# Alternative splicing is frequent during early embryonic development in mouse

**DOI:** 10.1186/1471-2164-11-399

**Published:** 2010-06-23

**Authors:** Timothée Revil, Daniel Gaffney, Christel Dias, Jacek Majewski, Loydie A Jerome-Majewska

**Affiliations:** 1 Department of Human Genetics, McGill University, Montreal, Quebec, Canada; 2 Genome Québec Innovation Centre, Montreal, Quebec, Canada; 3 Department of Pediatrics, Montreal Children's Hospital, Montreal, Quebec, Canada

## Abstract

**Background:**

Alternative splicing is known to increase the complexity of mammalian transcriptomes since nearly all mammalian genes express multiple pre-mRNA isoforms. However, our knowledge of the extent and function of alternative splicing in early embryonic development is based mainly on a few isolated examples. High throughput technologies now allow us to study genome-wide alternative splicing during mouse development.

**Results:**

A genome-wide analysis of alternative isoform expression in embryonic day 8.5, 9.5 and 11.5 mouse embryos and placenta was carried out using a splicing-sensitive exon microarray. We show that alternative splicing and isoform expression is frequent across developmental stages and tissues, and is comparable in frequency to the variation in whole-transcript expression. The genes that are alternatively spliced across our samples are disproportionately involved in important developmental processes. Finally, we find that a number of RNA binding proteins, including putative splicing factors, are differentially expressed and spliced across our samples suggesting that such proteins may be involved in regulating tissue and temporal variation in isoform expression. Using an example of a well characterized splicing factor, *Fox2*, we demonstrate that changes in *Fox2 *expression levels can be used to predict changes in inclusion levels of alternative exons that are flanked by Fox2 binding sites.

**Conclusions:**

We propose that alternative splicing is an important developmental regulatory mechanism. We further propose that gene expression should routinely be monitored at both the whole transcript and the isoform level in developmental studies

## Background

Developmental processes require precise spatial and temporal regulation of gene expression. Accordingly, developmental biologists have always been at the forefront of gene expression analysis, and recombinant DNA techniques such as transgenic and knockout models have greatly contributed to elucidation of developmental pathways and networks. Traditionally, these studies have focused on transcription factors and repressors that regulate the timing and strength of transcription. Recently, new regulatory mechanisms have emerged, such as post-transcriptional regulation by microRNAs and co-transcriptional regulation by alternative pre-mRNA splicing.

Alternative splicing is a pre-mRNA maturation process that consists of the removal or inclusion of certain alternative exons to produce different transcripts from one genomic locus [1,2]. Alternative splicing is now known to be prevalent in advanced eukaryotes. In humans, recent reports show that more than 98% of multi-exonic pre-mRNAs are alternatively spliced [3,4]. The mouse genome has been sequenced and, similarly to that of humans, a surprisingly low number of less than 30,000 genes have been identified [5,6]. It has been widely hypothesized that the great complexity of higher eukaryotic organisms stems from processes such as alternative splicing [7,8]. The distinct proteins translated from identical pre-mRNAs produced by this process can have different, even antagonistic activities. Thus, alternative splicing can play a major role in the activity of various important cellular mechanisms, such as cell differentiation, cell migration, cell growth and apoptosis. This wide range of cellular processes is required during mammalian embryogenesis to generate a viable organism from a single cell.

Several studies have suggested the importance of alternative splicing during development. In *C. elegans*, it was shown that 18% of the 352 verified alternative exons showed a larger than fourfold change in alternative splicing during its development from embryo to adult, including larval stages [9]. In humans, mice, chickens and *Xenopus*, a well-known example is provided by the fibroblast growth factor 8 (*FGF8*), which can produce many different isoforms [10]. Two of these, FGF8A and FGF8B, which differ by only eleven amino acids, have been shown to have different activities during development [11-14].

It has recently been shown that the levels of certain splicing factors, MBNL and CELF, are regulated and vary several-fold during pre- and postnatal heart development [15]. This variation in CELF and MBNL expression levels affects the splicing modulation of a large quantity of other alternative splicing events, suggesting the existence of a regulatory cascade at the splicing level. Some of the most interesting examples of alternative splicing and its regulation by splicing factors have been carried out in neural tissues. As neuronal precursor cells differentiate into neurons, there is a switch from the ubiquitous PTB to the highly similar, but neuron-specific, neural PTB (nPTB) [16]. As these two proteins modulate alternative splicing of specific subsets of pre-mRNAs, there is an associated switch of a large number of mRNA isoforms.

Since splicing factors may regulate alternative splicing of many different pre-mRNAs [17,18], knocking out known splicing factors in mice generally has profound effects on embryo or young pup viability. The majority of germ-line loss of function mutations in splicing factors result in embryonic arrest early in development, before embryonic day (E) 7.5, e.g. *Ptb *[19], *SC35 *[20,21], *Asf2/Sf2 *[22] and *SRp20 *[23]. In two cases, *Ptb *and *SRp20*, homozygous mutant embryos arrest at the morula stage [19,23]. *Prfp3 *mutant embryos also exhibit embryonic arrest, although it is not clear if these embryos die early or late in embryogenesis [24]. Germ-line loss of function mutations in splicing factors are also associated with organ specific abnormalities. For example, most *SRp38 *knockout embryos die before E15.5 with multiple cardiac defects [25], whereas a small number of mutant mice are born only to die soon after birth [22]. *Mbnl1 *and *Mbnl2 *are required in the skeletal muscle and eye [26,27], and Nova1 is required in motor neurons [20]. These studies indicate that there are stage-specific and tissue-specific requirements for splicing factors.

Although there have been a number of indications of the importance of alternative splicing in development, past studies have been limited largely to individual experiments focused on known candidate genes. In recent years, technological advances have paved the way to genome-wide analyses of mRNA processing, and have enabled hypothesis-free approaches. In this study, we take advantage of a splicing-sensitive exon microarray to investigate genome-wide variation in alternative splicing during development of the mouse embryo and its associated placenta. We focus on early developmental stages, E8.5-E11.5, in order to capture the isoform differences occurring during organogenesis. In mouse embryos, organogenesis begins at E8.5 and is mostly completed by E11.5 when most organs of the foetus can be recognized. The placenta is one of the first organs to form and function in the developing foetus. During organogenesis the precursors of the adult organs undergo a series of morphogenetic movements and differentiation events which have been shown to be controlled by changes in gene expression. We postulate that alternative splicing provides an additional mechanism for increasing the repertoire of transcripts from a limited number of developmentally relevant genes and confers additional specificity to individual developing tissues. Our results show that alternative splicing is frequent during organogenesis, and that different tissues (i.e. the placenta and embryo) as well as different developmental stages express specific gene isoforms. We confirm some previously described splicing events but also report numerous new time- and tissue-specific alternative isoforms. In addition, we find that the mRNA expression levels of some known splicing factors are modulated during organogenesis, suggesting that changes in expression levels of splicing factors may be responsible for alternative isoform expression.

## Results

**Figure 1:**
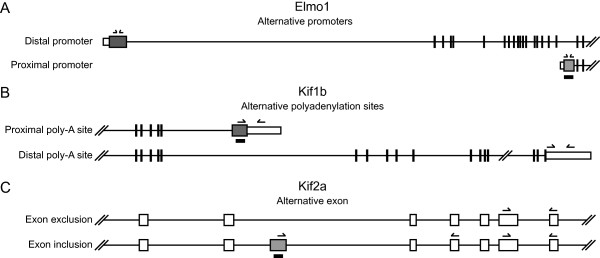
**Examples of types of alternative splicing analysed**. The boxes represent exons while the lines are introns. The bold lines beneath the exons indicate probes that detected significant expression changes for each example and the arrows represent primers used for qRT-PCR analyses. **(a) **Alternative promoter use can be found by an increase in the expression detection levels for a probe set for one promoter compared to the others. The change in promoter use of Elmo1 alters the 5' untranslated region (white boxes) as well as the N-terminus of the protein. **(b) **The same analyses can be applied for alternative polyadenylation sites, in this case Kif1b. **(c) **Alternative exons can be included or excluded in the mature mRNA, thus altering the coding sequence as in the Kif2a pre-mRNA.

We studied the variation in gene expression at the transcript isoform level in two "tissues" - embryonic and placenta - across three developmental stages: E8.5, E9.5, and E11.5. Unfortunately, we were not able to obtain sufficient amount of RNA from the chorion, the precursor of the placental tissue, at E8.5 and had to omit that sample from the analysis. For the remaining samples, 5 biological replicates were obtained for each tissue/stage, and the RNA from each replicate was hybridized to one Affymetrix GeneChip^® ^Mouse Exon 1.0 ST microarray. This array contains probes targeted to individual known and predicted exons, and allows monitoring of expression level at a sub-exon resolution. The replicate nature of the data allowed us to carry out analyses of variance (ANOVA) to detect differences in expression levels across time points and tissues. To investigate variations at the whole-transcript level, we performed the analysis using the summarized expression estimates of entire transcripts. For detection of differences in splicing and isoform expression, the expression levels of each individual probe set (roughly corresponding to an exon) were normalized by dividing by the expression level of the corresponding gene. The latter method will be referred to as the splicing index analysis. These two approaches allowed us to differentiate the cases where all, or most of the exons, within a gene have variable expression levels from the cases where only some of the exons within a transcript behave differently from the remaining exons, indicating alternative splicing or related isoform changes. This also allowed us to detect changes such as alternative transcription start sites or alternative polyadenylation sites which can also affect the N- or C-terminus of proteins (Figure 1).

### Data quality control and filtering

**Figure 2:**
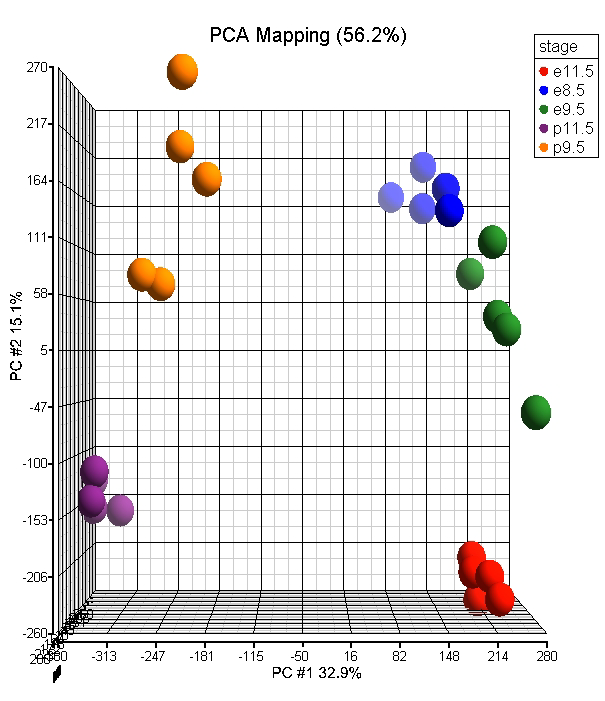
**Principal component analysis**. PCA was performed on the whole transcript levels. The plot shows that there is consistent behaviour (clustering) across biological replicates of the same samples. The two major sources of variation in the data are the tissue effect, which approximately corresponds to the 1^st ^principal component (horizontal axis), and the stage effect (vertical axis).

As the first quality control check, we carried out principal component analysis (PCA) to estimate the sources of variability within the data. PCA results from the whole-transcript analysis are shown in Figure 2, but similar trends are observed in exon-level, and normalized (Splicing Index) data. The first two principal components explain 48% of the variance of the data, and correspond to the tissue effect (32.9%) and stage effect (15.1%). Individual samples form distinct clusters, illustrating clear differences in gene expression levels across the two tissues and three stages.

We next carried out the splicing index analysis which identified a large number of candidate exons predicted to be alternatively spliced across the two tissues and three stages. Since it is instrumental to distinguish between truly alternatively spliced exons, and exons expressed at very low levels (indistinguishable from background noise) or very highly (beyond the sensitivity levels of the microarray); we employed a number of filtering steps, as described in Materials and Methods and previously in [28]. The Affymetrix Mouse Exon Array contains over 23,000 core meta probe sets (genes) and 280,000 probe sets (exons). Our filtering criteria reduced this number to 13,366 meta probe sets containing approximately 133,000 probe sets.

We investigated the efficacy of our data filtering steps by estimating the percentage overlap between candidate alternatively spliced exons in our study, with those predicted to be alternatively spliced based on independent EST and mRNA evidence such as those annotated by the "AltEvents" track from build 37 of the mouse genome on the UCSC Genome browser [5,29,30]. The Alt-Events track annotates alternative splice forms in UCSC "known" genes, which are assembled by combining evidence from multiple independent experimental sources. Application of our data filtering steps increases the percentage overlap with alternative events predicted from independent sources (Additional File 1 Figure S1). In addition, by increasing the stringency of the false discovery rate (FDR) cutoff we further increased the percentage overlap. This analysis demonstrates that the application of data filtering as well as the use of a stringent q-value cutoff increases the agreement between our results and alternatively spliced events predicted from independent experimental evidence. We note, however, that while our data filtering likely increases the sensitivity of our analysis, it is likely that the filters concurrently reduce specificity.

### Comparison of differentially expressed and spliced genes

To assess patterns of gene expression and splicing change over our entire data set, we defined a significant difference as a P-value which was less than that expected at an FDR of 0.05. Significant stage effects were defined using the combined P-values from the embryo and placenta: that is, a significant developmental stage effect reflects a difference in expression between different embryonic stages, placental stages or both. For brevity, we refer to differences in expression between embryo and placenta as "tissue-specific" and differences in expression between developmental stages as "stage-specific".

**Figure 3:**
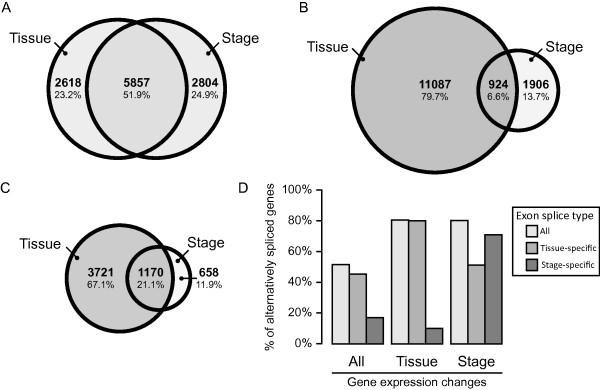
**Distribution of differentially expressed or spliced candidates**. (a) Number of genes that show significant expression changes between placental and embryonic tissues and between development stages, as well as the overlap between the two sets. (b) Individual probe sets that are differentially expressed compared to their transcripts between tissues and between stages. (c) Number of genes that contain at least one alternatively spliced exon when comparing embryo or placentas, or between developmental days. (d) Comparison of the percentage of tissue- or stage-dependent differentially alternatively spliced genes, or either, in three different subsets of differentially expressed genes: all, tissue-specific or stage-specific.

We first examined differences in expression between tissue and developmental stage at the level of whole gene expression. We find that approximately equal numbers of the 13,366 genes in our data set show a significant change in expression between day of development (8,661) as between placenta and embryo (8,475), with a large overlap between the two (5,857) (Figure 3A).

**Figure 4:**
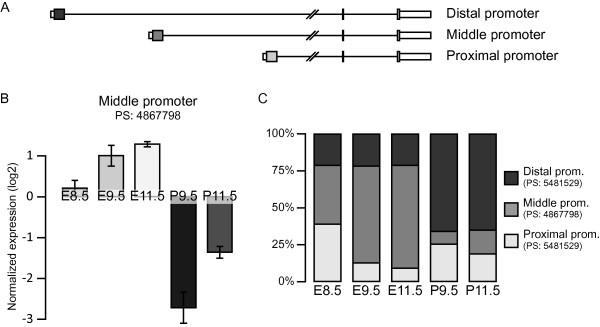
***Gcnt2 *promoter use during embryogenesis**. (a) Expression of the *Gcnt2 *gene is driven by three promoters, each contributing to different N-termini for the protein. (b) The use of the middle promoter is generally higher in the embryo than the placenta as detected by the probe set (PS) 4867798, and its use increases during development. (c) Comparison of the use of each three promoters, normalized to gene expression levels, shows a distinct profile for each promoter when comparing developmental stages and tissues.

We next examined stage and tissue differences in inclusion levels of individual exons. Of the nearly 300,000 core probe sets, 133,758 remained expressed at detectable levels after our filtering criteria. These probe sets are located in a total of 10,796 genes. We find that, at a FDR of 0.05, 12,011 (9%) probe sets are differentially included in their respective transcripts between embryo and placenta, while 2,830 (2%) are differentially included between day of development in either placenta or embryo, or both (Figure 3B). We also find numerous examples (924) where an exon is both tissue and stage-specific. For example, we observe an increase in the use of the middle promoter of *Gcnt2 *during embryo development, which in turn is higher than its use in placentas (Figure 4B). This seems to be due to changes in promoter use between tissues and stages (Figure 4C). Our results suggest that at least one putatively alternatively spliced exon is found in a total of 5,549 genes. A substantial fraction (21%) of these genes contains both a tissue-specific and stage-specific alternatively spliced exon (Figure 3C). In a majority of cases (824 genes), this is due to the alternative splicing of the same exon in a tissue and stage-specific manner.

We next investigated the overlap between whole gene expression change and alternative splicing. For clarity, in this section we focused on the 10,796 genes which contained at least one filtered (clearly expressed) probe set. We find that genes whose expression is significantly different between tissues or stages are highly enriched for alternatively spliced exons (Figure 3D). Genes that are differentially expressed between tissue are primarily enriched (compared to all genes) in tissue-specific alternative exons, while genes that are differentially expressed between developmental stages are likewise enriched in stage-specific alternative exons (Figure 3D). This analysis suggests that a large proportion of genes that are differentially expressed in early embryonic development concurrently exhibit significant isoform changes.

### Developmental processes and functions are overrepresented in alternatively spliced genes

To identify alternative isoforms in genes with a role in mammalian development, we used the simplified PANTHER gene ontology [31]. We tested for functional annotations that were enriched in the 1,828 genes with one or more developmentally-regulated alternative isoforms, compared to the expected distribution of annotation terms derived from a reference gene set. In our case, the reference set was comprised of all genes which we detected as expressed in the embryonic and placental samples. The results of this analysis (Tables 1 and 2) suggest that our candidate genes are active in important aspects of mammalian development. In particular, our candidate alternatively spliced genes appear to be active in cell attachment to the extracellular matrix, an integral process during organogenesis [32].

**Table 1:** Overrepresented PANTHER "Biological Process" terms.

PANTHER Biological Process	Observed	Expected	P-value
Cell adhesion	70	37.61	3.24 e-05

Developmental processes	218	160.69	8.49 e-05

Signal transduction	291	234.45	1.59 e-03

Cell adhesion-mediated signaling	43	21.22	3.61 e-03

Ectoderm development	80	50.05	6.14 e-03

Transport	130	94.69	6.45 e-03

Neurogenesis	70	44.34	3.52 e-02

**Table 2:** Overrepresented PANTHER "Molecular Function" terms.

PANTHER Molecular Function	Observed	Expected	P-value
Extracellular matrix structural protein	23	6.44	4.96 e-05

Cell adhesion molecule	44	23.42	2.36 e-03

Extracellular matrix	44	23.86	3.53 e-03

### Classification and visualization of alternative isoforms

**Figure 5:**
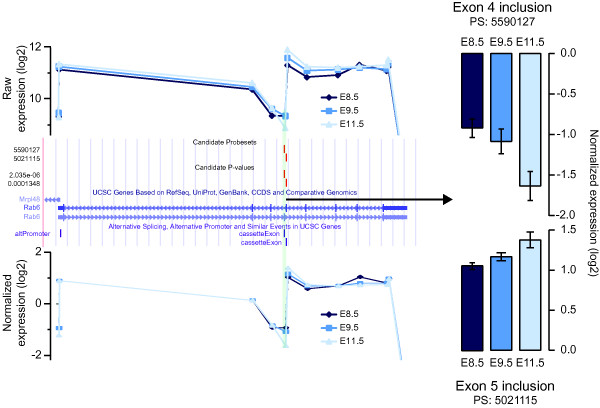
**Alternative splicing of *Rab6***. This pre-mRNA contains two mutually exclusive alternative exons, exons 4 and 5 (see middle panel representing the *Rab6 *gene structure using the UCSC Genome browser [29], http://genome.ucsc.edu). The top panel shows raw expression scores for each probe set for the three embryonic developmental stages. There is a slight difference in the expression of the entire transcript, as most probe sets increase their intensities from embryonic day (E) 8.5 to E11.5. This trend is not consistent for exons 4 and 5, as the inclusion of exon 4 actually goes down while the inclusion of exon 5 increases disproportionally. This is further emphasized in the bottom panel, showing the splicing index analysis and the expression values of each exon normalized to adjust for whole transcript expression changes. On the right, the bar graph shows the actual (log2 scale) reduction of exon 4 inclusion across the 3 day time span.

In order to classify, visualize, and evaluate the candidate alternative isoforms predicted by statistical analysis of exon microarray data, we created a database that links the results to the UCSC Genome Browser [29] and allows evaluation of each isoform in the context of all other available information, such as gene structure, expression information, as well as all other functional data provided by UCSC. The results are available in the additional data online (Additional file 2 Table S1). Figure 5 provides a concrete example of how microarray data and gene structure information can be combined to infer mutually exclusive alternative splicing of two neighbouring exons in the gene *Rab6*.

### Microarray results are confirmed by RT-PCR and qRT-PCR

The first level of validation of the microarray results was performed using end-point RT-PCR analysis. To simplify primer design and subsequent analysis, only alternative splicing events implicating cassette exons were verified using primers encompassing the alternative exons. Ten randomly chosen candidates with probe sets that overlapped at least partially with an existing cDNA, mRNA or EST-supported alternative exon, were selected (Table 3). All the ten exons verified were alternatively spliced in at least one of the sampled tissues (Additional file 3 Figure S2), and, in the cases where it could be quantified from the brightness of the corresponding bands in the gel, the trends of exon inclusion levels were in agreement with the microarray analysis.

**Table 3:** Validation of cassette exons by RT-PCR.

Gene symbol	Gene Name	P-value
Itga6	Integrin α 6	5.108 e-05

Erc1	ELKS/RAB6-interacting/CAST family member 1	4.412 e-04

Kif2a	Kinesin Family Member 2A	8.069 e-05

Epb4.1L3	Erythrocyte Protein Band 4.1-like 3	5.077 e-09

Numb	NUMB Gene Homolog	3.463 e-07

Pml	Promyelocytic Leukemia	5.884 e-06

Depdc5	DEP Domain Containing 5	9.024 e-06

Wnk1	WNK Lysine Deficient Protein Kinase 1	8.068 e-06

Mycbp2	MYC Binding Protein 2	8.881 e-06

Ganab	Alpha Glucosidase 2 Alpha Neutral Subunit	5.573 e-06

**Figure 6:**
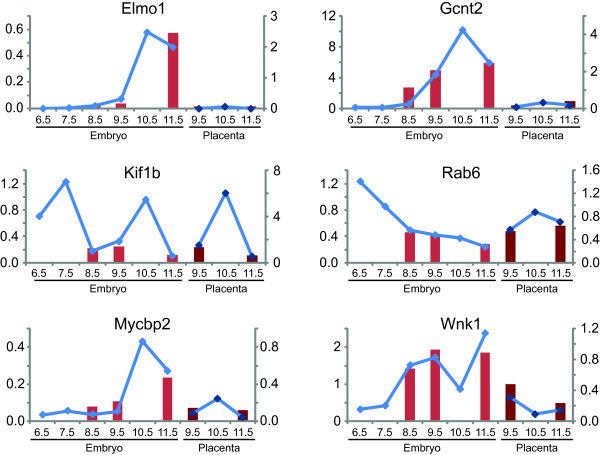
**Examples of qRT-PCR validation**. The results obtained from the microarray and quantitative RT-PCR follow the same trend, some of which are presented here. The bars (red) indicate the average of five probe set values of candidate exons divided by the average of the corresponding meta probe set values. The lines (blue) represent the fold change of the average of values for the qRT-PCR on the candidate exons as compared to internal controls on the same pre-mRNAs. Embryonic stages represented: E6.5 to E11.5; placental stages: P9.5 to P11.5.

To further verify the precise quantitative patterns of exon inclusion of the genes where the end-point quantification was not conclusive, we selected five of the already confirmed alternative cassette exons for analysis using quantitative real-time PCR. We also analysed seven additional alternative isoform events: four alternative promoters, two alternative polyadenylation sites, and one case of mutually exclusive exons (Table 4). We compared fold changes of the average of values for the qRT-PCR on the candidate exons with microarray probe set inclusion levels (see examples in Figure 6, compare blue lines with red bar graphs, respectively). The qRT-PCR based expression levels of all of these alternative splicing events displayed the same quantitative trend as earlier observed in the microarray analysis.

**Table 4:** Validation of different alternative splicing events by real-time PCR.

Gene symbol	Gene name	Splicing type	Microarray P-value
Elmo1	engulfment and cell motility 1, ced-12 homolog (C. elegans)	altPromoter	3.536 e-09

Ank3	ankyrin 3, epithelial	altPromoter	1.243 e-10

Gcnt2	glucosaminyl (N-acetyl) transferase 2, I-branching enzyme	altPromoter	4.825 e-09

Tcf4	transcription factor 4	altPromoter	1.173 e-08

Kif1b	kinesin family member 1B	altFinish	4.562 e-10

Itsn1	intersectin 1 (SH3 domain protein 1A)	altFinish	2.382e-07

Mycbp2	MYC binding protein 2	Cassette	8.881 e-06

Erc1	ELKS/RAB6-interacting/CAST family member 1	Cassette	4.412 e-06

Wnk1	WNK lysine deficient protein kinase 1	Cassette	8.068 e-06

Kif2a	kinesin family member 2A	Cassette	8.069e-05

Depdc5	DEP domain containing 5	Cassette	9.024e-06

Rab6	RAB6, member RAS oncogene family	Mutually exclusive	2.035e-06

In order to gain further insight into temporal variation in isoform expression, the qRT-PCR was performed on additional embryonic and placental stages, ranging from E6 to E11. The results of these additional stages were almost all predictable by extrapolation of the trends observed in the microarrays. A few demonstrated some unexpected behaviour, which may correspond to rapid changes in splicing patterns corresponding to developmental switches. As mice embryos develop extremely rapidly, with a gestation period of only 19-21 days, it is possible that the alternative splicing of certain pre-mRNAs can also be modified extremely rapidly.

The seventeen validated candidates are implicated in various cellular and organismal processes, and some have previously been shown to be involved in embryogenesis. For example, *Numb *is involved in neuronal differentiation during development. There are four known isoforms of *Numb*, two of which contain an alternative exon of 147 nt in the proline rich region and two without this exon [33]. These two classes have been shown to be differentially spliced during embryogenesis from E10 to E17, as well as in adults, to regulate cortical development [34]. This shift in alternative splicing is confirmed by our microarray results and we also find that this starts as soon as E8.5, the earliest data we have for this alternative splicing event.

### Multiple RNA-binding proteins are alternatively expressed and spliced during development

We examined our data set for differential expression and splicing of RNA-binding genes, since RNA-binding proteins (RBPs) may be involved in regulating tissue and temporal variation in isoform expression. Using a list of 380 putative RBPs recently published [35] as well as others added subsequently, we found that many RBPs are differentially expressed between the two tissues and three stages (Table 5). One interesting candidate is the alternative splicing factor CELF/BRUNOL4, which we observe to be regulated at both the expression and splicing levels. The global expression of this gene increases more than 2.5-fold in the embryo between E9.5 and E11.5. Meanwhile, in the same tissue samples and stages, the inclusion of exon 8 increases ten-fold and the use of a differential 3' end exon goes up 3.5 fold while in the placenta use of this 3' end exon goes down 4.5 fold between E9.5 and E11.5 (Additional file 2 Table S1). Although the consequence of this change of gene expression or alternative splicing is unknown, levels of CELF4/BRUNOL4 proteins are regulated during development of the brain and skeletal muscles between E14, newborn, postnatal day 4 and adult mice [36].

**Table 5:** Top 20 gene expression candidates in RNA binding proteins.

Gene symbol	Proposed functions (GO)	P-value of changes of gene expression	P-value of changes of alternative splicing
Slc6a1	Neurotransmitter transport	3.74 e-11	3.19 e-08

Dnajc6	Heat shock protein binding	2.35 e-10	1.27 e-04

Igf2bp1	mRNA stability	2.13 e-09	6.04 e-05

Enox1	Transport	6.26 e-09	1.53 e-04

Krr1	Ribosome biogenesis	6.38 e-09	N/A

Rbm13 (Mak16)	Ribosome biogenesis (yeast)	6.45 e-09	N/A

Ddx39	RNA splicing, RNA helicase	6.89 e-09	1.74 e-04

Nol9	Unknown	8.02 e-09	6.76 e-05

Zfp462	Transcription regulator (during development)	8.98 e-09	7.13 e-04

Nup37	Transport	1.86 e-08	N/A

Rbm9 (Fox-2)	RNA splicing	2.02 e-08	5.96 e-06

Pprc1	Transcription regulation	2.24 e-08	5.81 e-05

Pcbp3	Unknown	3.13 e-08	5.54 e-04

Elavl3	Cell differentiation, development	3.29 e-08	4.25 e-06

Mki67ip (Nifk)	Unknown	4.05 e-08	1.57 e-06

Rbms3	Unknown	7.05 e-08	N/A

Nup43	Transport	8.34 e-08	N/A

Rbm19	rRNA processing, ribosome biogenesis	8.34 e-08	N/A

Brunol4 (Celf4)	Splicing regulator	1.79 e-07	2.80 e-08

Eif3d	Translation initiation	2.28 e-07	7.34 e-04

**Figure 7:**
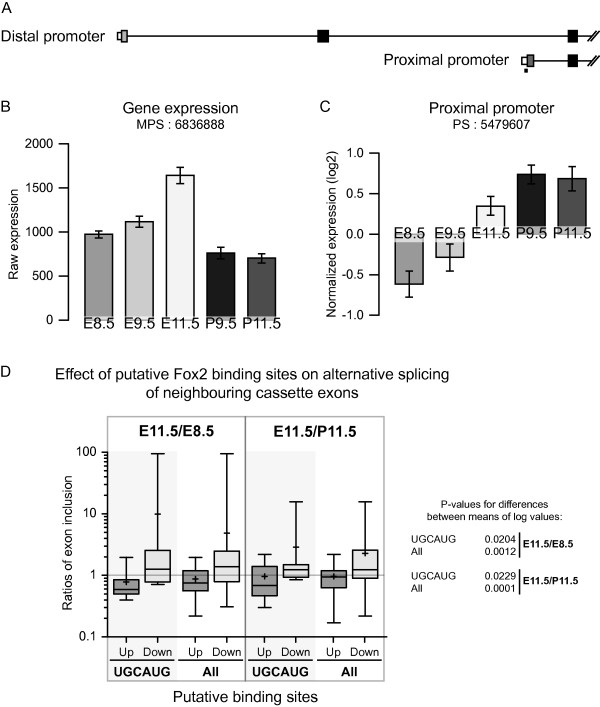
**The well characterized splicing factor Fox2 is alternatively expressed and spliced during development**. (a) Pre-mRNAs for this gene are transcribed using two primary promoters. (b) During development, the gene expression of *Fox2*, as detected by the meta probe set (MPS) 6836888 increases in embryos, while remaining generally lower and stable in placentas. (c) The use of the proximal promoter increases strongly during embryo development. (d) *Fox2 *expression levels modulate the alternative splicing of cassette exons containing putative *Fox2 *binding sites in their surrounding introns. The whiskers indicate the minimum and maximum value, the box represents the 25^th ^percentile to the 75^th^, the line inside the box shows the 50^th ^percentile and the + is the value of the mean. The strict UGCAUG binding site or a combination of 5 binding sites (All) were looked for within 100 nt of the splicing sites in the upstream (Up) or downstream (Down) introns. The E11.5/E8.5 or E11.5/P11.5 exon inclusion ratios of the candidates were calculated using corresponding probe set levels, normalized by meta probe set levels. A ratio over 1 indicates that inclusion of the alternative exon follows expression of *Fox2*, while a ratio less than 1 means that these are negatively correlated. Using a one-tailed, unpaired t-test, we find that the means of the log of the ratios are significantly different (P < 0.05) between Up and Down data sets.

Another particularly interesting candidate is *Rbm9*, also known as *Fox-2*. This protein is implicated in the regulation of alternative splicing in neurons and muscles [37] and has been shown as important for the survival of human embryonic stem cells [38]. According to our results, during embryogenesis, the expression levels of the isoforms in embryos nearly double from E8.5 to E11.5 (Figure 7B). Furthermore, the alternative splicing of the *Fox-2 *pre-mRNAs is significantly modified, with increasing use of the proximal promoter during development of both embryos and placentas, compared to E8.5 (Figure 7C). This leads to a different 5'UTR but also to a shorter N-terminal on the resulting protein. The effect of this switch of promoters is currently unknown but may play a role on the activity of this protein. Other exons of *Fox-2 *have also been demonstrated as alternatively spliced in later stages of development [15]. Between E14, postnatal and adult mice, exon 12 inclusion increases while exons 6 and 13 are excluded. This demonstrates a complex regulation of alternative splicing of this factor, with four exons modulated between E8.5 and postnatal life.

Because some aspects of the function of Fox2 and its binding sites within RNA sequences have been previously characterized, we were able to predict the effect of variation of Fox2 mRNA expression on a subset of exons that contain its putative binding sites. We compared the alternative splicing of cassette exons between E8.5 and E11.5 embryos as well as between E11.5 embryo and E11.5 placenta (P11.5), the two stages we observed the highest differences for *Fox2 *expression. The results obtained for these ratios (Figure 7D) demonstrate that inclusion rates of exons that are flanked by putative binding sites for Fox2 are highly correlated with the expression of this gene. The candidate exons were divided into two sets: those that contained a putative Fox2 binding site within the upstream intron, and those with a binding site in the downstream intron. Previous studies suggest that binding of Fox2 upstream of the exon should result in increased exon skipping, while downstream binding results in exon inclusion [38]. Our results indeed demonstrate that when the introns downstream of a cassette exon contained UGCAUG, a hexamer representing the most specific binding site for this protein, exon inclusion levels were positively correlated to *Fox2 *expression levels, while the opposite was true for exons with upstream binding sites. There is nearly a ten-fold difference between the means of exon inclusion ratios for these two categories, when comparing E11.5 and E8.5 (P = 0.0204). We also used a more relaxed criterion for identifying putative Fox2 binding sites, including all the binding sites previously described [38]. This analysis increased the sample size from 22 to 58 candidate regulated cassette exons (Additional file 4 Table S2), and resulted in increased statistical significance levels of the regulatory effect (P = 0.0012; Figure 7D, bars labelled "All"), suggesting that Fox2 binding sites may allow a level of degeneracy *in vivo*, and that there may be a large number of splicing events regulated by Fox2 during early embryonic development.

## Discussion

### Alternative splicing is frequent during early embryonic development

Alternative splicing is becoming a widely studied process that can increase the transcriptome complexity using a finite set of genes. However, little is known on the regulation of alternative splicing during development, where time and tissue specific regulation of protein levels is fundamental for cell differentiation, apoptosis and migration, all key regulators of organogenesis. In this work, we show that splicing is extensively regulated during organogenesis. We compile a list of 5,500 genes that indicate transcript isoform differences between our two tissues of interest - embryonic and placental - or across three developmental stages, E8.5, E9.5 and E11.5. According to a 5% FDR cutoff, we estimate that 9% of all exons are differentially alternatively spliced between the placenta and the embryo, and 2% of exons are differentially alternatively spliced across the three developmental stages. With 5 biological replicates each, and 6 distinct tissue/stage samples, our study has considerable statistical power to detect alternatively spliced transcripts. Our analysis also allows us to compare the fraction of genes that show isoform level changes with genes that are differentially expressed at the whole transcript level. Although we find that the number of differentially expressed genes is larger than differentially spliced, the numbers are of similar orders of magnitude (Figure 3). Interestingly, there is a large overlap between these subsets, and we show that genes with very significant levels of differential expression have a much higher than expected chance of being alternatively spliced.

It is also worth noting that because of the high statistical power of the study, our number of differentially expressed candidates is ten times higher than what was previously demonstrated by a genome-wide expression analysis of placentas and embryos at E12.5, which found only 6.5% of statistically significant differences in gene expression [39]. This could be explained in part by the advances in microarray analyses - i.e. the use of whole-transcript arrays - and the profiling of two placental stages allowing us to target a larger number of developmental events. However, it should be noted that the observed trends in expression are highly concordant between these two studies; most of the genes that had been shown to be highly expressed in placentas in the earlier study displayed the same high expression profile in our analysis.

Although we cannot rule out that a fraction of the genome-wide alternative splicing candidates may represent false positive results, our validation of 17 candidates presented here, as well as the 80% success in validation of previous experiments [40] suggests that false positives should not significantly affect our qualitative conclusions. It is difficult to estimate false negative rates in these types of studies. Although they are likely to be non-negligible, the limited power of the approach would render our estimates of the true frequency of alternative splicing conservative. We expect that this study exposes only the tip of the iceberg, and that a clearer picture will emerge as the whole-genome technologies mature. In the meantime, we provide a user-friendly database of our results in the additional material online (Additional file 1 Table S1).

### Implications for regulation of developmental pathways

In the current paradigm, regulation of developmental pathways is accomplished mainly at the level of transcription, and is mediated by changes in expression of certain key genes. For example, one of the earliest events in embryonic development is the separation of the inner cell mass - which forms the embryo proper - from the trophectoderm lineage. This event is regulated by interaction between three transcription factors: *Cdx2, Nanog*, and *Oct4*. This transcriptional regulation is essential for turning on numerous other genes that will drive differentiation of the embryo and its associated placenta. Incidentally, *Oct4 *is known to express multiple alternatively spliced isoforms with distinct expression patterns and possibly distinct functions [41]. In another well-known example, left-right asymmetry is governed by the *Nodal *molecular cascade. At E7.5 *Nodal *is turned on at the node, leading to auto regulation of its own expression in the left lateral plate, and turning on the expression of *Lefty *and *Pitx2 *genes. These three key players form the basis of a pathway containing numerous genes that control the specification of left versus right. Like *Oct4*, *Pitx2*, (one of our candidates, see Additional file 1 Table S1), expresses several isoforms with distinct transcriptional activities [42], and only one of those isoforms, *Pitx2c*, is required for left right asymmetry [43].

As the above examples demonstrate, many developmental pathways include alternative isoforms of key genes. Our own analysis presented here also indicates that a large proportion of developmentally regulated genes express alternative isoforms. The correct temporal and spatial expression of those isoforms is most likely regulated by specific splicing factors. Hence, we propose that in addition to canonical developmental pathways regulated by transcription factors, there exists a parallel, and yet highly overlapping, set of pathways regulated by splicing factors. A single splicing factor may affect the splicing of numerous exons in a large number of genes [18,44,45]. Thus, changes in expression levels of splicing factors can have profound downstream effects. Moreover, many splicing factors are themselves involved in self-regulatory feedback loops and express multiple isoforms [46,47]. Our analysis indicates differential embryonic expression of a number of RNA binding proteins and putative splicing factors. One of the particularly interesting examples is *Fox2 (RBM9 *or *Fxh*), a factor whose expression and promoter usage are both variable across the three developmental stages (Figure 7B). We find that *Fox2 *expression increases nearly 2 fold between days 8.5 and 11.5 in the embryo, but not in the placenta. We also show that this increase in *Fox2 *levels has a significant and predictable effect on the alternative splicing of cassette exons which contain putative binding sites within 100 nt in the neighbouring introns (Figure 7D). Furthermore, the use of the proximal promoter, which gives rise to an mRNA encoding a shorter N-terminus, increases between those stages (Figure 7C). Thus, not only is the overall amount of *Fox2 *product, but also the ratio of distinct isoforms, variable across early embryonic development.

Recently *Fox2 *has been found to be highly expressed in another developmentally relevant system, pluripotent cell lines, along with other pluripotency markers such as *Oct4 *and *Nanog*. *Fox2 *has been shown to regulate the splicing of numerous alternative exons in human embryonic stem cells, and to be necessary for maintaining viability of the cells. Moreover, *Fox2 *pre-mRNA contains active binding sites for its own protein, indicating a degree of autoregulation. Finally, many *Fox2 *targets are in turn splicing regulators, suggesting the existence of more extensive splicing networks [38]. The properties of such networks have been recently investigated in detail across a number of tissues and species [48].

### Future directions

Our study, along with a few recent publications [38,48-50] demonstrates that alternative splicing is a frequent event and is likely to have a significant role in development. With the promising results presented here, the future step will be to improve detection rates by using additional developmental stages, and a finer resolution of embryonic tissues. Indeed, the use of whole embryo mRNAs does not permit discovery of small-scale tissue-specific alternative splicing, which is likely to be extremely significant to tissue differentiation. Finally, most of our knowledge pertaining to genome-wide variation in alternative splicing has been acquired thanks to recent developments in microarray technologies, using either exon or splice-junction microarrays. Even more recent advances in high throughput sequencing will soon make it feasible to carry out whole-genome expression and isoform profiling experiments using mRNA sequencing. This approach allows digital monitoring of expression by counting the number of reads that map to mRNA segments of interest and comparing them across samples. These fragments may be genes, individual exons, or specific splice junctions. mRNA sequencing also allows the discovery of novel, as of yet unannotated, isoforms and transcripts. Preliminary studies carried out on a limited number of tissues and with limited sequencing coverage, [3,4] have already significantly enlarged our catalogue of alternatively spliced genes. With the rapidly increasing throughput, along with dropping costs and accessibility of sequencing, these technologies will soon allow us to routinely view gene expression at the sub exon level resolution and to decipher the role of splicing regulation in development and other systems.

## Conclusions

In this study, we have demonstrated that changes in alternative splicing are frequent during early mouse development between tissues and stages. Indeed, the numbers of differentially spliced genes are similar to those of differentially expressed genes. In these two categories, there is an overrepresentation of genes that have been implicated in development. In addition, many RNA-binding proteins are differentially spliced and/or expressed at the mRNA level. We can see a direct correlation between expression of Fox2, a known splicing factor, and the alternative splicing of cassette exons containing binding sites for this protein in the neighbouring introns. Thus, our results suggest that the effects of alternative mRNA isoforms should now be systematically verified in developmental gene studies.

## Methods

### Embryo collection

To generate embryos, C57BL/6J females were placed with C57BL/6J males overnight and checked for the presence of a vaginal plug in the morning. The day that a plug was detected was considered embryonic day (E) 0.5. All mice breeding and manipulations were performed in accordance with the Canadian Council on Animal Research. Embryos were collected between E6.5 - E11.5 for RNA isolation. Embryos for RNA isolation were stored in RNAlater (Ambion) for microarray analysis or TRIzol (Invitrogen) for RT-PCR, and isolated according to the manufacturers' protocol.

### Microarray Hybridization

The microarray hybridization and analysis was done as previously described [40], but using the Affymetrix GeneChip^® ^Mouse Exon 1.0 ST microarray. One microarray was used for each of the five biological replicates used in every stage and tissue analysed. All the microarray data has been deposited online in NCBI's Gene Expression Omnibus [51] with the accession number GSE21971. http://www.ncbi.nlm.nih.gov/geo/query/acc.cgi?acc=GSE21971.

### Normalization and summarization of ExonArray hybridization data

The Affymetrix Mouse ExonArray contains approximately 1.2 million probe sets which target roughly 1 million known and predicted exons. The annotations used to design these probe sets are derived from a variety of sources and vary dramatically in the strength of experimental evidence which supports their existence. These annotations are divided, in order of decreasing quality of experimental support into "core", "extended", "full", "free" and "ambiguous" annotations. Our analysis was restricted to the approximately 2.2 × 10^5 ^core probe sets on the ExonArray. These probe sets interrogate exons derived from RefSeq transcripts and/or full-length GenBank mRNAs. There are a number of issues to consider when analysing GeneChip data from an experiment with multiple arrays. These include background correction, normalization, non-specific hybridization and probe summarization. We background-corrected probe expression levels for non-specific binding based the distribution of binding intensities of a set of "anti-genomic" probes of a similar GC content to the probe of interest. These anti-genomic probes are designed so as to not hybridize with any sequence in the mouse genome and so provide an estimate of the level of non-specific binding for a given GC content. Probe intensities were then quantile normalized across all samples. Finally, individual probe expression levels were summarized into exon- and gene-level expression using the Probe Logarithmic Intensity Error (PLIER) algorithm. All analyses were performed using the Affymetrix Power Tools suite of command line programs [52].

### Data Quality Control

A major problem with inferring alternative splicing of exons between different biological samples using ExonArrays is differentiating such variation from changes in whole-gene expression level. The combination of changes in whole gene expression level with misleading probe set results can introduce potentially artefactual signals of alternative splicing in three main ways: via (i) the inclusion of probe sets from genes that are not expressed in a subset of samples (ii) the inclusion of cross-hybridizing probes or (iii) the inclusion of nonresponsive or "dead" probes [[53], Figure eight]. With these problems in mind we employed a number of quality control protocols to our expression data. Our objective was to minimize the number of false positive signals of differential splicing between our samples. Our filters are based upon the standards described by Affymetrix [53]. Firstly, in order to differentiate genic from exonic expression changes, all exon expression levels were normalized by the whole gene expression level estimated by the PLIER algorithm. Secondly, to minimize errors introduced by unexpressed genes, we also excluded all meta-probe sets and their constituent probe sets that were not expressed in all 25 of our samples. A gene was defined as "not expressed" if its mean expression level was lower than the quartile of the distribution of all core meta probe set intensities for the chip in question. Thirdly, we attempted to minimize the influence of cross hybridizing probe sets in our analysis by removing all probe sets in which the probe set/meta-probe set intensity ratio was greater than 5, indicating a high level of cross-hybridization. Finally, in order to remove "dead" probe sets we used the detection above background (DABG) P-value, as estimated by the apt-probeset-summarize program. The DABG P-value describes the probability that an intensity value at least as extreme as the observed could have been drawn from the null distribution, in this case the background distribution of intensity values. In order to account for chip-to-chip variation, we set a False Discovery Rate-corrected P-value threshold at 0.05 for each chip, based on the distribution of DABG values for that chip. In our case, a "dead" or unresponsive probe set was defined as any probe set in which the DABG P-value exceeded the chip-specific FDR threshold value in all samples. In order to stabilize the variance data values were log-transformed.

### Statistical Analysis

Each probe set in our filtered dataset retained 25 estimates of expression level. These estimates were divided by tissue (embryonic and placental) and developmental stage (days E8.5, E9.5 and E11.5 embryonic, days E9.5 and E11.5 placental) with 5 biological replicates in each tissue-stage. For simplicity, we refer to embryonic samples as a single "tissue" throughout. The absence of placental samples from day E8.5, due to insufficient RNA quantities, means that our dataset is unbalanced and therefore, not amenable to analysis by standard two-way ANOVA. Instead, we implemented the following approach. In order to test for significant differences in expression level between tissues we use a two-sample t-test, combining samples from day E9.5 and day E11.5 from both tissues. We tested for differences in expression level between developmental stages using a two sample t-test in placenta to compare expression level on day E9.5 and day E11.5, and using a one-way ANOVA in embryo to compare expression levels on days E8.5, E9.5 and E11.5. The p-values for these latter two tests were then combined using Fisher's method [54] under the common null hypothesis of no significant variation in expression level across developmental stages in either tissue. All p-values were adjusted for multiple testing by using the Benjamini and Hochberg [55] FDR procedure, and a false discovery rate of 0.05 was used as a cutoff for reporting the results.

### Fox2 effect on alternative cassette exon inclusion

In order to confidently identify cassette exons, of the top 2830 significant probe sets (Additional file 1 Table S1), we selected those that were annotated as "cassetteExon" in the "knownAlt" track on the UCSC Genome Browser [30]. As binding sites for Fox2 are found predominantly in the surrounding introns within 100 nt of the splicing sites [38], upstream and downstream introns were scanned for the presence of several different putative binding sites previously published [38], using sequences extracted with the UCSC Table Browser data retrieval tool [56]. We then calculated E11.5/E8.5 and E11.5/P11.5 ratios for each probe set value, normalized to the corresponding meta probe set. This ratio corresponds to the predicted ratio of exon inclusion levels. The candidate exons were divided into two sets: those that contained a putative Fox2 binding site within the upstream intron, and those with a binding site in the downstream intron. The data was log-transformed in order to stabilize the variance of the ratios, and the differences between the two sets were analysed using a one-tailed, unpaired Welch's t test [57]. The results were considered significant if the p-values for the difference between the means were less than 0.05 (P < 0.05).

### End-point PCR validation

Standard PCR validation was done using HotStarTaq PCR mix (Qiagen) according to the manufacturer's instructions. 8 ng of cDNA were used in a 10 μl reaction with conditions set as follows: 15 minutes at 95°C, followed by 30 cycles of 30 seconds at 95°C, 30 seconds at 58°C and 45 seconds at 72°C. The resulting products were analyzed on a 2% agarose gel (Additional file 3 Figure S2). Primer sequences used are available in additional table (Additional file 4 Table S3).

### Real-time PCR validation

The validation of the results by qPCR was done in a 384-well format using the 7900HT Fast Real-Time PCR System (Applied Biosystems) and the Power SYBR^® ^Green PCR Master Mix (Applied Biosystems) according to the manufacturer's protocol. The concentrations of the reagents used were 8 ng of cDNA was in a 10 μl reaction with 320 nM of each primer. The parameters of the qRT-PCR machine were 10 minutes at 95°C, followed by 40 cycles of 20 seconds at 95°C, 30 seconds at 58°C and 45 seconds at 72°C. The primers used are shown in additional table (Additional file 4 Table S4).

## Abbreviations

DABG: Detection Above BackGround; DAS: Differentially Alternatively Spliced; E: Embryonic day; FDR: False Discovery Rate; MPS: Meta Probe Set; PCA: Principal Component Analysis; PS: Probe Set; RBP: RNA-Binding Protein

## Authors' contributions

DG performed the data analyses. TR contributed to the data analyses and validated results. CD prepared the samples for microarray experiments and participated in validation of the results. LJM collected and provided the samples used for the study. JM and TR drafted the manuscript. JM and LJM conceived and co-ordinated the study. All authors read and approved the final manuscript.

## Supplementary Material

Additional file 1 **Figure S1 - Data filtering**. Fold-change in percentage overlap of predicted alternatively-spliced exons in our analysis with alternatively spliced events based on UCSC "known" genes versus False Discovery Rate (FDR) P-value threshold. Click here for file

Additional file 2 **Table S1 - Candidates**. A list of all significant alternatively spliced candidates found during our analysis. Click here for file

Additional file 3 **Figure S2 - PCR validation**. Results of the endpoint PCR validation of alternative cassette exons. Click here for file

Additional file 4 **Additional tables **. **Table S2 **Table containing primers used for end-point PCR validation. **Table S3 **Table containing primers used for qRT-PCR validation. **Table S4 **Information about binding sites found for Fox2. Click here for file
