# Supplementary material for: Alternative splicing is frequent during early embryonic development in mouse
Source: BMC Genomics. 2010 Jun 23;11:399. doi: 10.1186/1471-2164-11-399 (PMC2898759; doi:10.1186/1471-2164-11-399)
Supplement: Additional file 1 — Figure S1 - Data filtering. Fold-change in percentage overlap of predicted alternatively-spliced exons in our analysis with alternatively spliced events based on UCSC "known" genes versus False Discovery Rate (FDR) P-value threshold. [file 1471-2164-11-399-S1.PDF]

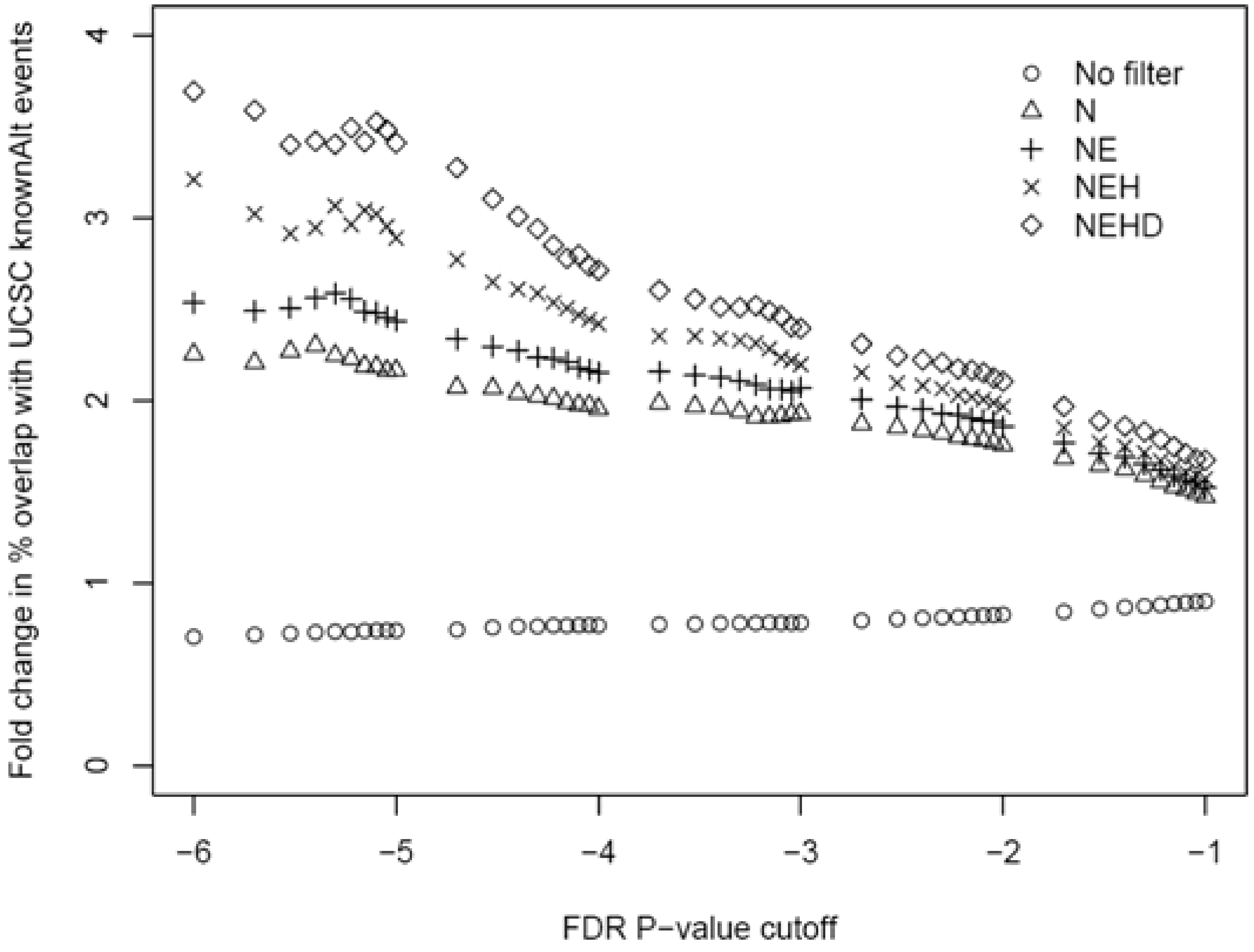

Supplemental figure 1 – Fold-change in percentage overlap of predicted alternatively-spliced exons in our analysis with alternatively spliced events based on UCSC "known" genes versus False Discovery Rate (FDR) P-value threshold. Here, a predicted alternatively spliced exon was any exon where the P-value associated with either a tissue or developmental stage effect was less than a given FDR threshold value. The fold-change in overlap is relative to the background overlap of all exons in a filtered dataset with the UCSC annotation. The filters are: N - exon expression normalized by whole gene expression level; NE - N + "unexpressed" genes removed; NEH - NE + potentially cross-hybridizing probe sets removed; NEHD - NEH + putatively "dead" probes removed. After expression-level filtering, at the FDR rate of 0.05, our results contain twice as many exons that already have prior evidence of alternative splicing as would be expected by chance.
