# Supplementary material for: Alternative splicing is frequent during early embryonic development in mouse
Source: BMC Genomics. 2010 Jun 23;11:399. doi: 10.1186/1471-2164-11-399 (PMC2898759; doi:10.1186/1471-2164-11-399)
Supplement: Additional file 2 — Table S1 - Candidates. A list of all significant alternatively spliced candidates found during our analysis. [file 1471-2164-11-399-S2.HTML]

AltDev: Alternatively spliced during development

# Supplemental data

### Top 2830 significant probe sets in 1828 meta probe sets (genes), with a P-value range of 2.89013e-12 to 1.05753e-03. Only the probe sets labeled as "core" are included. PLIER summarization was applied as discussed in the text.**| Gene (Entrez): | Corresponding "NCBI Entrez" profile. || Meta probe set (UCSC): | UCSC gene track containing customized probe set tracks. || Gene intensities: | Figures representing individual probe set intensites for each meta probe set, both raw and normalized. || Probe set intensities: | Bar graphs of the normalized expression of individual candidate probe sets, representing || | known (overlapping the UCSC knownAlt track; in green) and novel events (in red) | | --- | --- | --- | --- | --- | --- | --- | --- | --- | --- | | Rank | Gene (Entrez) | Combined P Value | Metaprobeset (UCSC) | Gene Intensities | Probeset Intensities | | --- | --- | --- | --- | --- | --- | | 1. | Scarb1 | P < 2.890e-12 | 6941934 | 6941934 | 4660928 (2.89e-12) | 5493341 (9.71e-07) | 5521512 (1.11e-04) | 5013866 (6.77e-04) | | 2. | Srpk2 | P < 5.122e-11 | 6936679 | 6936679 | 5170316 (5.12e-11) | 5062089 (7.41e-06) | 4701023 (1.04e-05) | 4900774 (6.00e-05) | 4318037 (1.89e-04) | | 3. | Ank3 | P < 1.243e-10 | 6768609 | 6768609 | 4712739 (1.24e-10) | 4719820 (2.43e-06) | 5599187 (1.22e-05) | 5074796 (1.85e-05) | 4576825 (1.85e-05) | 5217758 (7.72e-05) | 5224672 (2.19e-04) | 5049028 (2.28e-04) | 5375456 (2.93e-04) | | 4. | Dab2 | P < 1.347e-10 | 6828472 | 6828472 | 5480180 (1.35e-10) | 5371937 (8.37e-04) | | 5. | Slc12a6 | P < 1.417e-10 | 6880035 | 6880035 | 4340349 (1.42e-10) | 4709104 (4.12e-10) | 4424605 (7.15e-08) | 5016804 (1.96e-07) | 4322175 (6.01e-06) | 4328664 (7.63e-05) | 5177065 (7.76e-05) | 4948957 (2.54e-04) | 5358690 (3.39e-04) | 5035715 (3.90e-04) | 5260005 (3.93e-04) | | 6. | Slc2a3 | P < 1.809e-10 | 6957051 | 6957051 | 4508655 (1.81e-10) | 4916766 (2.24e-09) | 4512524 (2.62e-09) | 4540788 (3.23e-05) | 4967908 (3.30e-05) | 4479978 (1.50e-04) | | 7. | Nr6a1 | P < 2.199e-10 | 6886183 | 6886183 | 5587545 (2.20e-10) | 4656323 (6.79e-10) | 4774490 (2.74e-07) | 5551552 (5.32e-07) | 4648147 (3.00e-06) | 4808440 (5.50e-05) | 5220726 (4.32e-04) | 4900823 (5.55e-04) | | 8. | Kif1b | P < 4.562e-10 | 6926938 | 6926938 | 4949995 (4.56e-10) | 5596673 (1.56e-07) | 4420256 (4.84e-07) | 5125179 (1.56e-06) | 5476790 (3.75e-06) | 4630160 (5.09e-06) | 4861196 (5.53e-06) | 4738866 (8.19e-06) | 4371550 (3.55e-05) | 4602524 (5.40e-05) | 4686754 (8.89e-05) | 4867150 (9.49e-05) | 4741836 (1.11e-04) | 4392857 (1.51e-04) | 4995001 (1.63e-04) | 5460841 (1.85e-04) | 4521629 (2.47e-04) | 4755926 (3.05e-04) | 5061402 (4.23e-04) | 5103383 (5.30e-04) | 4743657 (8.63e-04) | 4371388 (8.83e-04) | | 9. | Lin28 | P < 6.014e-10 | 6925983 | 6925983 | 4322713 (6.01e-10) | 4936195 (1.09e-06) | 5616296 (3.08e-04) | 4607280 (7.64e-04) | | 10. | NA | P < 1.152e-09 | 6994688 | 6994688 | 5289170 (1.15e-09) | 4644167 (2.98e-08) | 4772751 (3.69e-08) | 4711853 (1.98e-07) | 5444577 (8.34e-06) | 5114272 (1.06e-05) | 5416277 (4.41e-05) | 5430992 (1.36e-04) | 5555498 (3.94e-04) | | 11. | Dner | P < 1.924e-09 | 6760292 | 6760292 | 4332294 (1.92e-09) | 4895536 (3.70e-09) | 5256194 (4.31e-05) | 5098108 (2.24e-04) | | 12. | Fbxl10 | P < 1.950e-09 | 6941768 | 6941768 | 4828148 (1.95e-09) | 4624773 (1.20e-08) | 4417537 (5.55e-08) | 4451074 (1.00e-07) | 5494893 (1.78e-07) | 5011660 (8.62e-07) | 5368455 (2.06e-06) | 5225834 (3.66e-06) | 4911144 (3.89e-06) | 4699986 (1.44e-05) | 4544136 (1.70e-05) | 4711531 (2.09e-05) | 5098311 (4.04e-05) | 5326373 (4.08e-05) | 5034426 (1.05e-04) | 4328332 (1.21e-04) | 4608244 (1.77e-04) | 5057139 (3.46e-04) | | 13. | Enpp2 | P < 1.980e-09 | 6835759 | 6835759 | 4429017 (1.98e-09) | 4741769 (5.43e-04) | | 14. | Cldn7 | P < 2.749e-09 | 6782087 | 6782087 | 5067524 (2.75e-09) | | 15. | E130012A19Rik | P < 3.053e-09 | 6791207 | 6791207 | 4490352 (3.05e-09) | | 16. | Mtvr2 | P < 3.220e-09 | 6871139 | 6871139 | 4511546 (3.22e-09) | 4852789 (3.91e-08) | 4334832 (2.54e-06) | 5318824 (2.80e-04) | 4649519 (7.45e-04) | | 17. | Elmo1 | P < 3.536e-09 | 6805158 | 6805158 | 5166975 (3.54e-09) | 4596721 (3.79e-09) | 4554022 (1.40e-08) | 5001412 (5.01e-08) | 4368163 (3.61e-06) | 4376542 (1.31e-05) | 4651632 (3.10e-05) | 5158535 (2.43e-04) | | 18. | Atp2b4 | P < 3.681e-09 | 6762321 | 6762321 | 4426241 (3.68e-09) | | 19. | Tcf4 | P < 4.173e-09 | 6861850 | 6861850 | 5205633 (4.17e-09) | 4886763 (1.17e-08) | 5422870 (1.05e-03) | | 20. | Gcnt2 | P < 4.825e-09 | 6806435 | 6806435 | 4867798 (4.83e-09) | 5600462 (4.48e-04) | | 21. | NA | P < 4.910e-09 | 6754519 | 6754519 | 5206863 (4.91e-09) | 4640153 (5.41e-06) | 4793245 (6.19e-04) | 5061812 (6.65e-04) | 4390282 (9.83e-04) | | 22. | Epb4.1l3 | P < 5.077e-09 | 6851897 | 6851897 | 5517310 (5.08e-09) | 5169699 (4.48e-06) | 4866829 (4.80e-04) | 5076503 (6.30e-04) | | 23. | Rtn4 | P < 7.722e-09 | 6779725 | 6779725 | 4726341 (7.72e-09) | 5252456 (5.23e-07) | 5103965 (1.54e-05) | | 24. | Vwf | P < 8.466e-09 | 6949884 | 6949884 | 4445014 (8.47e-09) | 4406809 (7.93e-07) | 5293344 (1.01e-05) | 5134551 (4.46e-05) | 4742462 (1.07e-04) | 5387652 (2.24e-04) | 4496265 (2.42e-04) | 4375079 (2.58e-04) | 4884257 (6.92e-04) | | 25. | Gas6 | P < 1.249e-08 | 6980568 | 6980568 | 5127226 (1.25e-08) | 5043840 (5.20e-04) | 5583070 (8.91e-04) | | 26. | Spnb2 | P < 1.259e-08 | 6786978 | 6786978 | 4684563 (1.26e-08) | 5559217 (9.38e-08) | 4931051 (2.30e-06) | 5270878 (1.51e-04) | 5305825 (1.71e-04) | 4763474 (5.06e-04) | | 27. | Ppfibp2 | P < 1.310e-08 | 6963271 | 6963271 | 4655793 (1.31e-08) | 4866715 (1.37e-07) | 5513701 (9.49e-05) | 5387620 (1.48e-04) | | 28. | NA | P < 1.326e-08 | 6907289 | 6907289 | 4898548 (1.33e-08) | | 29. | Pcdha1 | P < 1.525e-08 | 6860138 | 6860138 | 5290439 (1.53e-08) | 4970455 (5.44e-06) | 4981076 (9.06e-06) | 5504302 (1.39e-05) | 4899198 (2.08e-05) | 5510874 (2.75e-05) | 5439817 (2.81e-05) | 4785195 (4.75e-05) | 4333002 (1.83e-04) | 5329487 (3.71e-04) | 4693451 (7.11e-04) | 5070058 (9.07e-04) | 4618635 (9.32e-04) | | 30. | Fzd1 | P < 1.612e-08 | 6935966 | 6935966 | 5609113 (1.61e-08) | | 31. | Sp5 | P < 1.671e-08 | 6877958 | 6877958 | 5603560 (1.67e-08) | 4622564 (1.01e-06) | | 32. | Tubg2 | P < 1.789e-08 | 6784245 | 6784245 | 5560035 (1.79e-08) | 4457097 (3.14e-06) | 5413629 (9.28e-04) | | 33. | Mapk8 | P < 2.034e-08 | 6823849 | 6823849 | 4455656 (2.03e-08) | | 34. | Anxa8 | P < 2.096e-08 | 6818186 | 6818186 | 4405609 (2.10e-08) | 5187505 (3.94e-06) | 4877445 (1.78e-05) | 5390328 (4.30e-05) | | 35. | Myod1 | P < 2.291e-08 | 6960454 | 6960454 | 4666481 (2.29e-08) | 4860535 (2.19e-05) | | 36. | Brunol4 | P < 2.797e-08 | 6864062 | 6864062 | 4596487 (2.80e-08) | 5520202 (4.36e-05) | | 37. | Hoxd11 | P < 3.020e-08 | 6878261 | 6878261 | 4803984 (3.02e-08) | 5396842 (8.96e-08) | 5297562 (1.95e-05) | | 38. | Abce1 | P < 3.099e-08 | 6983624 | 6983624 | 4394518 (3.10e-08) | 4933974 (6.06e-04) | | 39. | Slc6a1 | P < 3.193e-08 | 6949153 | 6949153 | 4545950 (3.19e-08) | 5465561 (4.59e-06) | 4939010 (5.35e-06) | 4472248 (9.61e-06) | 5107792 (4.11e-05) | 4780523 (4.00e-04) | | 40. | Nkx3-1 | P < 3.320e-08 | 6820018 | 6820018 | 5494530 (3.32e-08) | | 41. | Pstpip1 | P < 3.583e-08 | 6989294 | 6989294 | 4896054 (3.58e-08) | | 42. | Cux1 | P < 3.735e-08 | 6942496 | 6942496 | 4991090 (3.74e-08) | 4581201 (1.00e-05) | 4901872 (4.33e-05) | 4851097 (5.38e-05) | 5018323 (8.78e-04) | | 43. | Atp1a3 | P < 3.828e-08 | 6965901 | 6965901 | 4501569 (3.83e-08) | 5027451 (5.81e-08) | 4968020 (8.40e-08) | 4399944 (1.93e-07) | 4606534 (5.90e-07) | 4758610 (1.11e-06) | 5418296 (1.18e-05) | 4684690 (1.71e-05) | 4584772 (4.55e-05) | 4404824 (2.07e-04) | 5528785 (1.01e-03) | | 44. | Inpp5f | P < 4.711e-08 | 6964428 | 6964428 | 4414633 (4.71e-08) | 5432381 (2.07e-06) | 4383534 (5.17e-05) | 5595921 (1.55e-04) | 5132656 (2.12e-04) | 5307934 (5.00e-04) | 5027613 (6.36e-04) | | 45. | Col5a2 | P < 4.813e-08 | 6758414 | 6758414 | 5051652 (4.81e-08) | 4664898 (5.35e-08) | 5226364 (2.81e-04) | | 46. | Ttyh1 | P < 5.402e-08 | 6972710 | 6972710 | 4596170 (5.40e-08) | 4930647 (8.45e-07) | 5266108 (7.25e-06) | 5549432 (6.21e-05) | | 47. | Slc2a2 | P < 6.099e-08 | 6896428 | 6896428 | 5033193 (6.10e-08) | 4829988 (5.76e-04) | | 48. | Slc6a4 | P < 6.530e-08 | 6782554 | 6782554 | 4372332 (6.53e-08) | 4925107 (1.48e-06) | 4539034 (2.39e-04) | | 49. | Scrt1 | P < 6.558e-08 | 6836830 | 6836830 | 5562773 (6.56e-08) | 5610215 (6.43e-04) | | 50. | Ndrg4 | P < 6.568e-08 | 6978390 | 6978390 | 5206768 (6.57e-08) | 5368891 (2.80e-06) | 4484264 (8.23e-04) | | 51. | Mttp | P < 6.635e-08 | 6909796 | 6909796 | 5319297 (6.63e-08) | | 52. | Dnmt3a | P < 7.027e-08 | 6792945 | 6792945 | 5035195 (7.03e-08) | 4810423 (2.09e-07) | 4523838 (1.37e-06) | 5505365 (4.58e-05) | 5613482 (1.37e-04) | 4937484 (1.67e-04) | 5379771 (2.21e-04) | 4513447 (2.37e-04) | 4358921 (2.71e-04) | 5439439 (5.66e-04) | 5465781 (6.33e-04) | | 53. | Pcaf | P < 1.061e-07 | 6851103 | 6851103 | 5219747 (1.06e-07) | 4556840 (3.14e-04) | | 54. | Sgk1 | P < 1.066e-07 | 6766455 | 6766455 | 5463222 (1.07e-07) | 4545625 (1.26e-07) | 4305308 (1.77e-07) | 5503524 (2.13e-05) | 5440065 (8.35e-05) | 5433118 (1.32e-04) | | 55. | Rhpn2 | P < 1.091e-07 | 6959807 | 6959807 | 4312512 (1.09e-07) | 4312198 (8.15e-05) | 5600657 (2.06e-04) | | 56. | Dok4 | P < 1.141e-07 | 6984485 | 6984485 | 5305013 (1.14e-07) | | 57. | Nr1d2 | P < 1.168e-07 | 6822946 | 6822946 | 4329889 (1.17e-07) | | 58. | Fbln2 | P < 1.197e-07 | 6947987 | 6947987 | 4444116 (1.20e-07) | 5564000 (6.17e-04) | | 59. | Mybbp1a | P < 1.292e-07 | 6782248 | 6782248 | 4741724 (1.29e-07) | 5078048 (1.85e-04) | 4563208 (2.32e-04) | 5464735 (7.97e-04) | | 60. | Ttc9c | P < 1.375e-07 | 6871380 | 6871380 | 4796433 (1.37e-07) | | 61. | Vwa1 | P < 1.412e-07 | 6927298 | 6927298 | 5133612 (1.41e-07) | 5329684 (4.58e-04) | | 62. | Sox8 | P < 1.532e-07 | 6854425 | 6854425 | 4607317 (1.53e-07) | | 63. | Spock2 | P < 1.556e-07 | 6768151 | 6768151 | 5039738 (1.56e-07) | 5479179 (8.46e-07) | 4610840 (1.45e-04) | 4604316 (1.89e-04) | | 64. | Trip10 | P < 1.578e-07 | 6851320 | 6851320 | 4346889 (1.58e-07) | 5423530 (5.47e-06) | 4337430 (2.16e-04) | | 65. | Hcfc1r1 | P < 1.663e-07 | 6849215 | 6849215 | 5088495 (1.66e-07) | 5522313 (1.72e-05) | | 66. | Car4 | P < 1.776e-07 | 6783144 | 6783144 | 4534935 (1.78e-07) | 5511019 (4.92e-04) | | 67. | B930006L02Rik | P < 1.793e-07 | 6962961 | 6962961 | 4464127 (1.79e-07) | 4892091 (7.77e-04) | | 68. | Rarg | P < 1.907e-07 | 6838717 | 6838717 | 5415271 (1.91e-07) | 5514861 (6.22e-04) | | 69. | Cyp4b1 | P < 1.972e-07 | 6924721 | 6924721 | 4560273 (1.97e-07) | | 70. | Arhgef2 | P < 2.112e-07 | 6899148 | 6899148 | 4917866 (2.11e-07) | | 71. | Slc7a7 | P < 2.128e-07 | 6824728 | 6824728 | 5348360 (2.13e-07) | | 72. | Mtap1b | P < 2.234e-07 | 6815490 | 6815490 | 5040788 (2.23e-07) | | 73. | Itsn1 | P < 2.382e-07 | 6843198 | 6843198 | 5598999 (2.38e-07) | 5115644 (1.90e-06) | 5484437 (5.18e-06) | 4953041 (1.32e-05) | 4906311 (2.79e-05) | 4460314 (7.17e-05) | 4534254 (2.57e-04) | | 74. | Reep6 | P < 2.637e-07 | 6769212 | 6769212 | 4842548 (2.64e-07) | 4861381 (1.04e-03) | | 75. | Amn | P < 2.711e-07 | 6798197 | 6798197 | 5164607 (2.71e-07) | 4728219 (8.22e-06) | 4431764 (2.87e-05) | | 76. | Col25a1 | P < 2.762e-07 | 6901347 | 6901347 | 5328735 (2.76e-07) | 4767450 (1.64e-04) | 5262978 (5.04e-04) | | 77. | Lgmn | P < 2.872e-07 | 6803161 | 6803161 | 5058373 (2.87e-07) | 4801565 (1.05e-04) | | 78. | Fyn | P < 3.028e-07 | 6767258 | 6767258 | 5066773 (3.03e-07) | | 79. | Pla2g7 | P < 3.163e-07 | 6850534 | 6850534 | 4480943 (3.16e-07) | | 80. | Atp2b2 | P < 3.164e-07 | 6956592 | 6956592 | 5139170 (3.16e-07) | 4732579 (6.18e-07) | 4371533 (9.74e-06) | 4687377 (2.30e-05) | 4492127 (2.88e-04) | 5024934 (5.40e-04) | | 81. | Igf2r | P < 3.316e-07 | 6853780 | 6853780 | 5502271 (3.32e-07) | 4821702 (6.57e-05) | 4450896 (2.21e-04) | 4648111 (8.74e-04) | | 82. | Evi5l | P < 3.340e-07 | 6973718 | 6973718 | 4855752 (3.34e-07) | 4996325 (1.65e-05) | | 83. | Rbp4 | P < 3.424e-07 | 6872980 | 6872980 | 5219209 (3.42e-07) | 4434196 (2.54e-04) | 4458069 (2.96e-04) | | 84. | Numb | P < 3.463e-07 | 6802290 | 6802290 | 5476023 (3.46e-07) | | 85. | Antxr1 | P < 3.497e-07 | 6955259 | 6955259 | 5491857 (3.50e-07) | 5357389 (2.79e-06) | | 86. | Adcyap1 | P < 3.570e-07 | 6853121 | 6853121 | 4366556 (3.57e-07) | 4807173 (7.59e-06) | 5235319 (2.18e-04) | | 87. | Sorbs3 | P < 3.889e-07 | 6825684 | 6825684 | 4394107 (3.89e-07) | 5258790 (4.04e-07) | 5135300 (1.51e-06) | 5280148 (4.39e-05) | 4486168 (6.07e-05) | 4993284 (1.04e-04) | 5225853 (5.06e-04) | | 88. | Gsn | P < 4.033e-07 | 6876380 | 6876380 | 5075084 (4.03e-07) | 4712316 (5.74e-04) | | 89. | Thra | P < 4.039e-07 | 6784042 | 6784042 | 4582355 (4.04e-07) | | 90. | Mtus1 | P < 4.129e-07 | 6981905 | 6981905 | 5247471 (4.13e-07) | 5023875 (1.39e-06) | 5473544 (1.62e-05) | 4786129 (1.70e-05) | 4810352 (1.49e-04) | 4479449 (3.13e-04) | | 91. | Srpx | P < 4.397e-07 | 7015549 | 7015549 | 5149956 (4.40e-07) | 5228085 (1.83e-05) | 5098258 (9.05e-05) | | 92. | Nedd4l | P < 4.851e-07 | 6861576 | 6861576 | 4836449 (4.85e-07) | | 93. | Map3k7 | P < 4.968e-07 | 6912461 | 6912461 | 4864482 (4.97e-07) | | 94. | Dis3 | P < 5.091e-07 | 6826927 | 6826927 | 5599846 (5.09e-07) | 5608937 (1.02e-06) | 4715129 (3.63e-05) | 5250236 (3.09e-04) | | 95. | Tspan11 | P < 5.775e-07 | 6950030 | 6950030 | 4799240 (5.77e-07) | | 96. | Tubb6 | P < 5.788e-07 | 6861707 | 6861707 | 4451890 (5.79e-07) | | 97. | Eln | P < 5.839e-07 | 6942380 | 6942380 | 4476878 (5.84e-07) | 4879529 (1.41e-06) | 4317297 (4.76e-04) | 4868330 (8.59e-04) | | 98. | B4galnt1 | P < 5.882e-07 | 6771546 | 6771546 | 5116127 (5.88e-07) | 5218551 (6.61e-05) | | 99. | Egfl6 | P < 5.968e-07 | 7020772 | 7020772 | 5321591 (5.97e-07) | 5195723 (1.47e-04) | | 100. | 9130213B05Rik | P < 5.982e-07 | 6932409 | 6932409 | 5342254 (5.98e-07) | | 101. | Psph | P < 6.014e-07 | 6942176 | 6942176 | 4313882 (6.01e-07) | 4512523 (6.73e-05) | 5152936 (2.40e-04) | | 102. | Zmiz1 | P < 6.344e-07 | 6817611 | 6817611 | 4754697 (6.34e-07) | | 103. | Pde4b | P < 6.794e-07 | 6915856 | 6915856 | 5167761 (6.79e-07) | 5210459 (8.81e-04) | | 104. | Traf1 | P < 7.142e-07 | 6886021 | 6886021 | 5154081 (7.14e-07) | | 105. | Bfar | P < 7.424e-07 | 6839528 | 6839528 | 5106297 (7.42e-07) | 5094213 (2.36e-04) | 4998507 (5.83e-04) | | 106. | Prdm16 | P < 7.513e-07 | 6927215 | 6927215 | 5156595 (7.51e-07) | 5285507 (2.71e-06) | 4437142 (3.97e-06) | 4328872 (2.11e-05) | 4678301 (4.60e-05) | 4465962 (4.99e-05) | 5322450 (2.52e-04) | 5184734 (4.27e-04) | 5062233 (6.87e-04) | 4795169 (1.02e-03) | | 107. | Acox1 | P < 7.539e-07 | 6792500 | 6792500 | 5518088 (7.54e-07) | | 108. | Eng | P < 7.583e-07 | 6876212 | 6876212 | 5490829 (7.58e-07) | 4515601 (2.23e-04) | | 109. | Barx1 | P < 7.875e-07 | 6806896 | 6806896 | 5083612 (7.88e-07) | | 110. | Sez6l2 | P < 8.273e-07 | 6964259 | 6964259 | 5433388 (8.27e-07) | 5079106 (5.54e-06) | 4996602 (2.02e-05) | 5088489 (6.56e-04) | | 111. | Araf | P < 8.457e-07 | 7010347 | 7010347 | 4454488 (8.46e-07) | 4379119 (8.81e-05) | 5054582 (4.22e-04) | 4337862 (5.54e-04) | | 112. | Rtn1 | P < 8.502e-07 | 6801636 | 6801636 | 4410939 (8.50e-07) | 5465505 (3.03e-06) | 5287394 (5.56e-06) | 5602313 (1.02e-05) | 5116236 (1.84e-05) | 5409037 (6.49e-05) | 5466222 (2.02e-04) | 5507542 (3.09e-04) | 4941697 (9.00e-04) | | 113. | Cct3 | P < 8.827e-07 | 6899104 | 6899104 | 4360558 (8.83e-07) | 5265866 (7.59e-04) | 5093273 (8.71e-04) | | 114. | Rufy3 | P < 8.905e-07 | 6932224 | 6932224 | 5572595 (8.90e-07) | 4509832 (3.71e-06) | 4328953 (7.14e-05) | 4428552 (9.30e-05) | 4321833 (9.70e-04) | | 115. | Obfc2a | P < 9.423e-07 | 6758588 | 6758588 | 4849050 (9.42e-07) | | 116. | Ddr2 | P < 9.506e-07 | 6764010 | 6764010 | 5400025 (9.51e-07) | 4899984 (3.16e-06) | | 117. | Rsl1d1 | P < 9.554e-07 | 6843951 | 6843951 | 5605874 (9.55e-07) | 5403149 (4.04e-04) | | 118. | Crhr1 | P < 9.568e-07 | 6784494 | 6784494 | 4771248 (9.57e-07) | | 119. | Timm17a | P < 9.591e-07 | 6762420 | 6762420 | 5519235 (9.59e-07) | | 120. | Syt4 | P < 9.927e-07 | 6864327 | 6864327 | 5283900 (9.93e-07) | | 121. | Il1r1 | P < 9.939e-07 | 6748884 | 6748884 | 4983516 (9.94e-07) | | 122. | Ncoa6 | P < 1.007e-06 | 6892371 | 6892371 | 5602099 (1.01e-06) | | 123. | Gldc | P < 1.015e-06 | 6872584 | 6872584 | 4580378 (1.01e-06) | 4402569 (1.55e-05) | 5365060 (4.07e-05) | 5341634 (3.15e-04) | | 124. | Col9a2 | P < 1.085e-06 | 6916849 | 6916849 | 5252322 (1.09e-06) | 4686510 (1.25e-05) | 5146010 (3.70e-05) | 5558680 (2.09e-04) | 5299615 (4.63e-04) | 5480610 (4.68e-04) | 4860272 (7.32e-04) | | 125. | Hapln1 | P < 1.086e-06 | 6808781 | 6808781 | 4657997 (1.09e-06) | | 126. | Lima1 | P < 1.183e-06 | 6838492 | 6838492 | 4969821 (1.18e-06) | 4531012 (2.82e-04) | | 127. | Hmga1 | P < 1.204e-06 | 6849507 | 6849507 | 5190724 (1.20e-06) | 4531186 (1.33e-06) | 4919920 (5.49e-06) | 4495516 (6.24e-06) | | 128. | Tcf7l2 | P < 1.217e-06 | 6870580 | 6870580 | 5230966 (1.22e-06) | 4416679 (6.62e-05) | 4411707 (4.40e-04) | 4698378 (6.76e-04) | 4964545 (8.76e-04) | | 129. | Naaa | P < 1.223e-06 | 6939985 | 6939985 | 4966609 (1.22e-06) | 4901182 (1.05e-03) | | 130. | Dagla | P < 1.244e-06 | 6871479 | 6871479 | 5288700 (1.24e-06) | 5479387 (1.26e-04) | 5193131 (6.14e-04) | | 131. | Emp1 | P < 1.245e-06 | 6950413 | 6950413 | 5146397 (1.24e-06) | | 132. | Myrip | P < 1.246e-06 | 6992942 | 6992942 | 5143642 (1.25e-06) | | 133. | Prtg | P < 1.303e-06 | 6990418 | 6990418 | 4781769 (1.30e-06) | 4887312 (2.03e-06) | 5536839 (4.10e-06) | 5440514 (7.86e-05) | 4554117 (1.21e-04) | 4594525 (1.77e-04) | 5272511 (2.99e-04) | 4918603 (9.00e-04) | | 134. | Csda | P < 1.319e-06 | 6957465 | 6957465 | 5168159 (1.32e-06) | 5434022 (6.01e-06) | 5600174 (7.62e-04) | | 135. | Cntn2 | P < 1.331e-06 | 6762234 | 6762234 | 4927176 (1.33e-06) | 4467314 (1.00e-04) | 4373946 (1.32e-04) | 5272324 (3.28e-04) | 5216422 (5.03e-04) | | 136. | Bai1 | P < 1.386e-06 | 6831511 | 6831511 | 5134283 (1.39e-06) | 5084995 (2.44e-04) | | 137. | Prkcz | P < 1.471e-06 | 6927248 | 6927248 | 5165980 (1.47e-06) | | 138. | Nedd9 | P < 1.475e-06 | 6812652 | 6812652 | 5303171 (1.47e-06) | 5175857 (8.41e-06) | | 139. | Nsdhl | P < 1.542e-06 | 7011907 | 7011907 | 5111725 (1.54e-06) | | 140. | Mki67ip | P < 1.566e-06 | 6752409 | 6752409 | 4448813 (1.57e-06) | 5206529 (2.56e-04) | | 141. | Prkcd | P < 1.626e-06 | 6823666 | 6823666 | 4864481 (1.63e-06) | | 142. | Leprel2 | P < 1.633e-06 | 6957138 | 6957138 | 5044420 (1.63e-06) | | 143. | Sox17 | P < 1.664e-06 | 6756599 | 6756599 | 5160098 (1.66e-06) | | 144. | Prmt5 | P < 1.671e-06 | 6824738 | 6824738 | 4849476 (1.67e-06) | | 145. | Rusc1 | P < 1.750e-06 | 6911212 | 6911212 | 4987651 (1.75e-06) | 4597177 (9.59e-05) | 4576448 (1.13e-04) | 4381935 (3.44e-04) | | 146. | Ogdh | P < 1.762e-06 | 6778560 | 6778560 | 5378617 (1.76e-06) | 5006114 (1.02e-05) | 4876532 (1.79e-04) | | 147. | Hand1 | P < 1.843e-06 | 6788536 | 6788536 | 5556710 (1.84e-06) | | 148. | Ihh | P < 1.874e-06 | 6759769 | 6759769 | 4802251 (1.87e-06) | 5368311 (1.49e-05) | | 149. | Npal3 | P < 1.891e-06 | 6926084 | 6926084 | 5326531 (1.89e-06) | | 150. | Cited2 | P < 1.936e-06 | 6766240 | 6766240 | 4827529 (1.94e-06) | | 151. | Wasf2 | P < 1.939e-06 | 6917557 | 6917557 | 5358739 (1.94e-06) | | 152. | 5830434P21Rik | P < 1.967e-06 | 6876173 | 6876173 | 4748742 (1.97e-06) | 5132409 (6.68e-05) | 4482427 (6.68e-04) | | 153. | Mbtd1 | P < 1.967e-06 | 6783626 | 6783626 | 5271006 (1.97e-06) | | 154. | 2610110G12Rik | P < 1.980e-06 | 6855144 | 6855144 | 4869479 (1.98e-06) | 4857648 (2.20e-04) | | 155. | Aes | P < 2.014e-06 | 6769304 | 6769304 | 4506595 (2.01e-06) | | 156. | Rab6 | P < 2.035e-06 | 6962950 | 6962950 | 5590127 (2.03e-06) | 5021115 (1.35e-04) | | 157. | Lass1 | P < 2.040e-06 | 6976991 | 6976991 | 4912580 (2.04e-06) | 4904577 (4.10e-06) | 5259039 (4.20e-06) | 4865445 (1.87e-04) | | 158. | Fn1 | P < 2.064e-06 | 6759621 | 6759621 | 5415737 (2.06e-06) | 4401495 (3.19e-06) | 4612563 (7.97e-06) | 4630802 (1.09e-05) | 5300313 (3.85e-05) | 4630426 (4.81e-05) | 5039118 (5.85e-05) | 5130413 (8.07e-05) | 4930621 (2.27e-04) | 5089929 (5.42e-04) | 5201971 (6.06e-04) | 4369368 (8.39e-04) | | 159. | Dst | P < 2.065e-06 | 6748525 | 6748525 | 4776151 (2.07e-06) | 4969885 (3.09e-05) | 5456344 (1.26e-04) | 4370202 (2.16e-04) | 5028582 (5.55e-04) | 4516010 (7.56e-04) | | 160. | Eef1a2 | P < 2.072e-06 | 6894268 | 6894268 | 5195888 (2.07e-06) | 5284153 (1.07e-05) | | 161. | Add1 | P < 2.146e-06 | 6929850 | 6929850 | 4470167 (2.15e-06) | | 162. | Ebf2 | P < 2.245e-06 | 6819948 | 6819948 | 4906861 (2.24e-06) | 4407034 (2.98e-05) | | 163. | Card14 | P < 2.245e-06 | 6785368 | 6785368 | 4787714 (2.24e-06) | | 164. | Nup160 | P < 2.354e-06 | 6878978 | 6878978 | 4536693 (2.35e-06) | 4395023 (2.22e-05) | | 165. | Ccdc21 | P < 2.379e-06 | 6925988 | 6925988 | 4495096 (2.38e-06) | | 166. | Mbnl2 | P < 2.450e-06 | 6822167 | 6822167 | 4849027 (2.45e-06) | | 167. | 0610010K14Rik | P < 2.451e-06 | 6789401 | 6789401 | 4690576 (2.45e-06) | | 168. | Fert2 | P < 2.460e-06 | 6851605 | 6851605 | 5548831 (2.46e-06) | | 169. | Prdm8 | P < 2.475e-06 | 6932704 | 6932704 | 5359732 (2.48e-06) | 5219864 (1.19e-05) | 5040322 (1.62e-04) | 4663415 (4.32e-04) | | 170. | Col17a1 | P < 2.516e-06 | 6873516 | 6873516 | 5275604 (2.52e-06) | 5426164 (2.21e-04) | 4956080 (6.36e-04) | | 171. | Atp1a2 | P < 2.554e-06 | 6764138 | 6764138 | 5196656 (2.55e-06) | 5548740 (7.79e-04) | | 172. | Plekha7 | P < 2.561e-06 | 6970744 | 6970744 | 4693359 (2.56e-06) | 4532640 (5.93e-05) | 4889753 (1.03e-04) | 4967061 (4.28e-04) | | 173. | Gnl3 | P < 2.613e-06 | 6823696 | 6823696 | 4578030 (2.61e-06) | | 174. | Atp1b1 | P < 2.661e-06 | 6763706 | 6763706 | 5190275 (2.66e-06) | | 175. | Sdk1 | P < 2.668e-06 | 6935312 | 6935312 | 4351922 (2.67e-06) | 5445101 (2.94e-04) | 4356517 (4.31e-04) | 5534437 (6.30e-04) | | 176. | Dnmt3b | P < 2.727e-06 | 6882397 | 6882397 | 5074130 (2.73e-06) | 5500912 (7.86e-04) | | 177. | Gemin5 | P < 2.755e-06 | 6788563 | 6788563 | 4422855 (2.75e-06) | | 178. | Prepl | P < 2.790e-06 | 6857834 | 6857834 | 4601289 (2.79e-06) | | 179. | Vav2 | P < 2.900e-06 | 6885530 | 6885530 | 4562685 (2.90e-06) | | 180. | Ltbp4 | P < 2.934e-06 | 6966039 | 6966039 | 5005630 (2.93e-06) | 5483408 (6.67e-06) | 5009636 (8.39e-04) | 5427101 (9.62e-04) | | 181. | Punc | P < 3.030e-06 | 6989914 | 6989914 | 5565647 (3.03e-06) | | 182. | Pnkd | P < 3.042e-06 | 6750532 | 6750532 | 4534764 (3.04e-06) | 4899967 (3.08e-05) | | 183. | Sgsm2 | P < 3.044e-06 | 6789693 | 6789693 | 4414752 (3.04e-06) | | 184. | Paqr4 | P < 3.147e-06 | 6854270 | 6854270 | 5460402 (3.15e-06) | | 185. | Rgs3 | P < 3.153e-06 | 6913985 | 6913985 | 5579440 (3.15e-06) | 5389311 (5.29e-05) | 4576439 (1.72e-04) | 4397974 (2.41e-04) | | 186. | NA | P < 3.184e-06 | 6946309 | 6946309 | 5485019 (3.18e-06) | | 187. | Psd2 | P < 3.228e-06 | 6860085 | 6860085 | 5184256 (3.23e-06) | 4741356 (3.15e-04) | | 188. | Cse1l | P < 3.228e-06 | 6883267 | 6883267 | 4884402 (3.23e-06) | | 189. | Rqcd1 | P < 3.272e-06 | 6750552 | 6750552 | 5377957 (3.27e-06) | | 190. | Slc39a5 | P < 3.426e-06 | 6778027 | 6778027 | 4668854 (3.43e-06) | 4642282 (1.45e-04) | 4718502 (2.52e-04) | | 191. | Pou6f1 | P < 3.442e-06 | 6838565 | 6838565 | 5318237 (3.44e-06) | 4517778 (3.47e-04) | 4316558 (3.71e-04) | 4952315 (5.73e-04) | | 192. | Slc4a3 | P < 3.498e-06 | 6750643 | 6750643 | 4745973 (3.50e-06) | 4736608 (1.91e-04) | | 193. | Sec14l2 | P < 3.523e-06 | 6785642 | 6785642 | 5573224 (3.52e-06) | 4716011 (9.92e-04) | | 194. | B4galnt3 | P < 3.629e-06 | 6956937 | 6956937 | 5082360 (3.63e-06) | 4914044 (1.08e-04) | 5030028 (1.68e-04) | | 195. | Celsr3 | P < 3.629e-06 | 6992373 | 6992373 | 5317025 (3.63e-06) | 4471143 (4.09e-05) | 5357945 (1.55e-04) | 4438920 (3.38e-04) | 5501605 (7.29e-04) | | 196. | Zdhhc1 | P < 3.685e-06 | 6984973 | 6984973 | 4700180 (3.68e-06) | | 197. | Tgm1 | P < 3.701e-06 | 6824838 | 6824838 | 4372380 (3.70e-06) | 4316762 (1.37e-04) | | 198. | Kcnq1 | P < 3.710e-06 | 6965319 | 6965319 | 5119439 (3.71e-06) | | 199. | Ctsh | P < 3.751e-06 | 6991264 | 6991264 | 4504264 (3.75e-06) | | 200. | Lrrn2 | P < 3.760e-06 | 6753157 | 6753157 | 4699169 (3.76e-06) | | 201. | Dsg2 | P < 3.793e-06 | 6859285 | 6859285 | 5075759 (3.79e-06) | | 202. | Ppan | P < 3.921e-06 | 6987343 | 6987343 | 4742906 (3.92e-06) | 5519165 (6.06e-05) | | 203. | Pkd1 | P < 3.928e-06 | 6849303 | 6849303 | 5336590 (3.93e-06) | 5503290 (9.67e-04) | | 204. | Dnm1 | P < 3.951e-06 | 6885871 | 6885871 | 4495299 (3.95e-06) | 4566222 (6.77e-06) | 5340607 (1.28e-05) | 5372697 (5.64e-05) | 5255766 (1.20e-04) | 4951987 (1.53e-04) | 4534177 (5.40e-04) | | 205. | Grwd1 | P < 3.998e-06 | 6967013 | 6967013 | 4954349 (4.00e-06) | | 206. | Pps | P < 4.017e-06 | 6782456 | 6782456 | 4540870 (4.02e-06) | | 207. | Txnl4a | P < 4.123e-06 | 6862459 | 6862459 | 5251037 (4.12e-06) | 4907963 (1.38e-05) | 5593095 (2.72e-04) | 4792056 (8.54e-04) | | 208. | Kcnq2 | P < 4.165e-06 | 6894265 | 6894265 | 4335279 (4.17e-06) | 5458937 (7.03e-06) | 4378601 (1.34e-04) | 4516277 (7.38e-04) | 4847293 (9.84e-04) | | 209. | Frk | P < 4.238e-06 | 6767094 | 6767094 | 5320479 (4.24e-06) | | 210. | Elavl3 | P < 4.254e-06 | 6993854 | 6993854 | 4423114 (4.25e-06) | 4769771 (6.60e-04) | | 211. | Asb7 | P < 4.329e-06 | 6967983 | 6967983 | 4785389 (4.33e-06) | 5296262 (4.86e-04) | | 212. | Stard7 | P < 4.373e-06 | 6880983 | 6880983 | 4437538 (4.37e-06) | | 213. | Erc1 | P < 4.412e-06 | 6956926 | 6956926 | 4875636 (4.41e-06) | 4994273 (2.72e-04) | 4413220 (5.79e-04) | | 214. | Eif2c3 | P < 4.416e-06 | 6925362 | 6925362 | 4967248 (4.42e-06) | | 215. | Usp10 | P < 4.435e-06 | 6979519 | 6979519 | 5591048 (4.44e-06) | | 216. | Kif13a | P < 4.486e-06 | 6812973 | 6812973 | 4855971 (4.49e-06) | | 217. | Atp2a1 | P < 4.517e-06 | 6971266 | 6971266 | 5153306 (4.52e-06) | 4518488 (7.56e-06) | 4343734 (1.85e-04) | 5202573 (6.10e-04) | | 218. | Lrp12 | P < 4.601e-06 | 6835222 | 6835222 | 5434631 (4.60e-06) | | 219. | Parp6 | P < 4.676e-06 | 6989556 | 6989556 | 4849641 (4.68e-06) | | 220. | Itga7 | P < 4.737e-06 | 6771718 | 6771718 | 4739437 (4.74e-06) | 4991477 (6.06e-04) | | 221. | Ggt5 | P < 4.840e-06 | 6768868 | 6768868 | 5362569 (4.84e-06) | 5226978 (1.02e-03) | | 222. | Atcay | P < 4.918e-06 | 6775441 | 6775441 | 4442706 (4.92e-06) | 5256296 (7.79e-05) | | 223. | Mpp6 | P < 4.975e-06 | 6946138 | 6946138 | 5534639 (4.97e-06) | | 224. | Itgb4 | P < 4.976e-06 | 6785183 | 6785183 | 5250660 (4.98e-06) | 5010470 (7.67e-05) | 5437828 (1.89e-04) | | 225. | Cyp20a1 | P < 5.107e-06 | 6749813 | 6749813 | 5024472 (5.11e-06) | | 226. | Fads2 | P < 5.197e-06 | 6871471 | 6871471 | 4949457 (5.20e-06) | | 227. | A2m | P < 5.360e-06 | 6949613 | 6949613 | 5576705 (5.36e-06) | 5560893 (1.18e-05) | 5535304 (1.29e-05) | 5248102 (2.71e-04) | | 228. | Slc16a1 | P < 5.482e-06 | 6900180 | 6900180 | 5480205 (5.48e-06) | 4558373 (1.89e-04) | | 229. | Ganab | P < 5.573e-06 | 6867976 | 6867976 | 5153997 (5.57e-06) | | 230. | Ndst1 | P < 5.587e-06 | 6865926 | 6865926 | 4367140 (5.59e-06) | 4351685 (1.85e-04) | | 231. | Klhl5 | P < 5.601e-06 | 6931229 | 6931229 | 5274433 (5.60e-06) | | 232. | Lctl | P < 5.662e-06 | 6989861 | 6989861 | 5391976 (5.66e-06) | | 233. | Crebzf | P < 5.773e-06 | 6962495 | 6962495 | 5274680 (5.77e-06) | 4753625 (1.01e-03) | | 234. | Ctage5 | P < 5.842e-06 | 6795455 | 6795455 | 5596927 (5.84e-06) | 4357794 (3.43e-04) | | 235. | Acly | P < 5.881e-06 | 6791428 | 6791428 | 4722306 (5.88e-06) | 5612014 (2.78e-04) | | 236. | Pml | P < 5.884e-06 | 6995964 | 6995964 | 5557104 (5.88e-06) | | 237. | Rbm9 | P < 5.957e-06 | 6836888 | 6836888 | 5448331 (5.96e-06) | 5479607 (7.92e-06) | | 238. | Sfxn3 | P < 6.010e-06 | 6869979 | 6869979 | 4880476 (6.01e-06) | | 239. | C3 | P < 6.041e-06 | 6856290 | 6856290 | 5501915 (6.04e-06) | 5155546 (1.83e-05) | 5155756 (2.21e-05) | 4863295 (1.10e-04) | 4400230 (9.23e-04) | | 240. | Slc44a2 | P < 6.063e-06 | 6987374 | 6987374 | 5412081 (6.06e-06) | 4770893 (2.61e-04) | | 241. | Nolc1 | P < 6.130e-06 | 6870027 | 6870027 | 4458112 (6.13e-06) | | 242. | Tcf7 | P < 6.159e-06 | 6788172 | 6788172 | 5389154 (6.16e-06) | 4340655 (1.91e-04) | | 243. | Clta | P < 6.200e-06 | 6913045 | 6913045 | 4399984 (6.20e-06) | 5176486 (2.45e-04) | | 244. | Nav1 | P < 6.205e-06 | 6762429 | 6762429 | 4540461 (6.20e-06) | 4438112 (3.42e-05) | 5407471 (5.84e-05) | 4409327 (3.37e-04) | 4536315 (3.92e-04) | 4309187 (6.80e-04) | | 245. | Vac14 | P < 6.265e-06 | 6979097 | 6979097 | 4884740 (6.27e-06) | | 246. | Stat6 | P < 6.348e-06 | 6771575 | 6771575 | 5123667 (6.35e-06) | 4984899 (8.38e-05) | 5439932 (1.11e-04) | 4603662 (8.93e-04) | | 247. | NA | P < 6.548e-06 | 6813887 | 6813887 | 5166752 (6.55e-06) | 5195124 (1.25e-05) | 5485654 (1.80e-04) | | 248. | Bcl7a | P < 6.550e-06 | 6934221 | 6934221 | 4937826 (6.55e-06) | 5487319 (3.40e-04) | 4671649 (8.78e-04) | | 249. | Pus1 | P < 6.581e-06 | 6941048 | 6941048 | 5415512 (6.58e-06) | 5073391 (8.64e-05) | | 250. | Micall2 | P < 6.589e-06 | 6942685 | 6942685 | 5000233 (6.59e-06) | 4760220 (5.46e-04) | | 251. | Gsc | P < 6.599e-06 | 6803297 | 6803297 | 5020429 (6.60e-06) | | 252. | 6720460F02Rik | P < 6.692e-06 | 6782214 | 6782214 | 4852844 (6.69e-06) | | 253. | Clasp2 | P < 6.924e-06 | 6992613 | 6992613 | 5207664 (6.92e-06) | | 254. | L1cam | P < 6.928e-06 | 7017600 | 7017600 | 4566587 (6.93e-06) | | 255. | Bai2 | P < 6.936e-06 | 6917353 | 6917353 | 5242049 (6.94e-06) | 4790810 (4.10e-04) | 5228173 (7.29e-04) | 4386402 (7.38e-04) | | 256. | Klf16 | P < 7.017e-06 | 6775379 | 6775379 | 4666517 (7.02e-06) | | 257. | Hsph1 | P < 7.206e-06 | 6943310 | 6943310 | 4728457 (7.21e-06) | 5262027 (4.50e-05) | 5247515 (7.94e-04) | | 258. | Hbb-y | P < 7.221e-06 | 6970009 | 6970009 | 4820949 (7.22e-06) | | 259. | Rgs12 | P < 7.266e-06 | 6929861 | 6929861 | 4493090 (7.27e-06) | 4766862 (5.44e-04) | | 260. | Dnajc2 | P < 7.346e-06 | 6936585 | 6936585 | 5441035 (7.35e-06) | 4862809 (6.20e-05) | 5152534 (1.17e-04) | | 261. | Phf17 | P < 7.409e-06 | 6897075 | 6897075 | 5196255 (7.41e-06) | | 262. | Smtnl2 | P < 7.618e-06 | 6789540 | 6789540 | 5169914 (7.62e-06) | | 263. | Rbl2 | P < 7.689e-06 | 6978156 | 6978156 | 4880869 (7.69e-06) | 4438562 (2.73e-05) | 4478027 (2.68e-04) | 4598697 (4.25e-04) | 4329649 (9.68e-04) | | 264. | Nkx3-2 | P < 7.804e-06 | 6937717 | 6937717 | 5485884 (7.80e-06) | | 265. | Schip1 | P < 7.929e-06 | 6898181 | 6898181 | 4306573 (7.93e-06) | 4783241 (1.19e-05) | 5612833 (3.21e-05) | | 266. | Wnk1 | P < 8.068e-06 | 6956932 | 6956932 | 5332694 (8.07e-06) | 4721679 (1.74e-05) | 5540775 (7.77e-04) | 4390830 (8.82e-04) | 5040197 (1.03e-03) | | 267. | Pdha1 | P < 8.272e-06 | 7020507 | 7020507 | 5608740 (8.27e-06) | 4394730 (1.02e-03) | | 268. | 2900083I11Rik | P < 8.474e-06 | 6934945 | 6934945 | 5113062 (8.47e-06) | | 269. | Folr1 | P < 8.515e-06 | 6969888 | 6969888 | 5181753 (8.52e-06) | 5386628 (2.12e-05) | 4320423 (3.95e-04) | | 270. | Stxbp1 | P < 8.553e-06 | 6885912 | 6885912 | 5419411 (8.55e-06) | 4897006 (4.88e-04) | | 271. | Gtf2a1 | P < 8.650e-06 | 6802744 | 6802744 | 4667020 (8.65e-06) | | 272. | Peli3 | P < 8.652e-06 | 6871060 | 6871060 | 5531304 (8.65e-06) | 4588055 (1.22e-04) | | 273. | Cxx1c | P < 8.794e-06 | 7011286 | 7011286 | 5296918 (8.79e-06) | | 274. | Bmp1 | P < 8.859e-06 | 6825704 | 6825704 | 4864133 (8.86e-06) | | 275. | Mycbp2 | P < 8.881e-06 | 6827135 | 6827135 | 4732293 (8.88e-06) | 5290332 (6.35e-04) | | 276. | St7 | P < 8.931e-06 | 6944385 | 6944385 | 4694297 (8.93e-06) | | 277. | Ncor2 | P < 8.986e-06 | 6941932 | 6941932 | 5408493 (8.99e-06) | | 278. | Depdc5 | P < 9.024e-06 | 6929762 | 6929762 | 5472984 (9.02e-06) | | 279. | Bsn | P < 9.074e-06 | 6998669 | 6998669 | 5229171 (9.07e-06) | | 280. | Ston2 | P < 9.155e-06 | 6802745 | 6802745 | 5247674 (9.16e-06) | 5363413 (2.21e-05) | 4416018 (9.27e-04) | | 281. | Apoa1 | P < 9.227e-06 | 6988769 | 6988769 | 5297394 (9.23e-06) | 5415898 (3.01e-05) | | 282. | Lmx1a | P < 9.259e-06 | 6754909 | 6754909 | 4819294 (9.26e-06) | 5473653 (1.43e-04) | 5497022 (9.85e-04) | | 283. | D9Ertd280e | P < 9.363e-06 | 6998213 | 6998213 | 4593401 (9.36e-06) | 5212758 (5.21e-04) | 5201710 (1.00e-03) | | 284. | 3110043J09Rik | P < 9.373e-06 | 6832324 | 6832324 | 4736502 (9.37e-06) | | 285. | Tnn | P < 9.428e-06 | 6763489 | 6763489 | 5112737 (9.43e-06) | | 286. | Foxp1 | P < 9.487e-06 | 6955879 | 6955879 | 4651360 (9.49e-06) | | 287. | Lrrfip2 | P < 9.515e-06 | 6992487 | 6992487 | 5254560 (9.51e-06) | 5296181 (6.24e-04) | | 288. | Ptgis | P < 9.893e-06 | 6893139 | 6893139 | 4323134 (9.89e-06) | | 289. | Ipo5 | P < 1.002e-05 | 6822191 | 6822191 | 5199327 (1.00e-05) | 4950103 (5.83e-05) | 4929884 (1.34e-04) | 5128581 (2.14e-04) | 4681618 (2.65e-04) | 5312209 (6.63e-04) | | 290. | Mmp9 | P < 1.008e-05 | 6883125 | 6883125 | 4306434 (1.01e-05) | | 291. | St3gal3 | P < 1.011e-05 | 6924877 | 6924877 | 4325730 (1.01e-05) | | 292. | Runx1t1 | P < 1.020e-05 | 6911833 | 6911833 | 5170627 (1.02e-05) | | 293. | Acin1 | P < 1.031e-05 | 6824755 | 6824755 | 4837295 (1.03e-05) | | 294. | Shisa4 | P < 1.043e-05 | 6762423 | 6762423 | 5030295 (1.04e-05) | | 295. | Ubox5 | P < 1.069e-05 | 6890918 | 6890918 | 4532968 (1.07e-05) | 4848344 (2.24e-05) | 5091555 (3.24e-04) | 4316927 (7.43e-04) | | 296. | NA | P < 1.070e-05 | 6958927 | 6958927 | 4436845 (1.07e-05) | | 297. | Aars | P < 1.075e-05 | 6979123 | 6979123 | 4380610 (1.07e-05) | | 298. | Zfp207 | P < 1.084e-05 | 6782818 | 6782818 | 5177372 (1.08e-05) | | 299. | Dysf | P < 1.085e-05 | 6947475 | 6947475 | 4541939 (1.09e-05) | 5178037 (5.07e-04) | | 300. | Gpx3 | P < 1.105e-05 | 6781090 | 6781090 | 5526930 (1.11e-05) | 4358746 (7.05e-05) | 5273560 (4.14e-04) | 5452784 (4.91e-04) | | 301. | Wfdc1 | P < 1.106e-05 | 6979508 | 6979508 | 4760295 (1.11e-05) | 4998842 (1.13e-04) | | 302. | Lcor | P < 1.120e-05 | 6869724 | 6869724 | 5174328 (1.12e-05) | 4707640 (6.61e-04) | | 303. | Rps13 | P < 1.144e-05 | 6985812 | 6985812 | 5063332 (1.14e-05) | | 304. | Zfhx3 | P < 1.152e-05 | 6979023 | 6979023 | 4749140 (1.15e-05) | 4726792 (1.42e-04) | 4893097 (7.27e-04) | 5267766 (7.69e-04) | | 305. | Stc2 | P < 1.162e-05 | 6787035 | 6787035 | 4731265 (1.16e-05) | | 306. | Abcg2 | P < 1.192e-05 | 6946554 | 6946554 | 4404627 (1.19e-05) | | 307. | Tbx4 | P < 1.194e-05 | 6783225 | 6783225 | 5125208 (1.19e-05) | 4373064 (8.04e-04) | | 308. | Kcnh2 | P < 1.197e-05 | 6936719 | 6936719 | 4613081 (1.20e-05) | 5573651 (4.62e-04) | | 309. | Hoxa7 | P < 1.200e-05 | 6953611 | 6953611 | 5073656 (1.20e-05) | | 310. | NA | P < 1.201e-05 | 6925165 | 6925165 | 4736995 (1.20e-05) | 4711701 (3.79e-05) | 5396726 (9.60e-05) | | 311. | Runx1 | P < 1.224e-05 | 6847972 | 6847972 | 4800865 (1.22e-05) | | 312. | Mylk | P < 1.229e-05 | 6840694 | 6840694 | 5496512 (1.23e-05) | | 313. | Pcsk9 | P < 1.232e-05 | 6924233 | 6924233 | 5426886 (1.23e-05) | | 314. | Vim | P < 1.240e-05 | 6875132 | 6875132 | 5100548 (1.24e-05) | 4912347 (6.88e-05) | 5579311 (1.72e-04) | | 315. | Tbl3 | P < 1.243e-05 | 6854386 | 6854386 | 5421522 (1.24e-05) | | 316. | NA | P < 1.249e-05 | 6960255 | 6960255 | 4643866 (1.25e-05) | 5325761 (8.55e-04) | | 317. | Zfp64 | P < 1.257e-05 | 6893303 | 6893303 | 5154866 (1.26e-05) | 5396161 (8.53e-04) | | 318. | Mbtps2 | P < 1.271e-05 | 7020412 | 7020412 | 4388470 (1.27e-05) | 5530952 (5.18e-04) | | 319. | Gba | P < 1.284e-05 | 6899213 | 6899213 | 5525829 (1.28e-05) | | 320. | Cct4 | P < 1.291e-05 | 6779359 | 6779359 | 4686197 (1.29e-05) | | 321. | Astn2 | P < 1.301e-05 | 6922382 | 6922382 | 5185623 (1.30e-05) | 5272445 (2.19e-05) | 5077001 (5.84e-05) | 4330515 (1.56e-04) | 5483006 (1.75e-04) | 4626327 (2.46e-04) | | 322. | NA | P < 1.301e-05 | 6760364 | 6760364 | 5008431 (1.30e-05) | | 323. | Sall2 | P < 1.310e-05 | 6824633 | 6824633 | 5474245 (1.31e-05) | 5528329 (8.17e-04) | | 324. | Stip1 | P < 1.316e-05 | 6871277 | 6871277 | 4366443 (1.32e-05) | | 325. | Adc | P < 1.324e-05 | 6925533 | 6925533 | 5078632 (1.32e-05) | 4872883 (1.16e-04) | 4861995 (2.33e-04) | 4344673 (4.05e-04) | 4620902 (6.16e-04) | | 326. | Lbp | P < 1.340e-05 | 6882730 | 6882730 | 4772863 (1.34e-05) | 5500123 (4.22e-04) | | 327. | Galntl4 | P < 1.344e-05 | 6970484 | 6970484 | 5268759 (1.34e-05) | 4474123 (9.69e-05) | | 328. | Ptprg | P < 1.344e-05 | 6816951 | 6816951 | 4785843 (1.34e-05) | | 329. | 4930402H24Rik | P < 1.346e-05 | 6890935 | 6890935 | 4760035 (1.35e-05) | 5019238 (1.07e-04) | 5010716 (1.58e-04) | 5246517 (6.96e-04) | 4587411 (1.03e-03) | | 330. | Fkbp11 | P < 1.382e-05 | 6838394 | 6838394 | 4452435 (1.38e-05) | 5305430 (8.90e-04) | | 331. | Apoe | P < 1.403e-05 | 6973587 | 6973587 | 5293248 (1.40e-05) | | 332. | Cpne1 | P < 1.405e-05 | 6892417 | 6892417 | 4853705 (1.40e-05) | 4448243 (2.74e-05) | 4875009 (3.79e-04) | | 333. | Mrps18b | P < 1.420e-05 | 6855145 | 6855145 | 4345350 (1.42e-05) | | 334. | Caskin2 | P < 1.423e-05 | 6792476 | 6792476 | 5602591 (1.42e-05) | | 335. | 9030409G11Rik | P < 1.465e-05 | 6926505 | 6926505 | 4413652 (1.46e-05) | 5312709 (1.45e-04) | 5551598 (2.56e-04) | 4715988 (8.59e-04) | | 336. | Gng2 | P < 1.469e-05 | 6823007 | 6823007 | 4377922 (1.47e-05) | | 337. | Klc1 | P < 1.490e-05 | 6798232 | 6798232 | 5157194 (1.49e-05) | 4307886 (6.01e-05) | 5444153 (1.33e-04) | | 338. | Net1 | P < 1.496e-05 | 6810688 | 6810688 | 4551581 (1.50e-05) | 4682494 (1.66e-04) | 5286893 (2.19e-04) | | 339. | Rnft2 | P < 1.498e-05 | 6941474 | 6941474 | 4996187 (1.50e-05) | 5436517 (8.47e-05) | 4966742 (7.73e-04) | | 340. | Tmem98 | P < 1.498e-05 | 6782859 | 6782859 | 5132625 (1.50e-05) | 4941577 (1.03e-03) | | 341. | Mta1 | P < 1.499e-05 | 6798312 | 6798312 | 5388994 (1.50e-05) | 4629085 (2.31e-05) | 5130374 (4.43e-04) | | 342. | Serinc2 | P < 1.519e-05 | 6925654 | 6925654 | 4727040 (1.52e-05) | | 343. | Kank4 | P < 1.529e-05 | 6923676 | 6923676 | 4861239 (1.53e-05) | | 344. | 0610010O12Rik | P < 1.533e-05 | 6860111 | 6860111 | 4395883 (1.53e-05) | 5494004 (8.05e-05) | | 345. | Fbxl12 | P < 1.548e-05 | 6993708 | 6993708 | 5289342 (1.55e-05) | | 346. | Senp7 | P < 1.557e-05 | 6841701 | 6841701 | 5213116 (1.56e-05) | 4737944 (5.41e-05) | | 347. | Slain2 | P < 1.560e-05 | 6931602 | 6931602 | 5551727 (1.56e-05) | | 348. | Car11 | P < 1.586e-05 | 6960403 | 6960403 | 4328729 (1.59e-05) | | 349. | Atp7b | P < 1.610e-05 | 6980938 | 6980938 | 4880762 (1.61e-05) | 4799548 (3.81e-05) | | 350. | Ssrp1 | P < 1.616e-05 | 6878730 | 6878730 | 5145850 (1.62e-05) | 4744127 (2.53e-04) | 4973521 (7.81e-04) | | 351. | Epas1 | P < 1.633e-05 | 6852836 | 6852836 | 5591003 (1.63e-05) | | 352. | Cct8 | P < 1.665e-05 | 6847632 | 6847632 | 5192289 (1.67e-05) | 5243102 (1.80e-04) | | 353. | Ubtf | P < 1.686e-05 | 6791558 | 6791558 | 5336515 (1.69e-05) | | 354. | Pdzk1ip1 | P < 1.702e-05 | 6916488 | 6916488 | 4980766 (1.70e-05) | 5614857 (1.47e-04) | 4454181 (9.43e-04) | | 355. | D19Bwg1357e | P < 1.719e-05 | 6872426 | 6872426 | 5363546 (1.72e-05) | | 356. | Krt5 | P < 1.735e-05 | 6838655 | 6838655 | 4828720 (1.74e-05) | 5055646 (4.42e-05) | 5322084 (6.11e-05) | | 357. | Ap3b2 | P < 1.736e-05 | 6968829 | 6968829 | 5062979 (1.74e-05) | 5326650 (1.79e-04) | 4390288 (2.49e-04) | 4547300 (2.85e-04) | 4696780 (3.09e-04) | | 358. | Ppa1 | P < 1.748e-05 | 6768232 | 6768232 | 4765367 (1.75e-05) | 4933702 (4.28e-04) | | 359. | Cdx1 | P < 1.757e-05 | 6865958 | 6865958 | 4597313 (1.76e-05) | 5472848 (2.56e-04) | 5300764 (2.76e-04) | | 360. | Mtap4 | P < 1.759e-05 | 6992415 | 6992415 | 4961304 (1.76e-05) | | 361. | Pde7b | P < 1.781e-05 | 6772550 | 6772550 | 5614919 (1.78e-05) | | 362. | 2210009G21Rik | P < 1.803e-05 | 6881499 | 6881499 | 5411298 (1.80e-05) | | 363. | Pvrl4 | P < 1.807e-05 | 6755175 | 6755175 | 5199847 (1.81e-05) | | 364. | Zfp318 | P < 1.843e-05 | 6850695 | 6850695 | 4529921 (1.84e-05) | | 365. | Bnc2 | P < 1.845e-05 | 6922966 | 6922966 | 5464083 (1.84e-05) | | 366. | Vill | P < 1.856e-05 | 6992866 | 6992866 | 4556520 (1.86e-05) | 5372479 (7.47e-05) | 5560213 (1.24e-04) | 5121684 (4.57e-04) | 4487428 (4.60e-04) | 4470753 (4.84e-04) | | 367. | Rbbp7 | P < 1.857e-05 | 7014929 | 7014929 | 4463405 (1.86e-05) | | 368. | Gramd1b | P < 1.879e-05 | 6994830 | 6994830 | 4902135 (1.88e-05) | 4772530 (8.92e-05) | | 369. | Ubp1 | P < 1.892e-05 | 6992614 | 6992614 | 4781972 (1.89e-05) | | 370. | Fgfr1 | P < 1.899e-05 | 6974743 | 6974743 | 5202052 (1.90e-05) | 5228863 (4.37e-04) | | 371. | Tcf12 | P < 1.907e-05 | 6996704 | 6996704 | 5070994 (1.91e-05) | 5447032 (6.11e-05) | 4873710 (6.94e-05) | 4842285 (7.81e-04) | | 372. | Rab27a | P < 1.919e-05 | 6990435 | 6990435 | 5049448 (1.92e-05) | | 373. | Slc39a4 | P < 1.931e-05 | 6836839 | 6836839 | 5263046 (1.93e-05) | 5479205 (5.94e-04) | | 374. | Traip | P < 1.963e-05 | 6992278 | 6992278 | 4991846 (1.96e-05) | | 375. | Ptgs1 | P < 1.971e-05 | 6876430 | 6876430 | 4752803 (1.97e-05) | | 376. | Rbbp5 | P < 1.977e-05 | 6753135 | 6753135 | 4443392 (1.98e-05) | 4472207 (2.08e-04) | | 377. | Nope | P < 1.990e-05 | 6989912 | 6989912 | 4414726 (1.99e-05) | 4568029 (3.25e-05) | 4368756 (1.26e-04) | 4608044 (3.26e-04) | 4445844 (4.00e-04) | | 378. | Myb | P < 1.999e-05 | 6772594 | 6772594 | 4574272 (2.00e-05) | 5561239 (1.11e-04) | | 379. | Kpnb1 | P < 2.003e-05 | 6791151 | 6791151 | 4889042 (2.00e-05) | 4839227 (2.77e-04) | 4419275 (5.45e-04) | | 380. | Nudt18 | P < 2.008e-05 | 6820088 | 6820088 | 4388560 (2.01e-05) | | 381. | Pus7 | P < 2.016e-05 | 6936690 | 6936690 | 4473565 (2.02e-05) | | 382. | Bgn | P < 2.017e-05 | 7011936 | 7011936 | 4881856 (2.02e-05) | 4957928 (8.30e-04) | | 383. | Mmd2 | P < 2.017e-05 | 6942841 | 6942841 | 5391807 (2.02e-05) | 5270991 (7.47e-05) | | 384. | 1810054D07Rik | P < 2.021e-05 | 6922901 | 6922901 | 4447288 (2.02e-05) | 4853470 (7.92e-04) | | 385. | Cdk2 | P < 2.025e-05 | 6778055 | 6778055 | 4983803 (2.03e-05) | | 386. | NA | P < 2.028e-05 | 6854893 | 6854893 | 5039351 (2.03e-05) | | 387. | Ptprd | P < 2.036e-05 | 6922672 | 6922672 | 4400455 (2.04e-05) | 5073327 (8.41e-05) | 5433919 (6.15e-04) | | 388. | Ass1 | P < 2.083e-05 | 6768261 | 6768261 | 5020033 (2.08e-05) | 4648666 (1.49e-04) | | 389. | Adcy4 | P < 2.100e-05 | 6824844 | 6824844 | 5216249 (2.10e-05) | | 390. | Htt | P < 2.111e-05 | 6929856 | 6929856 | 5322754 (2.11e-05) | 4849466 (7.92e-04) | | 391. | 2210010B09Rik | P < 2.128e-05 | 6987324 | 6987324 | 4616294 (2.13e-05) | | 392. | Mamdc2 | P < 2.142e-05 | 6872204 | 6872204 | 5372453 (2.14e-05) | 5330665 (2.95e-05) | | 393. | Pnn | P < 2.159e-05 | 6795451 | 6795451 | 5127908 (2.16e-05) | 4712324 (5.22e-05) | 5285377 (3.22e-04) | | 394. | Col6a1 | P < 2.178e-05 | 6775185 | 6775185 | 4654309 (2.18e-05) | 4588012 (1.78e-04) | 5480557 (5.28e-04) | | 395. | Cdc25a | P < 2.178e-05 | 6992414 | 6992414 | 4549200 (2.18e-05) | | 396. | Tnk1 | P < 2.180e-05 | 6789355 | 6789355 | 4548254 (2.18e-05) | | 397. | Vps13a | P < 2.202e-05 | 6871837 | 6871837 | 5412307 (2.20e-05) | | 398. | Gstm4 | P < 2.210e-05 | 6908088 | 6908088 | 4784612 (2.21e-05) | | 399. | Slc7a2 | P < 2.241e-05 | 6975658 | 6975658 | 4535728 (2.24e-05) | 4733328 (3.14e-04) | | 400. | Tspan9 | P < 2.249e-05 | 6957316 | 6957316 | 4887299 (2.25e-05) | | 401. | Zhx2 | P < 2.285e-05 | 6830619 | 6830619 | 4602238 (2.28e-05) | | 402. | Itga11 | P < 2.292e-05 | 6989752 | 6989752 | 4504645 (2.29e-05) | 4918464 (1.04e-03) | | 403. | Xrcc6bp1 | P < 2.328e-05 | 6777879 | 6777879 | 4763867 (2.33e-05) | 4736772 (1.53e-04) | | 404. | Lpl | P < 2.347e-05 | 6976901 | 6976901 | 4827892 (2.35e-05) | | 405. | Lats2 | P < 2.355e-05 | 6824967 | 6824967 | 4356505 (2.35e-05) | | 406. | Fcer1g | P < 2.359e-05 | 6764049 | 6764049 | 4896021 (2.36e-05) | | 407. | Prdx5 | P < 2.359e-05 | 6871258 | 6871258 | 4806652 (2.36e-05) | | 408. | Epb4.1l2 | P < 2.359e-05 | 6766705 | 6766705 | 5458579 (2.36e-05) | 5133021 (3.04e-05) | | 409. | Zfp451 | P < 2.373e-05 | 6757745 | 6757745 | 5553334 (2.37e-05) | | 410. | Ccne1 | P < 2.385e-05 | 6966600 | 6966600 | 5292483 (2.39e-05) | 4971537 (2.43e-04) | | 411. | Cacna1b | P < 2.396e-05 | 6885345 | 6885345 | 4536683 (2.40e-05) | 4390271 (6.45e-05) | 4492089 (1.74e-04) | 5315158 (7.42e-04) | | 412. | Hexim1 | P < 2.473e-05 | 6784400 | 6784400 | 4437456 (2.47e-05) | | 413. | Tpm2 | P < 2.489e-05 | 6921161 | 6921161 | 4871513 (2.49e-05) | 5173306 (2.86e-05) | 5211654 (7.54e-05) | 5465692 (1.80e-04) | 5523996 (2.74e-04) | 4727405 (7.93e-04) | | 414. | Chst8 | P < 2.511e-05 | 6966453 | 6966453 | 4542434 (2.51e-05) | | 415. | Eif3eip | P < 2.533e-05 | 6831867 | 6831867 | 5199031 (2.53e-05) | | 416. | Uhrf1 | P < 2.568e-05 | 6851232 | 6851232 | 5591543 (2.57e-05) | 5136315 (1.79e-04) | | 417. | Rspo1 | P < 2.581e-05 | 6917065 | 6917065 | 5516382 (2.58e-05) | | 418. | Col4a4 | P < 2.597e-05 | 6760194 | 6760194 | 5603952 (2.60e-05) | | 419. | Fanci | P < 2.610e-05 | 6961917 | 6961917 | 5566639 (2.61e-05) | | 420. | Cyfip2 | P < 2.613e-05 | 6787743 | 6787743 | 4663602 (2.61e-05) | | 421. | BC031353 | P < 2.641e-05 | 6990525 | 6990525 | 4883995 (2.64e-05) | 4622970 (1.41e-04) | 5342851 (1.03e-03) | | 422. | Tmem126a | P < 2.653e-05 | 6969289 | 6969289 | 5184471 (2.65e-05) | | 423. | 2410008K03Rik | P < 2.658e-05 | 6778383 | 6778383 | 4480798 (2.66e-05) | | 424. | Dhx30 | P < 2.670e-05 | 6998829 | 6998829 | 5428526 (2.67e-05) | 4505698 (2.43e-04) | 5114636 (8.11e-04) | | 425. | Rev3l | P < 2.681e-05 | 6767292 | 6767292 | 5033240 (2.68e-05) | 5439217 (2.72e-04) | | 426. | Tshz2 | P < 2.714e-05 | 6883487 | 6883487 | 4688745 (2.71e-05) | 5162543 (4.81e-05) | | 427. | 6030446N20Rik | P < 2.768e-05 | 6863417 | 6863417 | 4862392 (2.77e-05) | | 428. | Cbx7 | P < 2.777e-05 | 6837143 | 6837143 | 5390149 (2.78e-05) | | 429. | Insm1 | P < 2.780e-05 | 6881903 | 6881903 | 4644937 (2.78e-05) | 4610261 (7.73e-04) | | 430. | Fst | P < 2.781e-05 | 6816226 | 6816226 | 5589635 (2.78e-05) | | 431. | Ncapd3 | P < 2.803e-05 | 6987641 | 6987641 | 4825375 (2.80e-05) | | 432. | Tspan5 | P < 2.824e-05 | 6901780 | 6901780 | 5142294 (2.82e-05) | | 433. | Skiv2l | P < 2.825e-05 | 6855060 | 6855060 | 4311068 (2.83e-05) | 4750628 (2.57e-04) | | 434. | Cast | P < 2.845e-05 | 6814407 | 6814407 | 5424210 (2.85e-05) | 4667776 (4.63e-05) | 4957673 (1.43e-04) | | 435. | Rabggtb | P < 2.854e-05 | 6910708 | 6910708 | 4535729 (2.85e-05) | 4806304 (5.45e-04) | | 436. | Mfge8 | P < 2.866e-05 | 6968683 | 6968683 | 4485965 (2.87e-05) | | 437. | Eif4e2 | P < 2.890e-05 | 6751332 | 6751332 | 4337460 (2.89e-05) | 5359860 (2.23e-04) | | 438. | Rcan1 | P < 2.891e-05 | 6847952 | 6847952 | 4567413 (2.89e-05) | | 439. | Spint1 | P < 2.903e-05 | 6880508 | 6880508 | 5611490 (2.90e-05) | 4762284 (3.88e-04) | | 440. | Obsl1 | P < 2.940e-05 | 6759817 | 6759817 | 4488766 (2.94e-05) | 5477458 (1.59e-04) | | 441. | Cx3cl1 | P < 2.959e-05 | 6978335 | 6978335 | 4423543 (2.96e-05) | | 442. | Pnliprp2 | P < 2.967e-05 | 6870746 | 6870746 | 5454608 (2.97e-05) | | 443. | Col1a2 | P < 2.991e-05 | 6943818 | 6943818 | 4516711 (2.99e-05) | 5162119 (1.33e-04) | 5563977 (2.85e-04) | 5569365 (8.73e-04) | | 444. | Gpr85 | P < 3.008e-05 | 6951761 | 6951761 | 4607871 (3.01e-05) | | 445. | Tcfcp2 | P < 3.024e-05 | 6838564 | 6838564 | 5145007 (3.02e-05) | | 446. | P2rx6 | P < 3.081e-05 | 6839751 | 6839751 | 4560260 (3.08e-05) | | 447. | Ints1 | P < 3.134e-05 | 6942688 | 6942688 | 4398764 (3.13e-05) | 4308262 (9.49e-04) | | 448. | Xkr7 | P < 3.143e-05 | 6882346 | 6882346 | 4908568 (3.14e-05) | | 449. | Prss33 | P < 3.145e-05 | 6854286 | 6854286 | 4358861 (3.14e-05) | | 450. | Rasa4 | P < 3.163e-05 | 6934972 | 6934972 | 5215978 (3.16e-05) | 5215621 (5.70e-04) | | 451. | Adcy3 | P < 3.191e-05 | 6792970 | 6792970 | 4916012 (3.19e-05) | | 452. | Brca2 | P < 3.242e-05 | 6935756 | 6935756 | 4964734 (3.24e-05) | | 453. | Krt7 | P < 3.259e-05 | 6833326 | 6833326 | 5466939 (3.26e-05) | 5102615 (3.96e-04) | | 454. | Crebl1 | P < 3.261e-05 | 6850064 | 6850064 | 4367042 (3.26e-05) | | 455. | Gbe1 | P < 3.285e-05 | 6842273 | 6842273 | 4828701 (3.29e-05) | | 456. | Tnc | P < 3.324e-05 | 6922335 | 6922335 | 4949913 (3.32e-05) | 4625217 (6.55e-04) | | 457. | Dtx1 | P < 3.326e-05 | 6941645 | 6941645 | 5319911 (3.33e-05) | | 458. | Bop1 | P < 3.338e-05 | 6836827 | 6836827 | 5269799 (3.34e-05) | | 459. | Acaca | P < 3.341e-05 | 6783063 | 6783063 | 4459323 (3.34e-05) | 5375962 (3.24e-04) | 4342699 (3.84e-04) | 4796277 (6.64e-04) | 5318885 (6.74e-04) | | 460. | AI118078 | P < 3.358e-05 | 6989258 | 6989258 | 4899104 (3.36e-05) | | 461. | Igsf9 | P < 3.453e-05 | 6755241 | 6755241 | 5049138 (3.45e-05) | | 462. | Rtn2 | P < 3.469e-05 | 6959000 | 6959000 | 4842360 (3.47e-05) | | 463. | Jmjd2a | P < 3.492e-05 | 6924878 | 6924878 | 4905627 (3.49e-05) | | 464. | Zfp598 | P < 3.507e-05 | 6849310 | 6849310 | 5359063 (3.51e-05) | | 465. | 4732415M23Rik | P < 3.510e-05 | 6979687 | 6979687 | 4692124 (3.51e-05) | | 466. | Fbxw7 | P < 3.535e-05 | 6898873 | 6898873 | 4389430 (3.54e-05) | | 467. | Mpp3 | P < 3.541e-05 | 6791529 | 6791529 | 4686375 (3.54e-05) | 5209787 (5.91e-04) | | 468. | Pld2 | P < 3.548e-05 | 6782134 | 6782134 | 4387360 (3.55e-05) | | 469. | Fkbp4 | P < 3.584e-05 | 6957346 | 6957346 | 5291152 (3.58e-05) | | 470. | Slc2a1 | P < 3.601e-05 | 6916748 | 6916748 | 5265034 (3.60e-05) | 5202805 (8.61e-05) | | 471. | Epb4.1 | P < 3.614e-05 | 6925776 | 6925776 | 5012146 (3.61e-05) | | 472. | Tpcn1 | P < 3.617e-05 | 6941637 | 6941637 | 5209774 (3.62e-05) | | 473. | Brunol6 | P < 3.619e-05 | 6989554 | 6989554 | 4517898 (3.62e-05) | | 474. | Nap1l1 | P < 3.635e-05 | 6770718 | 6770718 | 4969108 (3.63e-05) | | 475. | Pum1 | P < 3.644e-05 | 6917383 | 6917383 | 5330957 (3.64e-05) | | 476. | Mt1 | P < 3.649e-05 | 6978291 | 6978291 | 5274067 (3.65e-05) | | 477. | Gramd1a | P < 3.655e-05 | 6966358 | 6966358 | 5324446 (3.65e-05) | | 478. | Osbpl5 | P < 3.666e-05 | 6972418 | 6972418 | 4497097 (3.67e-05) | 5516534 (6.20e-04) | | 479. | Cyp46a1 | P < 3.772e-05 | 6797893 | 6797893 | 5029918 (3.77e-05) | | 480. | Emb | P < 3.875e-05 | 6810548 | 6810548 | 4847114 (3.87e-05) | 5399643 (8.79e-05) | 4506277 (2.24e-04) | 5043061 (8.23e-04) | | 481. | Rab25 | P < 3.892e-05 | 6906762 | 6906762 | 4622435 (3.89e-05) | | 482. | Hkdc1 | P < 3.896e-05 | 6774392 | 6774392 | 5021728 (3.90e-05) | | 483. | Pygm | P < 3.926e-05 | 6867859 | 6867859 | 4878582 (3.93e-05) | 5526691 (1.25e-04) | 4374092 (6.41e-04) | | 484. | Mcf2l | P < 3.926e-05 | 6974114 | 6974114 | 4366071 (3.93e-05) | 4445937 (1.70e-04) | 5085001 (6.49e-04) | 5440970 (6.59e-04) | 5046049 (6.79e-04) | 4888968 (9.28e-04) | | 485. | Tmem160 | P < 3.928e-05 | 6958855 | 6958855 | 5024430 (3.93e-05) | | 486. | Loxl1 | P < 3.954e-05 | 6995968 | 6995968 | 5031445 (3.95e-05) | 5278980 (2.78e-04) | | 487. | Llgl2 | P < 3.971e-05 | 6785168 | 6785168 | 4843349 (3.97e-05) | 5254915 (7.93e-04) | | 488. | Tacc1 | P < 4.000e-05 | 6981125 | 6981125 | 4606375 (4.00e-05) | 5485150 (2.09e-04) | | 489. | Plcxd1 | P < 4.005e-05 | 6933390 | 6933390 | 4841765 (4.01e-05) | 4518622 (1.10e-04) | | 490. | Pphln1 | P < 4.010e-05 | 6832775 | 6832775 | 5218867 (4.01e-05) | | 491. | Ctsf | P < 4.013e-05 | 6867701 | 6867701 | 4471555 (4.01e-05) | 5547232 (4.05e-04) | | 492. | Phf8 | P < 4.039e-05 | 7014545 | 7014545 | 5029742 (4.04e-05) | 4648757 (5.55e-04) | | 493. | Axl | P < 4.054e-05 | 6965982 | 6965982 | 5092754 (4.05e-05) | | 494. | Aldh1l1 | P < 4.057e-05 | 6947936 | 6947936 | 5130028 (4.06e-05) | 4720313 (2.61e-04) | | 495. | Mcm6 | P < 4.103e-05 | 6762017 | 6762017 | 4633908 (4.10e-05) | | 496. | Ahi1 | P < 4.117e-05 | 6766409 | 6766409 | 5483040 (4.12e-05) | | 497. | Doc2b | P < 4.146e-05 | 6789785 | 6789785 | 5419182 (4.15e-05) | 5100527 (6.00e-05) | | 498. | Mutyh | P < 4.154e-05 | 6916597 | 6916597 | 4671077 (4.15e-05) | | 499. | NA | P < 4.194e-05 | 6909304 | 6909304 | 4385486 (4.19e-05) | | 500. | Cdca7l | P < 4.201e-05 | 6798429 | 6798429 | 4351573 (4.20e-05) | 5578956 (3.24e-04) | | 501. | Tbc1d9 | P < 4.214e-05 | 6977648 | 6977648 | 4358015 (4.21e-05) | 4893156 (7.49e-04) | | 502. | Rnaseh1 | P < 4.237e-05 | 6794059 | 6794059 | 5501742 (4.24e-05) | | 503. | 2610510H03Rik | P < 4.238e-05 | 6880337 | 6880337 | 5199137 (4.24e-05) | 5336647 (2.29e-04) | 4796324 (1.05e-03) | | 504. | Nanos3 | P < 4.253e-05 | 6983826 | 6983826 | 5455979 (4.25e-05) | | 505. | Six2 | P < 4.277e-05 | 6857876 | 6857876 | 5289790 (4.28e-05) | | 506. | Fhit | P < 4.293e-05 | 6822526 | 6822526 | 4476128 (4.29e-05) | | 507. | Smarca2 | P < 4.302e-05 | 6868884 | 6868884 | 5292535 (4.30e-05) | 5598890 (6.16e-04) | 4838652 (6.77e-04) | | 508. | 4632411B12Rik | P < 4.313e-05 | 6757906 | 6757906 | 4936215 (4.31e-05) | 5201641 (1.41e-04) | 4939712 (3.13e-04) | | 509. | Deaf1 | P < 4.348e-05 | 6972200 | 6972200 | 4930679 (4.35e-05) | 5277440 (1.21e-04) | | 510. | 8430427H17Rik | P < 4.351e-05 | 6892230 | 6892230 | 5315102 (4.35e-05) | | 511. | Tmem164 | P < 4.397e-05 | 7014222 | 7014222 | 4405376 (4.40e-05) | 5298352 (1.01e-03) | | 512. | Yy1 | P < 4.407e-05 | 6797950 | 6797950 | 5075758 (4.41e-05) | | 513. | Ap3m2 | P < 4.416e-05 | 6980968 | 6980968 | 5304598 (4.42e-05) | | 514. | Scnn1g | P < 4.440e-05 | 6964011 | 6964011 | 4960560 (4.44e-05) | | 515. | NA | P < 4.446e-05 | 6942636 | 6942636 | 4466033 (4.45e-05) | | 516. | Limch1 | P < 4.482e-05 | 6931362 | 6931362 | 5153639 (4.48e-05) | 5206727 (3.86e-04) | | 517. | Dixdc1 | P < 4.556e-05 | 6995521 | 6995521 | 4831249 (4.56e-05) | 4525137 (1.40e-04) | 4836296 (5.10e-04) | 5105910 (9.36e-04) | 5038252 (1.00e-03) | | 518. | Adamtsl5 | P < 4.558e-05 | 6775367 | 6775367 | 5153840 (4.56e-05) | 4808829 (3.33e-04) | 5416537 (5.52e-04) | 5358328 (8.07e-04) | | 519. | Topbp1 | P < 4.631e-05 | 6992022 | 6992022 | 5583668 (4.63e-05) | 5309399 (2.67e-04) | 4562668 (9.49e-04) | | 520. | Prkrir | P < 4.659e-05 | 6962829 | 6962829 | 5260221 (4.66e-05) | | 521. | Eml3 | P < 4.693e-05 | 6867980 | 6867980 | 5195056 (4.69e-05) | 4837990 (4.45e-04) | | 522. | Paqr8 | P < 4.765e-05 | 6747986 | 6747986 | 5473258 (4.77e-05) | | 523. | Zfp473 | P < 4.798e-05 | 6966895 | 6966895 | 4877677 (4.80e-05) | | 524. | Sdk2 | P < 4.835e-05 | 6792314 | 6792314 | 4308172 (4.84e-05) | 5566036 (1.53e-04) | 5236132 (2.57e-04) | 5508177 (2.82e-04) | 4634166 (3.31e-04) | 5330718 (9.04e-04) | | 525. | Anks1 | P < 4.917e-05 | 6849525 | 6849525 | 4936718 (4.92e-05) | 5089854 (4.50e-04) | 4510106 (5.50e-04) | | 526. | Tfip11 | P < 4.920e-05 | 6933513 | 6933513 | 4456436 (4.92e-05) | | 527. | Srrm1 | P < 4.923e-05 | 6926076 | 6926076 | 5152594 (4.92e-05) | | 528. | Mat2a | P < 4.959e-05 | 6954626 | 6954626 | 4658478 (4.96e-05) | 5247478 (9.99e-04) | 4875721 (1.02e-03) | | 529. | Megf10 | P < 5.076e-05 | 6861173 | 6861173 | 4914188 (5.08e-05) | 4377989 (5.87e-05) | 5492153 (2.87e-04) | 5191940 (5.59e-04) | | 530. | Itga6 | P < 5.108e-05 | 6878031 | 6878031 | 5594042 (5.11e-05) | | 531. | Csad | P < 5.125e-05 | 6838713 | 6838713 | 4436055 (5.12e-05) | | 532. | Postn | P < 5.128e-05 | 6897557 | 6897557 | 4516400 (5.13e-05) | | 533. | Plekha6 | P < 5.149e-05 | 6753185 | 6753185 | 4522075 (5.15e-05) | | 534. | NA | P < 5.169e-05 | 6993833 | 6993833 | 5099755 (5.17e-05) | | 535. | Slc16a9 | P < 5.172e-05 | 6768618 | 6768618 | 5036999 (5.17e-05) | 4694299 (3.64e-04) | 4572726 (7.53e-04) | | 536. | Nat13 | P < 5.174e-05 | 6841097 | 6841097 | 4996372 (5.17e-05) | | 537. | Tnpo3 | P < 5.206e-05 | 6952376 | 6952376 | 5093440 (5.21e-05) | 5245677 (6.89e-05) | | 538. | Abca7 | P < 5.217e-05 | 6769180 | 6769180 | 4651511 (5.22e-05) | 4873224 (2.50e-04) | | 539. | Cdca7 | P < 5.274e-05 | 6878053 | 6878053 | 4787242 (5.27e-05) | 4689681 (4.75e-04) | | 540. | Vti1a | P < 5.278e-05 | 6870569 | 6870569 | 5354444 (5.28e-05) | 5371106 (1.75e-04) | 5389162 (9.08e-04) | | 541. | Pla2g2e | P < 5.286e-05 | 6918021 | 6918021 | 4361949 (5.29e-05) | | 542. | Col16a1 | P < 5.333e-05 | 6917365 | 6917365 | 5425761 (5.33e-05) | 5361735 (1.94e-04) | | 543. | Cxxc1 | P < 5.341e-05 | 6862085 | 6862085 | 4373950 (5.34e-05) | | 544. | Prmt3 | P < 5.347e-05 | 6960622 | 6960622 | 5118280 (5.35e-05) | | 545. | Rnf150 | P < 5.349e-05 | 6977643 | 6977643 | 4377654 (5.35e-05) | | 546. | Hpx | P < 5.357e-05 | 6970137 | 6970137 | 4844263 (5.36e-05) | | 547. | Wdfy3 | P < 5.368e-05 | 6940431 | 6940431 | 4951750 (5.37e-05) | 4987347 (3.02e-04) | | 548. | Anln | P < 5.378e-05 | 6993890 | 6993890 | 4307203 (5.38e-05) | | 549. | Pcnt | P < 5.387e-05 | 6775175 | 6775175 | 5213994 (5.39e-05) | 4886040 (8.30e-05) | 5458348 (3.15e-04) | | 550. | Epc1 | P < 5.405e-05 | 6863167 | 6863167 | 4534905 (5.40e-05) | 5076281 (5.73e-05) | 4387113 (1.93e-04) | | 551. | Wdr55 | P < 5.406e-05 | 6860129 | 6860129 | 4925620 (5.41e-05) | | 552. | Stat5b | P < 5.408e-05 | 6791444 | 6791444 | 5503064 (5.41e-05) | | 553. | Tspan17 | P < 5.425e-05 | 6807192 | 6807192 | 4924429 (5.42e-05) | | 554. | NA | P < 5.433e-05 | 6959397 | 6959397 | 4734839 (5.43e-05) | 4861517 (6.14e-05) | | 555. | Slit1 | P < 5.456e-05 | 6873149 | 6873149 | 4662555 (5.46e-05) | | 556. | Churc1 | P < 5.499e-05 | 6796208 | 6796208 | 5389000 (5.50e-05) | | 557. | Armc8 | P < 5.567e-05 | 6998217 | 6998217 | 5465664 (5.57e-05) | 4852551 (4.16e-04) | | 558. | P140 | P < 5.635e-05 | 6791204 | 6791204 | 4482555 (5.63e-05) | 4458562 (3.17e-04) | | 559. | Fasn | P < 5.648e-05 | 6792822 | 6792822 | 4361144 (5.65e-05) | | 560. | Cirh1a | P < 5.680e-05 | 6978935 | 6978935 | 5278900 (5.68e-05) | | 561. | Psmc6 | P < 5.704e-05 | 6818556 | 6818556 | 4323592 (5.70e-05) | | 562. | Foxp4 | P < 5.717e-05 | 6855801 | 6855801 | 4450387 (5.72e-05) | | 563. | Ppm1h | P < 5.734e-05 | 6771334 | 6771334 | 5307370 (5.73e-05) | | 564. | Gatm | P < 5.734e-05 | 6890448 | 6890448 | 5144283 (5.73e-05) | 4920477 (2.23e-04) | | 565. | Tmeff2 | P < 5.740e-05 | 6749346 | 6749346 | 5197299 (5.74e-05) | | 566. | Th | P < 5.743e-05 | 6972328 | 6972328 | 5568498 (5.74e-05) | 5169032 (9.07e-04) | | 567. | Tpx2 | P < 5.791e-05 | 6882333 | 6882333 | 4661432 (5.79e-05) | | 568. | EG331392 | P < 5.806e-05 | 7010378 | 7010378 | 5275744 (5.81e-05) | | 569. | Pprc1 | P < 5.808e-05 | 6870025 | 6870025 | 4465082 (5.81e-05) | | 570. | Kif5a | P < 5.818e-05 | 6777921 | 6777921 | 5352277 (5.82e-05) | 4525678 (4.50e-04) | 4798271 (8.84e-04) | | 571. | Hmgb3 | P < 5.905e-05 | 7011852 | 7011852 | 4977834 (5.90e-05) | | 572. | Ptpn5 | P < 5.909e-05 | 6967109 | 6967109 | 5602963 (5.91e-05) | 4663152 (1.69e-04) | | 573. | Grm4 | P < 5.960e-05 | 6854537 | 6854537 | 4686125 (5.96e-05) | | 574. | Tm4sf1 | P < 6.030e-05 | 6905289 | 6905289 | 5011566 (6.03e-05) | | 575. | Nol5a | P < 6.041e-05 | 6881172 | 6881172 | 4621202 (6.04e-05) | | 576. | Igf2bp1 | P < 6.042e-05 | 6791072 | 6791072 | 4963532 (6.04e-05) | 5573008 (1.88e-04) | | 577. | Lama1 | P < 6.059e-05 | 6851848 | 6851848 | 4954857 (6.06e-05) | 4649537 (7.69e-05) | 5386162 (1.15e-04) | | 578. | Lgals9 | P < 6.071e-05 | 6790012 | 6790012 | 5592906 (6.07e-05) | | 579. | Tyro3 | P < 6.107e-05 | 6880544 | 6880544 | 4781926 (6.11e-05) | | 580. | Tcfl5 | P < 6.123e-05 | 6894205 | 6894205 | 5043430 (6.12e-05) | | 581. | Bdnf | P < 6.169e-05 | 6879925 | 6879925 | 4680085 (6.17e-05) | | 582. | Rgs16 | P < 6.258e-05 | 6754138 | 6754138 | 4569622 (6.26e-05) | | 583. | Mbnl3 | P < 6.313e-05 | 7016804 | 7016804 | 4652219 (6.31e-05) | | 584. | Prickle3 | P < 6.320e-05 | 7009775 | 7009775 | 4844203 (6.32e-05) | | 585. | Rin2 | P < 6.367e-05 | 6881895 | 6881895 | 5159219 (6.37e-05) | 4355196 (1.59e-04) | 5049980 (3.87e-04) | 5357843 (4.84e-04) | 5235457 (7.04e-04) | | 586. | Drg2 | P < 6.387e-05 | 6781441 | 6781441 | 5306164 (6.39e-05) | 5067274 (6.34e-04) | | 587. | 4921513D23Rik | P < 6.438e-05 | 6844110 | 6844110 | 5002152 (6.44e-05) | | 588. | Ing4 | P < 6.518e-05 | 6949841 | 6949841 | 5377668 (6.52e-05) | | 589. | Pigo | P < 6.565e-05 | 6921129 | 6921129 | 5402029 (6.57e-05) | | 590. | Atp1b3 | P < 6.579e-05 | 6998049 | 6998049 | 4317790 (6.58e-05) | 5467000 (1.25e-04) | | 591. | Col5a1 | P < 6.592e-05 | 6875920 | 6875920 | 4719912 (6.59e-05) | | 592. | Fbxo41 | P < 6.614e-05 | 6955151 | 6955151 | 4805916 (6.61e-05) | | 593. | Plk3 | P < 6.625e-05 | 6924832 | 6924832 | 4491761 (6.62e-05) | | 594. | NA | P < 6.648e-05 | 6780868 | 6780868 | 5205554 (6.65e-05) | | 595. | Tsc2 | P < 6.707e-05 | 6854378 | 6854378 | 5286759 (6.71e-05) | | 596. | Nol9 | P < 6.761e-05 | 6918998 | 6918998 | 4902464 (6.76e-05) | | 597. | NA | P < 6.798e-05 | 6974081 | 6974081 | 4802588 (6.80e-05) | 5614273 (2.17e-04) | 4540800 (2.29e-04) | | 598. | Sept9 | P < 6.851e-05 | 6785261 | 6785261 | 4348550 (6.85e-05) | 4980666 (2.04e-04) | 4838634 (7.59e-04) | | 599. | Magee1 | P < 6.859e-05 | 7013139 | 7013139 | 4403964 (6.86e-05) | | 600. | Arhgef12 | P < 6.903e-05 | 6994954 | 6994954 | 5209805 (6.90e-05) | | 601. | Btnl2 | P < 6.945e-05 | 6850025 | 6850025 | 4488430 (6.94e-05) | 5167724 (2.21e-04) | | 602. | Pak4 | P < 6.956e-05 | 6966158 | 6966158 | 4618887 (6.96e-05) | 4532746 (8.57e-04) | | 603. | Elk4 | P < 6.990e-05 | 6753091 | 6753091 | 4780886 (6.99e-05) | | 604. | Spag9 | P < 7.053e-05 | 6783640 | 6783640 | 5492942 (7.05e-05) | 4739903 (2.35e-04) | 4752785 (3.51e-04) | | 605. | Fes | P < 7.064e-05 | 6968780 | 6968780 | 4672629 (7.06e-05) | | 606. | Rgnef | P < 7.068e-05 | 6815382 | 6815382 | 4491161 (7.07e-05) | | 607. | Tnfrsf14 | P < 7.092e-05 | 6927231 | 6927231 | 4428133 (7.09e-05) | | 608. | Mapk14 | P < 7.113e-05 | 6849567 | 6849567 | 5587922 (7.11e-05) | | 609. | Hoxd10 | P < 7.126e-05 | 6878265 | 6878265 | 4860187 (7.13e-05) | | 610. | Uchl5 | P < 7.177e-05 | 6753710 | 6753710 | 4616533 (7.18e-05) | | 611. | Adsl | P < 7.198e-05 | 6832079 | 6832079 | 5197576 (7.20e-05) | 4389710 (1.31e-04) | | 612. | Wdsof1 | P < 7.199e-05 | 6829964 | 6829964 | 5409015 (7.20e-05) | | 613. | Tmem134 | P < 7.202e-05 | 6867642 | 6867642 | 4952678 (7.20e-05) | | 614. | Cldn23 | P < 7.206e-05 | 6981713 | 6981713 | 4992092 (7.21e-05) | | 615. | Galnt1 | P < 7.265e-05 | 6859428 | 6859428 | 5451726 (7.26e-05) | | 616. | Pitx2 | P < 7.317e-05 | 6901280 | 6901280 | 5370473 (7.32e-05) | | 617. | Aplp1 | P < 7.334e-05 | 6966284 | 6966284 | 4724088 (7.33e-05) | | 618. | Zfp740 | P < 7.354e-05 | 6833398 | 6833398 | 4552183 (7.35e-05) | | 619. | Dlg3 | P < 7.417e-05 | 7012845 | 7012845 | 5344442 (7.42e-05) | 4791891 (2.51e-04) | 5313869 (3.13e-04) | | 620. | Akp5 | P < 7.425e-05 | 6760410 | 6760410 | 5200061 (7.43e-05) | | 621. | Gpr56 | P < 7.458e-05 | 6978354 | 6978354 | 4433528 (7.46e-05) | | 622. | Dnmt1 | P < 7.469e-05 | 6993722 | 6993722 | 5439138 (7.47e-05) | 4475647 (2.16e-04) | 5340921 (6.97e-04) | | 623. | Col6a2 | P < 7.522e-05 | 6775182 | 6775182 | 4345821 (7.52e-05) | 4875668 (1.73e-04) | 5129268 (2.65e-04) | | 624. | Sfrs1 | P < 7.527e-05 | 6783359 | 6783359 | 5409565 (7.53e-05) | 4782503 (9.90e-04) | | 625. | Lrrfip1 | P < 7.572e-05 | 6751525 | 6751525 | 5460141 (7.57e-05) | 4648001 (4.52e-04) | | 626. | Kcns3 | P < 7.620e-05 | 6798933 | 6798933 | 5093139 (7.62e-05) | | 627. | Tmem16b | P < 7.644e-05 | 6949885 | 6949885 | 4501732 (7.64e-05) | | 628. | Pou2f1 | P < 7.693e-05 | 6763810 | 6763810 | 4425985 (7.69e-05) | | 629. | 1110031B06Rik | P < 7.693e-05 | 6788672 | 6788672 | 4659289 (7.69e-05) | | 630. | Spsb1 | P < 7.772e-05 | 6926987 | 6926987 | 4327614 (7.77e-05) | | 631. | Abcc1 | P < 7.813e-05 | 6839607 | 6839607 | 5116413 (7.81e-05) | | 632. | Arrdc2 | P < 7.842e-05 | 6983230 | 6983230 | 5296233 (7.84e-05) | | 633. | Bhmt | P < 7.865e-05 | 6815126 | 6815126 | 4943552 (7.87e-05) | 5160984 (5.85e-04) | | 634. | Smc3 | P < 7.924e-05 | 6870470 | 6870470 | 4850497 (7.92e-05) | 5350594 (4.43e-04) | | 635. | Espn | P < 7.942e-05 | 6927124 | 6927124 | 4445772 (7.94e-05) | 4607289 (4.38e-04) | | 636. | Nr1d1 | P < 7.996e-05 | 6791282 | 6791282 | 4833184 (8.00e-05) | | 637. | Ccdc5 | P < 8.008e-05 | 6866839 | 6866839 | 4380939 (8.01e-05) | | 638. | 2810004N23Rik | P < 8.013e-05 | 6986086 | 6986086 | 4795889 (8.01e-05) | | 639. | Kif2a | P < 8.069e-05 | 6815797 | 6815797 | 5345537 (8.07e-05) | | 640. | Btrc | P < 8.071e-05 | 6869997 | 6869997 | 4810903 (8.07e-05) | | 641. | Npcd | P < 8.143e-05 | 6837131 | 6837131 | 4725846 (8.14e-05) | | 642. | Cacnb1 | P < 8.185e-05 | 6791233 | 6791233 | 4623022 (8.18e-05) | 4391320 (7.97e-04) | | 643. | Stx3 | P < 8.195e-05 | 6871580 | 6871580 | 4770528 (8.19e-05) | 5387146 (1.06e-04) | 4549423 (4.58e-04) | | 644. | Npc1 | P < 8.198e-05 | 6863434 | 6863434 | 4543690 (8.20e-05) | | 645. | Map3k4 | P < 8.230e-05 | 6853762 | 6853762 | 4554055 (8.23e-05) | 5445594 (8.91e-04) | | 646. | Tfdp2 | P < 8.230e-05 | 6991563 | 6991563 | 4319499 (8.23e-05) | 4463373 (3.99e-04) | | 647. | Abi2 | P < 8.233e-05 | 6749817 | 6749817 | 4987109 (8.23e-05) | | 648. | Ube4b | P < 8.286e-05 | 6926941 | 6926941 | 4933933 (8.29e-05) | | 649. | Unc50 | P < 8.308e-05 | 6748701 | 6748701 | 5469484 (8.31e-05) | 4931580 (1.61e-04) | | 650. | Adamts18 | P < 8.345e-05 | 6985481 | 6985481 | 4703571 (8.35e-05) | | 651. | Tm9sf2 | P < 8.350e-05 | 6822296 | 6822296 | 4417365 (8.35e-05) | | 652. | Prkar1a | P < 8.384e-05 | 6784810 | 6784810 | 4940692 (8.38e-05) | | 653. | AW146020 | P < 8.400e-05 | 6947321 | 6947321 | 5278798 (8.40e-05) | | 654. | Fryl | P < 8.431e-05 | 6939093 | 6939093 | 4917938 (8.43e-05) | | 655. | NA | P < 8.446e-05 | 6901732 | 6901732 | 4951266 (8.45e-05) | | 656. | Syne2 | P < 8.461e-05 | 6796158 | 6796158 | 5442201 (8.46e-05) | 5595407 (5.86e-04) | | 657. | Adam10 | P < 8.478e-05 | 6990295 | 6990295 | 4815182 (8.48e-05) | | 658. | Mak16 | P < 8.628e-05 | 6981497 | 6981497 | 4770807 (8.63e-05) | | 659. | Abr | P < 8.638e-05 | 6789817 | 6789817 | 4835966 (8.64e-05) | | 660. | Pld3 | P < 8.668e-05 | 6966058 | 6966058 | 5356153 (8.67e-05) | | 661. | Rundc3a | P < 8.705e-05 | 6784343 | 6784343 | 5582973 (8.71e-05) | 4318143 (2.43e-04) | 4452094 (6.06e-04) | | 662. | Dph3 | P < 8.776e-05 | 6823768 | 6823768 | 4361759 (8.78e-05) | | 663. | Atmin | P < 8.778e-05 | 6979424 | 6979424 | 5448666 (8.78e-05) | | 664. | Rhcg | P < 8.785e-05 | 6968712 | 6968712 | 5200402 (8.78e-05) | | 665. | Impdh1 | P < 8.793e-05 | 6952340 | 6952340 | 5535176 (8.79e-05) | | 666. | Acot10 | P < 8.802e-05 | 7014702 | 7014702 | 5358410 (8.80e-05) | | 667. | Ube2c | P < 8.848e-05 | 6883114 | 6883114 | 4753408 (8.85e-05) | | 668. | Elf1 | P < 8.859e-05 | 6820573 | 6820573 | 4330524 (8.86e-05) | | 669. | Psmd11 | P < 8.907e-05 | 6782828 | 6782828 | 5229005 (8.91e-05) | | 670. | Hoxa11 | P < 8.908e-05 | 6953617 | 6953617 | 5063800 (8.91e-05) | | 671. | Fdft1 | P < 9.027e-05 | 6825302 | 6825302 | 4362412 (9.03e-05) | | 672. | Eif3s10 | P < 9.047e-05 | 6874212 | 6874212 | 4498262 (9.05e-05) | 5061670 (3.87e-04) | 4440377 (4.25e-04) | 4415631 (8.72e-04) | | 673. | Hmha1 | P < 9.106e-05 | 6769181 | 6769181 | 4391418 (9.11e-05) | | 674. | Crmp1 | P < 9.147e-05 | 6930022 | 6930022 | 4920111 (9.15e-05) | 4504241 (9.33e-05) | | 675. | Unc5a | P < 9.179e-05 | 6807201 | 6807201 | 5197700 (9.18e-05) | | 676. | Tmem163 | P < 9.190e-05 | 6761964 | 6761964 | 5493998 (9.19e-05) | | 677. | Chac1 | P < 9.202e-05 | 6880516 | 6880516 | 4607647 (9.20e-05) | | 678. | Apc2 | P < 9.210e-05 | 6769209 | 6769209 | 4558140 (9.21e-05) | 5210903 (2.84e-04) | | 679. | Herc2 | P < 9.276e-05 | 6960846 | 6960846 | 4354869 (9.28e-05) | 4415806 (1.60e-04) | | 680. | Zmat3 | P < 9.279e-05 | 6904047 | 6904047 | 5116816 (9.28e-05) | | 681. | 1110017D15Rik | P < 9.287e-05 | 6921036 | 6921036 | 5431483 (9.29e-05) | | 682. | Mapre2 | P < 9.296e-05 | 6859411 | 6859411 | 5333666 (9.30e-05) | | 683. | Syt16 | P < 9.303e-05 | 6796053 | 6796053 | 5365478 (9.30e-05) | | 684. | Mast1 | P < 9.345e-05 | 6983890 | 6983890 | 5083139 (9.35e-05) | | 685. | Bag2 | P < 9.373e-05 | 6757744 | 6757744 | 5575652 (9.37e-05) | | 686. | Evx1 | P < 9.404e-05 | 6946250 | 6946250 | 5304924 (9.40e-05) | 4460814 (1.83e-04) | | 687. | Mtap7d1 | P < 9.433e-05 | 6925350 | 6925350 | 4858528 (9.43e-05) | | 688. | NA | P < 9.439e-05 | 6849968 | 6849968 | 4448393 (9.44e-05) | | 689. | Ctsb | P < 9.499e-05 | 6819694 | 6819694 | 5371824 (9.50e-05) | 5126942 (2.44e-04) | | 690. | Adamts2 | P < 9.564e-05 | 6780811 | 6780811 | 4326100 (9.56e-05) | | 691. | Dnajc10 | P < 9.603e-05 | 6878511 | 6878511 | 5147279 (9.60e-05) | 4350896 (4.44e-04) | 4628452 (1.04e-03) | | 692. | Mrpl11 | P < 9.684e-05 | 6867706 | 6867706 | 5588542 (9.68e-05) | | 693. | Madd | P < 9.689e-05 | 6888751 | 6888751 | 4675436 (9.69e-05) | | 694. | Ppef2 | P < 9.711e-05 | 6939979 | 6939979 | 4607644 (9.71e-05) | 4428124 (1.48e-04) | 5080847 (7.49e-04) | | 695. | Hpn | P < 9.741e-05 | 6966354 | 6966354 | 5549604 (9.74e-05) | | 696. | Pip5k3 | P < 9.745e-05 | 6750086 | 6750086 | 4619781 (9.75e-05) | | 697. | Zfp84 | P < 9.759e-05 | 6959534 | 6959534 | 5433703 (9.76e-05) | | 698. | Zfpm2 | P < 9.874e-05 | 6830026 | 6830026 | 4402927 (9.87e-05) | | 699. | Kif20b | P < 9.900e-05 | 6869370 | 6869370 | 5069113 (9.90e-05) | 4921228 (3.26e-04) | | 700. | Sigirr | P < 9.943e-05 | 6972174 | 6972174 | 4895373 (9.94e-05) | | 701. | Rae1 | P < 9.973e-05 | 6883641 | 6883641 | 5492248 (9.97e-05) | | 702. | Thbs1 | P < 1.006e-04 | 6880393 | 6880393 | 4426664 (1.01e-04) | 4636327 (7.15e-04) | 4497837 (7.94e-04) | | 703. | Wiz | P < 1.011e-04 | 6854874 | 6854874 | 4819604 (1.01e-04) | | 704. | Tspan14 | P < 1.012e-04 | 6824142 | 6824142 | 4347437 (1.01e-04) | | 705. | Ankrd24 | P < 1.013e-04 | 6769309 | 6769309 | 5073049 (1.01e-04) | | 706. | Xpo5 | P < 1.019e-04 | 6850683 | 6850683 | 4936087 (1.02e-04) | | 707. | Gns | P < 1.020e-04 | 6771224 | 6771224 | 4378374 (1.02e-04) | | 708. | Arrb1 | P < 1.027e-04 | 6962880 | 6962880 | 5034986 (1.03e-04) | | 709. | 2610204M08Rik | P < 1.032e-04 | 6798269 | 6798269 | 4484417 (1.03e-04) | | 710. | Aebp1 | P < 1.032e-04 | 6778521 | 6778521 | 5030863 (1.03e-04) | 5323024 (1.33e-04) | | 711. | Mad2l1 | P < 1.034e-04 | 6946778 | 6946778 | 4414369 (1.03e-04) | 5241538 (2.93e-04) | | 712. | Trim29 | P < 1.038e-04 | 6988571 | 6988571 | 4729346 (1.04e-04) | 5257748 (5.03e-04) | | 713. | Impact | P < 1.038e-04 | 6858936 | 6858936 | 5244384 (1.04e-04) | | 714. | Trib2 | P < 1.038e-04 | 6799173 | 6799173 | 5060331 (1.04e-04) | | 715. | Prep | P < 1.041e-04 | 6767610 | 6767610 | 4334813 (1.04e-04) | | 716. | Slc9a9 | P < 1.045e-04 | 6991461 | 6991461 | 5262693 (1.05e-04) | | 717. | Nus1 | P < 1.049e-04 | 6767845 | 6767845 | 5015679 (1.05e-04) | | 718. | Nkx1-2 | P < 1.051e-04 | 6971687 | 6971687 | 4881706 (1.05e-04) | | 719. | 2610507B11Rik | P < 1.052e-04 | 6782685 | 6782685 | 4446657 (1.05e-04) | | 720. | Rgmb | P < 1.057e-04 | 6853960 | 6853960 | 4662548 (1.06e-04) | | 721. | Narf | P < 1.057e-04 | 6785538 | 6785538 | 5508407 (1.06e-04) | | 722. | 4932441K18Rik | P < 1.064e-04 | 7020636 | 7020636 | 4564945 (1.06e-04) | 5375608 (5.54e-04) | | 723. | Mrpl15 | P < 1.064e-04 | 6756625 | 6756625 | 5566174 (1.06e-04) | | 724. | Phf12 | P < 1.070e-04 | 6782643 | 6782643 | 4848654 (1.07e-04) | | 725. | Tbc1d8 | P < 1.071e-04 | 6758027 | 6758027 | 5122778 (1.07e-04) | 4854358 (7.09e-04) | | 726. | 5430432M24Rik | P < 1.074e-04 | 6892104 | 6892104 | 4615423 (1.07e-04) | | 727. | Afg3l1 | P < 1.082e-04 | 6979709 | 6979709 | 4354173 (1.08e-04) | | 728. | Dido1 | P < 1.084e-04 | 6894212 | 6894212 | 4418325 (1.08e-04) | | 729. | Arrdc1 | P < 1.085e-04 | 6885351 | 6885351 | 4814206 (1.09e-04) | | 730. | Eps15l1 | P < 1.087e-04 | 6983325 | 6983325 | 4633242 (1.09e-04) | | 731. | Smarcad1 | P < 1.090e-04 | 6946729 | 6946729 | 5129899 (1.09e-04) | | 732. | Cpne2 | P < 1.091e-04 | 6978319 | 6978319 | 4830991 (1.09e-04) | | 733. | Aurka | P < 1.092e-04 | 6893486 | 6893486 | 5522781 (1.09e-04) | | 734. | Jag2 | P < 1.093e-04 | 6803917 | 6803917 | 4370082 (1.09e-04) | | 735. | Tfpi | P < 1.099e-04 | 6888299 | 6888299 | 4843014 (1.10e-04) | | 736. | St6galnac2 | P < 1.112e-04 | 6792544 | 6792544 | 5208322 (1.11e-04) | 5584478 (6.41e-04) | | 737. | Ttc24 | P < 1.125e-04 | 6906703 | 6906703 | 5521308 (1.13e-04) | | 738. | Nes | P < 1.130e-04 | 6899039 | 6899039 | 5005084 (1.13e-04) | | 739. | Vsig2 | P < 1.130e-04 | 6988222 | 6988222 | 4539870 (1.13e-04) | | 740. | Svop | P < 1.132e-04 | 6941189 | 6941189 | 5178777 (1.13e-04) | | 741. | Sult2b1 | P < 1.133e-04 | 6967006 | 6967006 | 4888844 (1.13e-04) | 5103410 (1.25e-04) | | 742. | Pde3a | P < 1.134e-04 | 6950718 | 6950718 | 4587715 (1.13e-04) | 4390466 (8.24e-04) | | 743. | Ptdss1 | P < 1.138e-04 | 6807790 | 6807790 | 5001468 (1.14e-04) | | 744. | 2010011I20Rik | P < 1.141e-04 | 6883598 | 6883598 | 4628927 (1.14e-04) | | 745. | Lpar1 | P < 1.146e-04 | 6922004 | 6922004 | 5549607 (1.15e-04) | | 746. | Ints12 | P < 1.148e-04 | 6901489 | 6901489 | 5272510 (1.15e-04) | | 747. | Gprasp1 | P < 1.151e-04 | 7013952 | 7013952 | 5082900 (1.15e-04) | 5487466 (5.11e-04) | 4979596 (5.58e-04) | 4783684 (9.64e-04) | | 748. | Sema3b | P < 1.156e-04 | 6998639 | 6998639 | 4734887 (1.16e-04) | 5527134 (6.98e-04) | | 749. | Acss1 | P < 1.160e-04 | 6892032 | 6892032 | 4821056 (1.16e-04) | | 750. | Morc4 | P < 1.163e-04 | 7019785 | 7019785 | 4599832 (1.16e-04) | | 751. | Ahdc1 | P < 1.167e-04 | 6917551 | 6917551 | 5360665 (1.17e-04) | | 752. | Ezh2 | P < 1.170e-04 | 6953331 | 6953331 | 4485025 (1.17e-04) | 4748591 (3.52e-04) | 4869780 (4.56e-04) | | 753. | Tmem150 | P < 1.172e-04 | 6946971 | 6946971 | 4373670 (1.17e-04) | | 754. | Narg1l | P < 1.175e-04 | 6826266 | 6826266 | 5549085 (1.17e-04) | | 755. | Stk24 | P < 1.180e-04 | 6827912 | 6827912 | 5558221 (1.18e-04) | | 756. | Stom | P < 1.181e-04 | 6886039 | 6886039 | 5474045 (1.18e-04) | 4480320 (1.78e-04) | | 757. | Ebna1bp2 | P < 1.185e-04 | 6916722 | 6916722 | 5414961 (1.18e-04) | | 758. | Phyhipl | P < 1.195e-04 | 6774875 | 6774875 | 5445347 (1.20e-04) | | 759. | Cdca4 | P < 1.198e-04 | 6803912 | 6803912 | 5578826 (1.20e-04) | | 760. | Abca4 | P < 1.209e-04 | 6900982 | 6900982 | 4848858 (1.21e-04) | 5338778 (8.86e-04) | | 761. | S100a1 | P < 1.211e-04 | 6906903 | 6906903 | 5201724 (1.21e-04) | 4970860 (8.14e-04) | | 762. | Paxip1 | P < 1.212e-04 | 6936855 | 6936855 | 5264853 (1.21e-04) | 5531322 (7.14e-04) | | 763. | Sltm | P < 1.214e-04 | 6990281 | 6990281 | 5094972 (1.21e-04) | | 764. | Rad52 | P < 1.221e-04 | 6949509 | 6949509 | 5611638 (1.22e-04) | | 765. | Nasp | P < 1.224e-04 | 6924801 | 6924801 | 5342773 (1.22e-04) | | 766. | BC029214 | P < 1.233e-04 | 6885412 | 6885412 | 4360854 (1.23e-04) | | 767. | Bin1 | P < 1.237e-04 | 6859814 | 6859814 | 5095505 (1.24e-04) | 5499601 (6.01e-04) | | 768. | R3hdm2 | P < 1.244e-04 | 6771568 | 6771568 | 5039407 (1.24e-04) | | 769. | Pard3 | P < 1.245e-04 | 6979993 | 6979993 | 5156154 (1.24e-04) | 4429520 (2.70e-04) | | 770. | Aacs | P < 1.255e-04 | 6934401 | 6934401 | 4827794 (1.25e-04) | | 771. | Tubgcp2 | P < 1.261e-04 | 6972110 | 6972110 | 4500607 (1.26e-04) | | 772. | Obfc2b | P < 1.265e-04 | 6778028 | 6778028 | 5428594 (1.27e-04) | | 773. | Cygb | P < 1.266e-04 | 6792539 | 6792539 | 4454612 (1.27e-04) | 5345264 (9.98e-04) | | 774. | Dgcr8 | P < 1.270e-04 | 6844329 | 6844329 | 4911736 (1.27e-04) | 4791338 (7.97e-04) | | 775. | Dnajc6 | P < 1.272e-04 | 6915844 | 6915844 | 5228910 (1.27e-04) | 5575875 (2.30e-04) | 4603342 (3.17e-04) | 5256610 (5.23e-04) | 5357236 (6.96e-04) | | 776. | Casp8ap2 | P < 1.272e-04 | 6912491 | 6912491 | 5585188 (1.27e-04) | | 777. | Atp9a | P < 1.273e-04 | 6893288 | 6893288 | 4708656 (1.27e-04) | 4996316 (3.85e-04) | | 778. | Mybph | P < 1.278e-04 | 6753250 | 6753250 | 4602202 (1.28e-04) | 4564122 (1.84e-04) | 4966286 (3.40e-04) | | 779. | NA | P < 1.283e-04 | 6913344 | 6913344 | 4551146 (1.28e-04) | | 780. | Idh1 | P < 1.294e-04 | 6759396 | 6759396 | 5077561 (1.29e-04) | | 781. | Gnas | P < 1.308e-04 | 6883737 | 6883737 | 5201088 (1.31e-04) | 5109379 (6.66e-04) | | 782. | Cherp | P < 1.310e-04 | 6983327 | 6983327 | 5530525 (1.31e-04) | | 783. | Tmem110 | P < 1.314e-04 | 6817952 | 6817952 | 4610418 (1.31e-04) | | 784. | Rabgap1l | P < 1.317e-04 | 6763495 | 6763495 | 5273014 (1.32e-04) | | 785. | Fchsd2 | P < 1.324e-04 | 6962999 | 6962999 | 4867115 (1.32e-04) | | 786. | Tmem180 | P < 1.328e-04 | 6870068 | 6870068 | 5224382 (1.33e-04) | | 787. | Pogz | P < 1.332e-04 | 6899613 | 6899613 | 5319838 (1.33e-04) | 4718510 (8.14e-04) | | 788. | Gpr107 | P < 1.333e-04 | 6876100 | 6876100 | 5008183 (1.33e-04) | | 789. | Gpr44 | P < 1.348e-04 | 6868070 | 6868070 | 4570705 (1.35e-04) | | 790. | C80913 | P < 1.349e-04 | 6966588 | 6966588 | 4308705 (1.35e-04) | | 791. | Eif1a | P < 1.351e-04 | 6860635 | 6860635 | 5473326 (1.35e-04) | | 792. | Pcolce | P < 1.354e-04 | 6942558 | 6942558 | 5428888 (1.35e-04) | | 793. | Nmnat2 | P < 1.362e-04 | 6754102 | 6754102 | 5049420 (1.36e-04) | 4861618 (1.61e-04) | 4317385 (4.54e-04) | | 794. | Tiam2 | P < 1.363e-04 | 6848368 | 6848368 | 4729967 (1.36e-04) | | 795. | Zfp777 | P < 1.363e-04 | 6953369 | 6953369 | 5543654 (1.36e-04) | | 796. | Ryr1 | P < 1.377e-04 | 6966183 | 6966183 | 5481083 (1.38e-04) | 4768586 (2.71e-04) | 5613507 (4.04e-04) | 5030708 (4.68e-04) | | 797. | Psen1 | P < 1.388e-04 | 6796540 | 6796540 | 4578637 (1.39e-04) | | 798. | Dut | P < 1.399e-04 | 6880853 | 6880853 | 4973263 (1.40e-04) | | 799. | Rps6ka1 | P < 1.401e-04 | 6925958 | 6925958 | 4362735 (1.40e-04) | | 800. | Ddx19a | P < 1.409e-04 | 6985340 | 6985340 | 5163488 (1.41e-04) | | 801. | 2610027L16Rik | P < 1.415e-04 | 6819274 | 6819274 | 5438695 (1.42e-04) | | 802. | Rbpms | P < 1.416e-04 | 6981604 | 6981604 | 5422528 (1.42e-04) | | 803. | Piga | P < 1.422e-04 | 7015011 | 7015011 | 4475787 (1.42e-04) | | 804. | NA | P < 1.422e-04 | 6890670 | 6890670 | 5121868 (1.42e-04) | | 805. | Arvcf | P < 1.426e-04 | 6839836 | 6839836 | 5377673 (1.43e-04) | | 806. | Tcea2 | P < 1.430e-04 | 6884294 | 6884294 | 5079797 (1.43e-04) | | 807. | Ercc8 | P < 1.433e-04 | 6810051 | 6810051 | 4678462 (1.43e-04) | | 808. | Alg8 | P < 1.437e-04 | 6962751 | 6962751 | 5276101 (1.44e-04) | | 809. | Fbln7 | P < 1.442e-04 | 6881092 | 6881092 | 5249901 (1.44e-04) | | 810. | Ipo11 | P < 1.444e-04 | 6815792 | 6815792 | 4954760 (1.44e-04) | 5356874 (4.19e-04) | | 811. | Tyw1 | P < 1.450e-04 | 6934668 | 6934668 | 4444819 (1.45e-04) | | 812. | Ss18l1 | P < 1.454e-04 | 6884129 | 6884129 | 5084813 (1.45e-04) | 5072159 (6.76e-04) | | 813. | Lphn3 | P < 1.455e-04 | 6931961 | 6931961 | 5301240 (1.46e-04) | 5552025 (8.26e-04) | | 814. | Klhdc4 | P < 1.466e-04 | 6985886 | 6985886 | 5319636 (1.47e-04) | 5557681 (6.74e-04) | | 815. | Inpp5b | P < 1.469e-04 | 6917045 | 6917045 | 5596392 (1.47e-04) | 5280021 (6.02e-04) | | 816. | Slc32a1 | P < 1.477e-04 | 6882762 | 6882762 | 4553950 (1.48e-04) | | 817. | Npdc1 | P < 1.479e-04 | 6875720 | 6875720 | 4941446 (1.48e-04) | 5387633 (1.52e-04) | | 818. | NA | P < 1.501e-04 | 6855725 | 6855725 | 5005446 (1.50e-04) | | 819. | Lrrc28 | P < 1.502e-04 | 6968027 | 6968027 | 4962983 (1.50e-04) | | 820. | Arsb | P < 1.505e-04 | 6809030 | 6809030 | 4859854 (1.51e-04) | | 821. | Cct2 | P < 1.512e-04 | 6777296 | 6777296 | 5474091 (1.51e-04) | | 822. | Heatr3 | P < 1.514e-04 | 6977971 | 6977971 | 4915668 (1.51e-04) | | 823. | Ikbkb | P < 1.519e-04 | 6980965 | 6980965 | 4597746 (1.52e-04) | | 824. | Nfia | P < 1.521e-04 | 6915619 | 6915619 | 5364513 (1.52e-04) | | 825. | Anp32e | P < 1.525e-04 | 6899722 | 6899722 | 4727782 (1.52e-04) | | 826. | Enox1 | P < 1.533e-04 | 6820425 | 6820425 | 5323850 (1.53e-04) | 4478673 (2.29e-04) | 4470368 (1.03e-03) | | 827. | Suv39h2 | P < 1.534e-04 | 6884351 | 6884351 | 5105311 (1.53e-04) | 4477896 (4.72e-04) | | 828. | Adcy7 | P < 1.536e-04 | 6977975 | 6977975 | 4943282 (1.54e-04) | | 829. | Nktr | P < 1.536e-04 | 6993005 | 6993005 | 5370195 (1.54e-04) | | 830. | Clcn1 | P < 1.542e-04 | 6945782 | 6945782 | 5215453 (1.54e-04) | | 831. | Frmd8 | P < 1.544e-04 | 6871156 | 6871156 | 5574244 (1.54e-04) | | 832. | Siah2 | P < 1.552e-04 | 6905366 | 6905366 | 5105309 (1.55e-04) | | 833. | Stx17 | P < 1.557e-04 | 6913316 | 6913316 | 4626917 (1.56e-04) | | 834. | Tead4 | P < 1.563e-04 | 6957327 | 6957327 | 5445014 (1.56e-04) | | 835. | Vapa | P < 1.567e-04 | 6856674 | 6856674 | 4434506 (1.57e-04) | | 836. | P2rx4 | P < 1.568e-04 | 6934164 | 6934164 | 4316146 (1.57e-04) | | 837. | Aaas | P < 1.572e-04 | 6838730 | 6838730 | 4528535 (1.57e-04) | | 838. | Tulp4 | P < 1.578e-04 | 6848513 | 6848513 | 4390437 (1.58e-04) | | 839. | Tspan10 | P < 1.578e-04 | 6785434 | 6785434 | 5041258 (1.58e-04) | | 840. | Cbs | P < 1.586e-04 | 6854826 | 6854826 | 5456099 (1.59e-04) | 5203348 (8.16e-04) | | 841. | Ddef1 | P < 1.590e-04 | 6836237 | 6836237 | 5027359 (1.59e-04) | 5409989 (8.74e-04) | | 842. | Elf2 | P < 1.594e-04 | 6904953 | 6904953 | 5576825 (1.59e-04) | 4544277 (4.14e-04) | | 843. | Ecel1 | P < 1.602e-04 | 6760417 | 6760417 | 5376713 (1.60e-04) | | 844. | Grhl3 | P < 1.604e-04 | 6926087 | 6926087 | 5612381 (1.60e-04) | | 845. | 6230416J20Rik | P < 1.605e-04 | 6923058 | 6923058 | 4966748 (1.60e-04) | | 846. | Cacnb3 | P < 1.618e-04 | 6833100 | 6833100 | 4459160 (1.62e-04) | | 847. | Tnrc18 | P < 1.622e-04 | 6942847 | 6942847 | 4512858 (1.62e-04) | 4460971 (9.38e-04) | | 848. | Pde2a | P < 1.626e-04 | 6963006 | 6963006 | 5565346 (1.63e-04) | 4547377 (4.07e-04) | | 849. | Bhlhb8 | P < 1.630e-04 | 6935439 | 6935439 | 4533628 (1.63e-04) | | 850. | H13 | P < 1.633e-04 | 6882305 | 6882305 | 5229963 (1.63e-04) | | 851. | Cct5 | P < 1.634e-04 | 6834752 | 6834752 | 4984336 (1.63e-04) | 4926448 (2.79e-04) | 4465590 (3.07e-04) | 4678047 (6.29e-04) | 5204822 (9.69e-04) | | 852. | Accn4 | P < 1.634e-04 | 6750632 | 6750632 | 5354227 (1.63e-04) | 5210372 (3.25e-04) | | 853. | Nol10 | P < 1.650e-04 | 6793674 | 6793674 | 5565388 (1.65e-04) | | 854. | Soat2 | P < 1.653e-04 | 6833394 | 6833394 | 4441146 (1.65e-04) | 4555974 (3.69e-04) | 4883177 (3.83e-04) | 5519852 (1.01e-03) | | 855. | Ppp2r5c | P < 1.655e-04 | 6798067 | 6798067 | 4883203 (1.66e-04) | | 856. | Mthfd1l | P < 1.657e-04 | 6771920 | 6771920 | 5102199 (1.66e-04) | 4781444 (7.91e-04) | | 857. | NA | P < 1.662e-04 | 6989901 | 6989901 | 4841935 (1.66e-04) | 4429743 (4.65e-04) | | 858. | Nucks1 | P < 1.667e-04 | 6753085 | 6753085 | 5061914 (1.67e-04) | | 859. | Arfgap2 | P < 1.670e-04 | 6879040 | 6879040 | 4382447 (1.67e-04) | 4750783 (4.23e-04) | | 860. | Slc7a6 | P < 1.677e-04 | 6978884 | 6978884 | 4807219 (1.68e-04) | 4955488 (4.15e-04) | | 861. | Bat1a | P < 1.678e-04 | 6850132 | 6850132 | 5387538 (1.68e-04) | 5355335 (5.16e-04) | | 862. | Wwp2 | P < 1.679e-04 | 6978968 | 6978968 | 4694664 (1.68e-04) | 4646324 (5.96e-04) | | 863. | Bach1 | P < 1.683e-04 | 6842940 | 6842940 | 4746336 (1.68e-04) | | 864. | Eif2ak4 | P < 1.689e-04 | 6880423 | 6880423 | 4527075 (1.69e-04) | | 865. | Dusp26 | P < 1.690e-04 | 6975050 | 6975050 | 5386580 (1.69e-04) | 4455505 (2.09e-04) | | 866. | Apitd1 | P < 1.696e-04 | 6926934 | 6926934 | 4884953 (1.70e-04) | | 867. | Cited1 | P < 1.698e-04 | 7018585 | 7018585 | 4306091 (1.70e-04) | | 868. | Setd1a | P < 1.700e-04 | 6964352 | 6964352 | 4417320 (1.70e-04) | 4899507 (3.75e-04) | | 869. | Cnot6l | P < 1.707e-04 | 6940146 | 6940146 | 4434020 (1.71e-04) | 4394043 (9.68e-04) | | 870. | Nme1 | P < 1.715e-04 | 6790902 | 6790902 | 4315630 (1.71e-04) | | 871. | Nrd1 | P < 1.721e-04 | 6916221 | 6916221 | 5576583 (1.72e-04) | 5255836 (9.51e-04) | | 872. | 2810022L02Rik | P < 1.732e-04 | 6749691 | 6749691 | 5071090 (1.73e-04) | 5245773 (5.82e-04) | | 873. | Dalrd3 | P < 1.735e-04 | 6992350 | 6992350 | 5046505 (1.74e-04) | | 874. | Rnf44 | P < 1.737e-04 | 6813332 | 6813332 | 5420575 (1.74e-04) | | 875. | Rfx3 | P < 1.739e-04 | 6872444 | 6872444 | 4944916 (1.74e-04) | | 876. | Armc1 | P < 1.743e-04 | 6903480 | 6903480 | 4323915 (1.74e-04) | | 877. | Ddx39 | P < 1.743e-04 | 6977687 | 6977687 | 4681932 (1.74e-04) | | 878. | Sfxn1 | P < 1.758e-04 | 6807154 | 6807154 | 5239089 (1.76e-04) | | 879. | Prph | P < 1.769e-04 | 6833146 | 6833146 | 5453854 (1.77e-04) | 5028471 (2.52e-04) | | 880. | Ube2o | P < 1.786e-04 | 6792527 | 6792527 | 5588339 (1.79e-04) | | 881. | Dennd4b | P < 1.788e-04 | 6899335 | 6899335 | 4662637 (1.79e-04) | | 882. | Sqle | P < 1.799e-04 | 6830761 | 6830761 | 4741147 (1.80e-04) | 5423875 (5.12e-04) | | 883. | Cox11 | P < 1.806e-04 | 6783500 | 6783500 | 5394260 (1.81e-04) | | 884. | Sidt2 | P < 1.811e-04 | 6995259 | 6995259 | 5150432 (1.81e-04) | | 885. | Ifnar2 | P < 1.813e-04 | 6843177 | 6843177 | 5442762 (1.81e-04) | 4813066 (5.87e-04) | | 886. | Sez6 | P < 1.814e-04 | 6782626 | 6782626 | 5275945 (1.81e-04) | | 887. | Mapk13 | P < 1.836e-04 | 6849571 | 6849571 | 4465909 (1.84e-04) | | 888. | St8sia6 | P < 1.843e-04 | 6884820 | 6884820 | 4769603 (1.84e-04) | 5303643 (3.23e-04) | 4427257 (5.55e-04) | | 889. | Rad54l2 | P < 1.844e-04 | 6998595 | 6998595 | 5270412 (1.84e-04) | | 890. | Napsa | P < 1.850e-04 | 6960287 | 6960287 | 4704333 (1.85e-04) | 4916461 (7.58e-04) | 4358402 (1.02e-03) | 5390768 (1.06e-03) | | 891. | Hsd3b6 | P < 1.864e-04 | 6907433 | 6907433 | 4859557 (1.86e-04) | | 892. | Nkain4 | P < 1.868e-04 | 6894245 | 6894245 | 4915357 (1.87e-04) | | 893. | Bxdc5 | P < 1.877e-04 | 6910279 | 6910279 | 4719812 (1.88e-04) | | 894. | Ctgf | P < 1.884e-04 | 6766623 | 6766623 | 5137960 (1.88e-04) | | 895. | St8sia5 | P < 1.887e-04 | 6862281 | 6862281 | 5516137 (1.89e-04) | | 896. | Pde1b | P < 1.893e-04 | 6833516 | 6833516 | 5430579 (1.89e-04) | 5321669 (4.88e-04) | | 897. | Itga3 | P < 1.894e-04 | 6791017 | 6791017 | 5330284 (1.89e-04) | | 898. | Phc3 | P < 1.894e-04 | 6903946 | 6903946 | 4611374 (1.89e-04) | 4978992 (2.90e-04) | 4891108 (6.08e-04) | 5373093 (6.19e-04) | | 899. | Scrib | P < 1.903e-04 | 6836794 | 6836794 | 5199130 (1.90e-04) | | 900. | Haghl | P < 1.910e-04 | 6854433 | 6854433 | 4689950 (1.91e-04) | | 901. | Prpf4b | P < 1.911e-04 | 6806162 | 6806162 | 4622935 (1.91e-04) | | 902. | Rnaseh2b | P < 1.913e-04 | 6819662 | 6819662 | 4515447 (1.91e-04) | | 903. | Ahsa1 | P < 1.914e-04 | 6796784 | 6796784 | 4390171 (1.91e-04) | | 904. | Spag8 | P < 1.921e-04 | 6921172 | 6921172 | 5502755 (1.92e-04) | | 905. | Dpp4 | P < 1.924e-04 | 6887179 | 6887179 | 4694893 (1.92e-04) | | 906. | Nup153 | P < 1.925e-04 | 6812968 | 6812968 | 5093633 (1.93e-04) | 5599217 (3.53e-04) | | 907. | Eme1 | P < 1.930e-04 | 6790966 | 6790966 | 5430077 (1.93e-04) | | 908. | Sdad1 | P < 1.931e-04 | 6939986 | 6939986 | 5261029 (1.93e-04) | | 909. | Scp2 | P < 1.933e-04 | 6924366 | 6924366 | 4788877 (1.93e-04) | | 910. | St14 | P < 1.955e-04 | 6994353 | 6994353 | 5149961 (1.96e-04) | 5342255 (7.26e-04) | | 911. | Nampt | P < 1.958e-04 | 6794293 | 6794293 | 5159572 (1.96e-04) | 4837558 (6.83e-04) | 4885345 (9.48e-04) | | 912. | Ptpdc1 | P < 1.965e-04 | 6813070 | 6813070 | 5450840 (1.97e-04) | | 913. | 6430548M08Rik | P < 1.967e-04 | 6979527 | 6979527 | 5209415 (1.97e-04) | | 914. | Pmpca | P < 1.972e-04 | 6875792 | 6875792 | 5224547 (1.97e-04) | | 915. | 4930452B06Rik | P < 1.975e-04 | 6822485 | 6822485 | 4307745 (1.98e-04) | | 916. | Hbb-bh1 | P < 1.976e-04 | 6970005 | 6970005 | 4590584 (1.98e-04) | | 917. | Tmem93 | P < 1.977e-04 | 6789584 | 6789584 | 4995824 (1.98e-04) | | 918. | Alkbh1 | P < 1.982e-04 | 6802555 | 6802555 | 4358570 (1.98e-04) | | 919. | Plxna3 | P < 1.983e-04 | 7012010 | 7012010 | 4808216 (1.98e-04) | | 920. | Phc2 | P < 1.995e-04 | 6917249 | 6917249 | 5142497 (1.99e-04) | 4438256 (7.33e-04) | | 921. | NA | P < 1.997e-04 | 6840086 | 6840086 | 5096103 (2.00e-04) | | 922. | Athl1 | P < 2.000e-04 | 6965143 | 6965143 | 4347284 (2.00e-04) | | 923. | Tmem63b | P < 2.002e-04 | 6855625 | 6855625 | 5440971 (2.00e-04) | | 924. | Ptprs | P < 2.005e-04 | 6856231 | 6856231 | 4790356 (2.00e-04) | 4865020 (5.31e-04) | 5334463 (1.04e-03) | | 925. | Skiv2l2 | P < 2.013e-04 | 6816148 | 6816148 | 4351812 (2.01e-04) | 4345042 (4.06e-04) | | 926. | Crtc1 | P < 2.018e-04 | 6983184 | 6983184 | 5550141 (2.02e-04) | | 927. | Pa2g4 | P < 2.025e-04 | 6778043 | 6778043 | 5491164 (2.03e-04) | | 928. | Gpt2 | P < 2.026e-04 | 6977814 | 6977814 | 5108816 (2.03e-04) | | 929. | AU040829 | P < 2.036e-04 | 6789047 | 6789047 | 5243897 (2.04e-04) | | 930. | Fgfr3 | P < 2.040e-04 | 6929817 | 6929817 | 5208447 (2.04e-04) | | 931. | Chtf18 | P < 2.040e-04 | 6854430 | 6854430 | 4354467 (2.04e-04) | | 932. | Ssbp2 | P < 2.042e-04 | 6808876 | 6808876 | 5088427 (2.04e-04) | | 933. | Snx29 | P < 2.046e-04 | 6839420 | 6839420 | 4603489 (2.05e-04) | | 934. | Gga3 | P < 2.046e-04 | 6792452 | 6792452 | 4349996 (2.05e-04) | 4754055 (2.82e-04) | 4647575 (7.59e-04) | | 935. | Glg1 | P < 2.049e-04 | 6985355 | 6985355 | 5283158 (2.05e-04) | | 936. | Mst1r | P < 2.051e-04 | 6992274 | 6992274 | 4387384 (2.05e-04) | | 937. | Tbl1x | P < 2.056e-04 | 7012151 | 7012151 | 5434041 (2.06e-04) | | 938. | Sec16a | P < 2.057e-04 | 6885487 | 6885487 | 4806360 (2.06e-04) | | 939. | Setd5 | P < 2.073e-04 | 6949064 | 6949064 | 4942717 (2.07e-04) | | 940. | 5730410E15Rik | P < 2.079e-04 | 6835428 | 6835428 | 5492469 (2.08e-04) | | 941. | Vars | P < 2.082e-04 | 6850092 | 6850092 | 4513829 (2.08e-04) | 5266386 (3.35e-04) | 5299926 (7.15e-04) | 4524682 (9.37e-04) | | 942. | Trpc6 | P < 2.087e-04 | 6986775 | 6986775 | 4491049 (2.09e-04) | | 943. | Hrc | P < 2.091e-04 | 6960358 | 6960358 | 5433485 (2.09e-04) | 4485693 (6.54e-04) | | 944. | Golga2 | P < 2.102e-04 | 6876181 | 6876181 | 5189440 (2.10e-04) | | 945. | Tcta | P < 2.103e-04 | 6998676 | 6998676 | 4649001 (2.10e-04) | 5173031 (9.52e-04) | | 946. | Dcn | P < 2.104e-04 | 6770064 | 6770064 | 4544820 (2.10e-04) | 5194203 (3.95e-04) | | 947. | Sh3tc1 | P < 2.107e-04 | 6937442 | 6937442 | 4704115 (2.11e-04) | | 948. | Hisppd2a | P < 2.110e-04 | 6890382 | 6890382 | 5289389 (2.11e-04) | | 949. | Hacl1 | P < 2.112e-04 | 6823742 | 6823742 | 4376424 (2.11e-04) | | 950. | Lamc3 | P < 2.118e-04 | 6876159 | 6876159 | 4517268 (2.12e-04) | | 951. | Cul1 | P < 2.131e-04 | 6945935 | 6945935 | 5541274 (2.13e-04) | | 952. | Sep15 | P < 2.146e-04 | 6902122 | 6902122 | 5195492 (2.15e-04) | | 953. | Ddx19b | P < 2.149e-04 | 6985343 | 6985343 | 4584224 (2.15e-04) | | 954. | 2210404J11Rik | P < 2.153e-04 | 6848813 | 6848813 | 5438441 (2.15e-04) | | 955. | Cd97 | P < 2.168e-04 | 6983799 | 6983799 | 4791132 (2.17e-04) | | 956. | Sphk2 | P < 2.180e-04 | 6967004 | 6967004 | 4627329 (2.18e-04) | | 957. | Eif5 | P < 2.188e-04 | 6798216 | 6798216 | 4370216 (2.19e-04) | | 958. | Neu1 | P < 2.194e-04 | 6850080 | 6850080 | 5563281 (2.19e-04) | | 959. | Exosc8 | P < 2.206e-04 | 6905192 | 6905192 | 5534000 (2.21e-04) | | 960. | Syngr2 | P < 2.207e-04 | 6785299 | 6785299 | 5445992 (2.21e-04) | | 961. | Cds2 | P < 2.207e-04 | 6881306 | 6881306 | 5390089 (2.21e-04) | 4764913 (7.35e-04) | | 962. | Gspt1 | P < 2.213e-04 | 6843953 | 6843953 | 4731890 (2.21e-04) | 4962300 (2.69e-04) | 5140264 (3.00e-04) | 4908669 (5.01e-04) | | 963. | Actg2 | P < 2.214e-04 | 6955035 | 6955035 | 5533117 (2.21e-04) | 4947994 (2.54e-04) | | 964. | Creb5 | P < 2.216e-04 | 6946313 | 6946313 | 5228615 (2.22e-04) | 4753759 (4.29e-04) | | 965. | Abcd4 | P < 2.220e-04 | 6802333 | 6802333 | 5579777 (2.22e-04) | 4454941 (2.87e-04) | | 966. | BC066135 | P < 2.231e-04 | 6894182 | 6894182 | 5416527 (2.23e-04) | | 967. | Ogfod2 | P < 2.233e-04 | 6934273 | 6934273 | 5274426 (2.23e-04) | | 968. | Myo5b | P < 2.238e-04 | 6862094 | 6862094 | 4554035 (2.24e-04) | 4791853 (3.97e-04) | 5004765 (3.98e-04) | 5363535 (6.25e-04) | | 969. | Rps6kb2 | P < 2.239e-04 | 6870978 | 6870978 | 5523703 (2.24e-04) | | 970. | Card10 | P < 2.240e-04 | 6837046 | 6837046 | 4901625 (2.24e-04) | | 971. | NA | P < 2.252e-04 | 6934216 | 6934216 | 4481818 (2.25e-04) | | 972. | Cdh15 | P < 2.252e-04 | 6979655 | 6979655 | 5371964 (2.25e-04) | | 973. | Hebp2 | P < 2.253e-04 | 6772408 | 6772408 | 4860252 (2.25e-04) | | 974. | Pld1 | P < 2.267e-04 | 6896406 | 6896406 | 4799284 (2.27e-04) | 5123443 (9.70e-04) | | 975. | Arid3a | P < 2.285e-04 | 6769165 | 6769165 | 5460879 (2.28e-04) | 5267020 (1.04e-03) | | 976. | Prkar1b | P < 2.298e-04 | 6942655 | 6942655 | 4528739 (2.30e-04) | | 977. | Adamts7 | P < 2.303e-04 | 6991267 | 6991267 | 4703005 (2.30e-04) | | 978. | Calr | P < 2.304e-04 | 6983879 | 6983879 | 5317665 (2.30e-04) | | 979. | Lrp8 | P < 2.313e-04 | 6916143 | 6916143 | 4929384 (2.31e-04) | | 980. | Ascl1 | P < 2.326e-04 | 6775783 | 6775783 | 5204131 (2.33e-04) | | 981. | Etfa | P < 2.338e-04 | 6995817 | 6995817 | 4866932 (2.34e-04) | 5189055 (4.80e-04) | | 982. | Lhx6 | P < 2.338e-04 | 6886075 | 6886075 | 4753316 (2.34e-04) | 4593283 (9.11e-04) | | 983. | 3110057O12Rik | P < 2.339e-04 | 6897008 | 6897008 | 5170260 (2.34e-04) | | 984. | Arid4b | P < 2.339e-04 | 6804919 | 6804919 | 5237662 (2.34e-04) | | 985. | Add3 | P < 2.349e-04 | 6870424 | 6870424 | 5117020 (2.35e-04) | | 986. | Limk2 | P < 2.351e-04 | 6785601 | 6785601 | 5040068 (2.35e-04) | | 987. | Cdh1 | P < 2.353e-04 | 6978923 | 6978923 | 4676439 (2.35e-04) | | 988. | Heatr5b | P < 2.358e-04 | 6857434 | 6857434 | 4893504 (2.36e-04) | | 989. | Narg1 | P < 2.361e-04 | 6897349 | 6897349 | 4341624 (2.36e-04) | | 990. | 0610040J01Rik | P < 2.363e-04 | 6931168 | 6931168 | 5156471 (2.36e-04) | | 991. | Npr2 | P < 2.373e-04 | 6913020 | 6913020 | 4616662 (2.37e-04) | 4472931 (5.05e-04) | | 992. | Bnip3 | P < 2.374e-04 | 6971987 | 6971987 | 5089793 (2.37e-04) | | 993. | Gpr156 | P < 2.375e-04 | 6840823 | 6840823 | 5299324 (2.37e-04) | | 994. | Utrn | P < 2.375e-04 | 6772193 | 6772193 | 4854784 (2.37e-04) | 4669447 (4.03e-04) | 4550397 (5.27e-04) | 4382037 (8.62e-04) | 5001664 (9.94e-04) | | 995. | Exosc5 | P < 2.382e-04 | 6959228 | 6959228 | 5430576 (2.38e-04) | | 996. | Prmt1 | P < 2.382e-04 | 6966922 | 6966922 | 5233422 (2.38e-04) | 5098846 (6.88e-04) | | 997. | Ehbp1 | P < 2.384e-04 | 6786586 | 6786586 | 5477817 (2.38e-04) | 5133045 (7.86e-04) | | 998. | Ccdc3 | P < 2.385e-04 | 6874653 | 6874653 | 4985491 (2.38e-04) | 5076685 (4.09e-04) | | 999. | 4121402D02Rik | P < 2.385e-04 | 6784048 | 6784048 | 5062453 (2.39e-04) | 4404893 (4.67e-04) | | 1000. | Ubqln1 | P < 2.427e-04 | 6813555 | 6813555 | 5433546 (2.43e-04) | 4462442 (3.49e-04) | | 1001. | Llgl1 | P < 2.427e-04 | 6781456 | 6781456 | 4643584 (2.43e-04) | | 1002. | Rgs9 | P < 2.433e-04 | 6792079 | 6792079 | 5173956 (2.43e-04) | | 1003. | Ccdc95 | P < 2.436e-04 | 6971303 | 6971303 | 5602383 (2.44e-04) | | 1004. | Rfx2 | P < 2.439e-04 | 6856257 | 6856257 | 5184710 (2.44e-04) | 4948234 (3.29e-04) | | 1005. | Fhod1 | P < 2.447e-04 | 6984966 | 6984966 | 5279486 (2.45e-04) | | 1006. | Arhgef4 | P < 2.448e-04 | 6748553 | 6748553 | 4354006 (2.45e-04) | | 1007. | Os9 | P < 2.452e-04 | 6777906 | 6777906 | 4506120 (2.45e-04) | | 1008. | Baz2a | P < 2.453e-04 | 6771628 | 6771628 | 4892344 (2.45e-04) | | 1009. | Sema4a | P < 2.468e-04 | 6906749 | 6906749 | 4322994 (2.47e-04) | 4422652 (4.63e-04) | 5612242 (7.87e-04) | | 1010. | B2m | P < 2.472e-04 | 6880670 | 6880670 | 4513606 (2.47e-04) | | 1011. | Kif22 | P < 2.488e-04 | 6971320 | 6971320 | 4478057 (2.49e-04) | | 1012. | Pdcd11 | P < 2.491e-04 | 6870130 | 6870130 | 5304614 (2.49e-04) | | 1013. | Gpi1 | P < 2.494e-04 | 6966425 | 6966425 | 5304129 (2.49e-04) | 5512883 (4.38e-04) | | 1014. | Pbx1 | P < 2.498e-04 | 6763937 | 6763937 | 4538548 (2.50e-04) | 5169000 (2.56e-04) | | 1015. | Dgkk | P < 2.505e-04 | 7009748 | 7009748 | 4669782 (2.51e-04) | 5121702 (7.81e-04) | | 1016. | Crtac1 | P < 2.511e-04 | 6873187 | 6873187 | 4437159 (2.51e-04) | 5255865 (4.80e-04) | | 1017. | Irs1 | P < 2.516e-04 | 6760177 | 6760177 | 5006341 (2.52e-04) | | 1018. | Plekhj1 | P < 2.535e-04 | 6775401 | 6775401 | 5083706 (2.54e-04) | | 1019. | Opa1 | P < 2.536e-04 | 6840369 | 6840369 | 4837852 (2.54e-04) | 5007211 (3.46e-04) | | 1020. | Cgnl1 | P < 2.536e-04 | 6996703 | 6996703 | 5341675 (2.54e-04) | 4946202 (5.16e-04) | 5356323 (5.77e-04) | | 1021. | Plod1 | P < 2.536e-04 | 6926855 | 6926855 | 5312143 (2.54e-04) | | 1022. | Slc41a3 | P < 2.539e-04 | 6947939 | 6947939 | 4502989 (2.54e-04) | | 1023. | Gpc1 | P < 2.545e-04 | 6751634 | 6751634 | 4569960 (2.54e-04) | | 1024. | Rassf4 | P < 2.561e-04 | 6956765 | 6956765 | 4405337 (2.56e-04) | | 1025. | Neo1 | P < 2.562e-04 | 6996018 | 6996018 | 4347936 (2.56e-04) | | 1026. | Myh9 | P < 2.565e-04 | 6836973 | 6836973 | 5183366 (2.57e-04) | 5443177 (6.71e-04) | | 1027. | Galnt2 | P < 2.573e-04 | 6979772 | 6979772 | 5402658 (2.57e-04) | | 1028. | Tpmt | P < 2.573e-04 | 6812983 | 6812983 | 4583124 (2.57e-04) | | 1029. | Aco1 | P < 2.592e-04 | 6912820 | 6912820 | 4495642 (2.59e-04) | | 1030. | Slc25a37 | P < 2.598e-04 | 6825638 | 6825638 | 5087878 (2.60e-04) | | 1031. | Rnasen | P < 2.608e-04 | 6828862 | 6828862 | 4819348 (2.61e-04) | | 1032. | Gsx1 | P < 2.618e-04 | 6935579 | 6935579 | 5005330 (2.62e-04) | | 1033. | Ap1gbp1 | P < 2.635e-04 | 6783057 | 6783057 | 5219213 (2.63e-04) | 5476290 (7.00e-04) | 5470630 (8.97e-04) | | 1034. | Rtel1 | P < 2.636e-04 | 6884262 | 6884262 | 5147123 (2.64e-04) | | 1035. | Mfap3 | P < 2.637e-04 | 6781209 | 6781209 | 4858530 (2.64e-04) | | 1036. | Dus3l | P < 2.640e-04 | 6851273 | 6851273 | 5014278 (2.64e-04) | 4862777 (2.68e-04) | | 1037. | Brd4 | P < 2.656e-04 | 6854868 | 6854868 | 5541384 (2.66e-04) | | 1038. | Pla2g4a | P < 2.659e-04 | 6762944 | 6762944 | 5430165 (2.66e-04) | | 1039. | Mff | P < 2.662e-04 | 6751070 | 6751070 | 4362032 (2.66e-04) | 4324626 (2.98e-04) | | 1040. | 2310040C09Rik | P < 2.665e-04 | 6781526 | 6781526 | 5147276 (2.66e-04) | | 1041. | Aph1b | P < 2.666e-04 | 6996432 | 6996432 | 5513087 (2.67e-04) | | 1042. | Gp1bb | P < 2.689e-04 | 6844348 | 6844348 | 5449353 (2.69e-04) | | 1043. | Ttc27 | P < 2.695e-04 | 6852225 | 6852225 | 4583202 (2.69e-04) | | 1044. | Sqstm1 | P < 2.698e-04 | 6788014 | 6788014 | 5520468 (2.70e-04) | | 1045. | Ralgps1 | P < 2.700e-04 | 6885930 | 6885930 | 5562530 (2.70e-04) | 5256051 (7.68e-04) | | 1046. | Nrcam | P < 2.705e-04 | 6794766 | 6794766 | 5103210 (2.70e-04) | | 1047. | 5830418K08Rik | P < 2.710e-04 | 6993501 | 6993501 | 5246717 (2.71e-04) | | 1048. | Tmem143 | P < 2.725e-04 | 6960423 | 6960423 | 4490592 (2.73e-04) | | 1049. | Ltbp1 | P < 2.736e-04 | 6852229 | 6852229 | 4427564 (2.74e-04) | 4397028 (7.39e-04) | | 1050. | Flii | P < 2.742e-04 | 6788784 | 6788784 | 4545475 (2.74e-04) | 5515753 (7.39e-04) | 4604115 (9.38e-04) | | 1051. | Ppp1r9a | P < 2.743e-04 | 6943862 | 6943862 | 5452022 (2.74e-04) | | 1052. | Bok | P < 2.765e-04 | 6751709 | 6751709 | 4852698 (2.76e-04) | | 1053. | Tiam1 | P < 2.769e-04 | 6847748 | 6847748 | 5029340 (2.77e-04) | | 1054. | l7Rn6 | P < 2.771e-04 | 6969253 | 6969253 | 5344334 (2.77e-04) | | 1055. | A330021E22Rik | P < 2.772e-04 | 6935983 | 6935983 | 5161232 (2.77e-04) | | 1056. | Sema5a | P < 2.774e-04 | 6829591 | 6829591 | 5124810 (2.77e-04) | | 1057. | Btg2 | P < 2.777e-04 | 6762345 | 6762345 | 4910411 (2.78e-04) | | 1058. | Bicd2 | P < 2.782e-04 | 6806938 | 6806938 | 5563130 (2.78e-04) | | 1059. | Slc5a2 | P < 2.786e-04 | 6964396 | 6964396 | 5472375 (2.79e-04) | | 1060. | Ttc25 | P < 2.787e-04 | 6784202 | 6784202 | 5326891 (2.79e-04) | | 1061. | Cacng4 | P < 2.787e-04 | 6792024 | 6792024 | 4600041 (2.79e-04) | | 1062. | Timm13 | P < 2.787e-04 | 6775410 | 6775410 | 4969197 (2.79e-04) | | 1063. | Sema6b | P < 2.794e-04 | 6856204 | 6856204 | 4541190 (2.79e-04) | 5115607 (4.75e-04) | | 1064. | Ctsc | P < 2.799e-04 | 6962376 | 6962376 | 4478633 (2.80e-04) | | 1065. | Usp15 | P < 2.806e-04 | 6777684 | 6777684 | 4836581 (2.81e-04) | | 1066. | Sh3gl3 | P < 2.813e-04 | 6962107 | 6962107 | 5402780 (2.81e-04) | 4935945 (6.15e-04) | | 1067. | Rnf38 | P < 2.814e-04 | 6921211 | 6921211 | 4465149 (2.81e-04) | | 1068. | Timeless | P < 2.825e-04 | 6771636 | 6771636 | 5217629 (2.83e-04) | | 1069. | Itpr1 | P < 2.829e-04 | 6948906 | 6948906 | 5140799 (2.83e-04) | 4486289 (9.00e-04) | | 1070. | Rrp1b | P < 2.846e-04 | 6849845 | 6849845 | 4450898 (2.85e-04) | | 1071. | Clic1 | P < 2.854e-04 | 6850097 | 6850097 | 4599392 (2.85e-04) | | 1072. | NA | P < 2.856e-04 | 6836797 | 6836797 | 4705310 (2.86e-04) | | 1073. | Abcf1 | P < 2.857e-04 | 6855149 | 6855149 | 5218435 (2.86e-04) | | 1074. | Wwox | P < 2.863e-04 | 6979320 | 6979320 | 4417372 (2.86e-04) | | 1075. | Nefl | P < 2.876e-04 | 6819974 | 6819974 | 4721605 (2.88e-04) | | 1076. | Trim33 | P < 2.876e-04 | 6900100 | 6900100 | 5117038 (2.88e-04) | | 1077. | Angpt1 | P < 2.925e-04 | 6835353 | 6835353 | 5320025 (2.93e-04) | | 1078. | Dmgdh | P < 2.926e-04 | 6809022 | 6809022 | 4903730 (2.93e-04) | 5228175 (4.27e-04) | | 1079. | Hook2 | P < 2.938e-04 | 6977779 | 6977779 | 5397963 (2.94e-04) | | 1080. | Lca5 | P < 2.952e-04 | 6997367 | 6997367 | 5443247 (2.95e-04) | 5601659 (7.78e-04) | | 1081. | Heatr1 | P < 2.963e-04 | 6804849 | 6804849 | 5240519 (2.96e-04) | 4991494 (5.69e-04) | 4931303 (5.82e-04) | | 1082. | Ccdc60 | P < 2.981e-04 | 6941364 | 6941364 | 4552424 (2.98e-04) | | 1083. | Ptpru | P < 2.991e-04 | 6925762 | 6925762 | 4631698 (2.99e-04) | | 1084. | Stat5a | P < 3.012e-04 | 6784218 | 6784218 | 5578576 (3.01e-04) | 5360398 (4.26e-04) | 5321714 (4.26e-04) | | 1085. | Lamp1 | P < 3.019e-04 | 6974138 | 6974138 | 5079112 (3.02e-04) | | 1086. | Trp53 | P < 3.021e-04 | 6782031 | 6782031 | 5130264 (3.02e-04) | | 1087. | Map3k6 | P < 3.029e-04 | 6917577 | 6917577 | 5510142 (3.03e-04) | | 1088. | Vps26a | P < 3.031e-04 | 6774399 | 6774399 | 4308525 (3.03e-04) | | 1089. | Foxo3a | P < 3.035e-04 | 6773545 | 6773545 | 5189583 (3.04e-04) | | 1090. | Plac9 | P < 3.061e-04 | 6823414 | 6823414 | 4738285 (3.06e-04) | | 1091. | Tiparp | P < 3.061e-04 | 6898076 | 6898076 | 5150923 (3.06e-04) | | 1092. | Ankfy1 | P < 3.062e-04 | 6782256 | 6782256 | 4861990 (3.06e-04) | | 1093. | Ddx54 | P < 3.067e-04 | 6933987 | 6933987 | 5213854 (3.07e-04) | | 1094. | D4Wsu114e | P < 3.073e-04 | 6926851 | 6926851 | 4816679 (3.07e-04) | 5426541 (3.61e-04) | | 1095. | Rufy1 | P < 3.074e-04 | 6788025 | 6788025 | 4412487 (3.07e-04) | | 1096. | Palb2 | P < 3.100e-04 | 6971029 | 6971029 | 5437925 (3.10e-04) | | 1097. | Shc1 | P < 3.106e-04 | 6899249 | 6899249 | 5294395 (3.11e-04) | | 1098. | Stoml1 | P < 3.109e-04 | 6989452 | 6989452 | 5064510 (3.11e-04) | | 1099. | Zbtb7b | P < 3.120e-04 | 6906834 | 6906834 | 5486804 (3.12e-04) | | 1100. | Txndc17 | P < 3.127e-04 | 6782224 | 6782224 | 5031206 (3.13e-04) | | 1101. | Lhfpl4 | P < 3.129e-04 | 6956537 | 6956537 | 4495229 (3.13e-04) | | 1102. | Dmpk | P < 3.145e-04 | 6958984 | 6958984 | 5170674 (3.14e-04) | | 1103. | 4732479N06Rik | P < 3.147e-04 | 7019504 | 7019504 | 5033440 (3.15e-04) | | 1104. | Prkcbp1 | P < 3.150e-04 | 6893020 | 6893020 | 4847322 (3.15e-04) | | 1105. | Iars | P < 3.153e-04 | 6806963 | 6806963 | 5452088 (3.15e-04) | 4668903 (3.62e-04) | | 1106. | Yod1 | P < 3.174e-04 | 6752980 | 6752980 | 4764828 (3.17e-04) | | 1107. | Ptpn13 | P < 3.175e-04 | 6933014 | 6933014 | 5544161 (3.17e-04) | 4855521 (9.97e-04) | | 1108. | C130038G02Rik | P < 3.180e-04 | 6935630 | 6935630 | 4574450 (3.18e-04) | | 1109. | Tacc3 | P < 3.183e-04 | 6929816 | 6929816 | 4443229 (3.18e-04) | | 1110. | Arid1a | P < 3.185e-04 | 6925936 | 6925936 | 4383482 (3.18e-04) | | 1111. | Fubp1 | P < 3.208e-04 | 6902493 | 6902493 | 5463633 (3.21e-04) | | 1112. | Tspan31 | P < 3.221e-04 | 6777902 | 6777902 | 4568166 (3.22e-04) | | 1113. | 1110007A13Rik | P < 3.243e-04 | 6971466 | 6971466 | 5154980 (3.24e-04) | 4980896 (9.06e-04) | | 1114. | Jmjd6 | P < 3.245e-04 | 6792553 | 6792553 | 4863145 (3.24e-04) | 4670925 (4.16e-04) | | 1115. | Col11a2 | P < 3.248e-04 | 6850001 | 6850001 | 4751426 (3.25e-04) | 4379584 (7.74e-04) | | 1116. | Cyp26b1 | P < 3.259e-04 | 6955111 | 6955111 | 4510291 (3.26e-04) | | 1117. | AI595406 | P < 3.260e-04 | 6787281 | 6787281 | 4683024 (3.26e-04) | | 1118. | Hspa5 | P < 3.266e-04 | 6876342 | 6876342 | 5118308 (3.27e-04) | | 1119. | Gdi1 | P < 3.269e-04 | 7012007 | 7012007 | 5371345 (3.27e-04) | | 1120. | Ssbp3 | P < 3.273e-04 | 6916095 | 6916095 | 4962226 (3.27e-04) | | 1121. | Cmklr1 | P < 3.276e-04 | 6941173 | 6941173 | 4935250 (3.28e-04) | | 1122. | Eif5a | P < 3.278e-04 | 6789365 | 6789365 | 5202149 (3.28e-04) | | 1123. | Acvr1 | P < 3.320e-04 | 6886957 | 6886957 | 4949405 (3.32e-04) | | 1124. | Ucp2 | P < 3.330e-04 | 6962935 | 6962935 | 5031585 (3.33e-04) | | 1125. | Osr2 | P < 3.332e-04 | 6829722 | 6829722 | 5471947 (3.33e-04) | 4325335 (4.01e-04) | | 1126. | Tnxb | P < 3.333e-04 | 6850066 | 6850066 | 5596331 (3.33e-04) | | 1127. | Qsox1 | P < 3.333e-04 | 6763235 | 6763235 | 5578655 (3.33e-04) | | 1128. | Ptgr1 | P < 3.334e-04 | 6922026 | 6922026 | 5041972 (3.33e-04) | | 1129. | Tkt | P < 3.342e-04 | 6817930 | 6817930 | 4339399 (3.34e-04) | 4681940 (5.00e-04) | | 1130. | Ift140 | P < 3.344e-04 | 6849339 | 6849339 | 5417969 (3.34e-04) | 4747624 (4.19e-04) | | 1131. | Nphp4 | P < 3.354e-04 | 6919021 | 6919021 | 5097567 (3.35e-04) | 4397377 (6.25e-04) | | 1132. | Ipo4 | P < 3.366e-04 | 6824829 | 6824829 | 4691948 (3.37e-04) | | 1133. | Zdhhc20 | P < 3.379e-04 | 6824980 | 6824980 | 4661591 (3.38e-04) | | 1134. | Adipor2 | P < 3.387e-04 | 6956912 | 6956912 | 5518332 (3.39e-04) | | 1135. | Pet112l | P < 3.388e-04 | 6898897 | 6898897 | 5156424 (3.39e-04) | | 1136. | Pdk2 | P < 3.392e-04 | 6791015 | 6791015 | 4640365 (3.39e-04) | | 1137. | Gstk1 | P < 3.395e-04 | 6945775 | 6945775 | 4418427 (3.40e-04) | 4853670 (3.77e-04) | | 1138. | Saps2 | P < 3.402e-04 | 6832564 | 6832564 | 5015552 (3.40e-04) | 4676712 (5.21e-04) | 5591329 (7.31e-04) | | 1139. | Actn1 | P < 3.409e-04 | 6802098 | 6802098 | 5597164 (3.41e-04) | | 1140. | Stab1 | P < 3.415e-04 | 6823709 | 6823709 | 4695862 (3.42e-04) | | 1141. | Ptk2 | P < 3.421e-04 | 6836602 | 6836602 | 4675762 (3.42e-04) | | 1142. | Pde8a | P < 3.429e-04 | 6962054 | 6962054 | 4842944 (3.43e-04) | | 1143. | Ect2 | P < 3.438e-04 | 6903753 | 6903753 | 5577223 (3.44e-04) | | 1144. | Ccdc106 | P < 3.441e-04 | 6972612 | 6972612 | 5538913 (3.44e-04) | | 1145. | Satb2 | P < 3.449e-04 | 6758862 | 6758862 | 4610598 (3.45e-04) | | 1146. | Kif15 | P < 3.451e-04 | 6993098 | 6993098 | 5302864 (3.45e-04) | 4539384 (9.71e-04) | | 1147. | BC033915 | P < 3.462e-04 | 6988764 | 6988764 | 4833632 (3.46e-04) | | 1148. | Ccdc43 | P < 3.469e-04 | 6791626 | 6791626 | 4655975 (3.47e-04) | | 1149. | Sharpin | P < 3.469e-04 | 6836815 | 6836815 | 5310647 (3.47e-04) | | 1150. | NA | P < 3.472e-04 | 6973283 | 6973283 | 5550773 (3.47e-04) | | 1151. | Bcas3 | P < 3.479e-04 | 6783182 | 6783182 | 4717722 (3.48e-04) | | 1152. | Vegfa | P < 3.482e-04 | 6855659 | 6855659 | 4412508 (3.48e-04) | | 1153. | Drd4 | P < 3.482e-04 | 6965187 | 6965187 | 5241330 (3.48e-04) | 5170751 (9.85e-04) | | 1154. | Mark1 | P < 3.489e-04 | 6764841 | 6764841 | 4951889 (3.49e-04) | 5403056 (3.90e-04) | | 1155. | Lhfpl2 | P < 3.496e-04 | 6809047 | 6809047 | 5252652 (3.50e-04) | | 1156. | Rbm28 | P < 3.500e-04 | 6952337 | 6952337 | 5400453 (3.50e-04) | | 1157. | 2810405K02Rik | P < 3.504e-04 | 6927227 | 6927227 | 4451396 (3.50e-04) | | 1158. | Acox3 | P < 3.512e-04 | 6929908 | 6929908 | 4972922 (3.51e-04) | 5287559 (7.74e-04) | 4733609 (7.86e-04) | 4901034 (8.96e-04) | | 1159. | Xrcc3 | P < 3.526e-04 | 6803862 | 6803862 | 5503804 (3.53e-04) | | 1160. | Pkp4 | P < 3.539e-04 | 6877428 | 6877428 | 4737468 (3.54e-04) | | 1161. | Ly75 | P < 3.542e-04 | 6887081 | 6887081 | 4913910 (3.54e-04) | | 1162. | Hsbp1 | P < 3.546e-04 | 6979493 | 6979493 | 5065337 (3.55e-04) | | 1163. | Fdxr | P < 3.548e-04 | 6792419 | 6792419 | 5159792 (3.55e-04) | | 1164. | NA | P < 3.549e-04 | 6987446 | 6987446 | 5197750 (3.55e-04) | | 1165. | Atp8a1 | P < 3.552e-04 | 6938891 | 6938891 | 4752831 (3.55e-04) | 4594764 (4.34e-04) | | 1166. | Mgat1 | P < 3.559e-04 | 6780725 | 6780725 | 4944479 (3.56e-04) | | 1167. | Ablim1 | P < 3.562e-04 | 6874001 | 6874001 | 4672934 (3.56e-04) | 5589674 (1.05e-03) | | 1168. | Ptges2 | P < 3.591e-04 | 6876188 | 6876188 | 4852506 (3.59e-04) | | 1169. | Mc1r | P < 3.614e-04 | 6979704 | 6979704 | 4591149 (3.61e-04) | 4630035 (6.14e-04) | | 1170. | Lrpap1 | P < 3.615e-04 | 6937364 | 6937364 | 5237550 (3.62e-04) | | 1171. | Trub1 | P < 3.618e-04 | 6870680 | 6870680 | 4345088 (3.62e-04) | | 1172. | Snx2 | P < 3.628e-04 | 6860925 | 6860925 | 4530722 (3.63e-04) | | 1173. | Fpgs | P < 3.635e-04 | 6885901 | 6885901 | 5445390 (3.64e-04) | | 1174. | Rab30 | P < 3.655e-04 | 6962577 | 6962577 | 5612850 (3.66e-04) | | 1175. | Osbpl1a | P < 3.686e-04 | 6863467 | 6863467 | 4964465 (3.69e-04) | | 1176. | A930016P21Rik | P < 3.686e-04 | 6829551 | 6829551 | 4759237 (3.69e-04) | | 1177. | P4hb | P < 3.687e-04 | 6792779 | 6792779 | 4563328 (3.69e-04) | | 1178. | NA | P < 3.697e-04 | 6815491 | 6815491 | 5471612 (3.70e-04) | | 1179. | Pdlim2 | P < 3.698e-04 | 6825683 | 6825683 | 4596496 (3.70e-04) | 4872297 (9.81e-04) | 4673532 (1.05e-03) | | 1180. | Mlph | P < 3.703e-04 | 6751520 | 6751520 | 4657656 (3.70e-04) | | 1181. | Gtpbp3 | P < 3.715e-04 | 6977078 | 6977078 | 4821561 (3.72e-04) | | 1182. | 1200009I06Rik | P < 3.722e-04 | 6798212 | 6798212 | 4324320 (3.72e-04) | 4851920 (7.91e-04) | | 1183. | 1500011H22Rik | P < 3.723e-04 | 6941739 | 6941739 | 5116181 (3.72e-04) | | 1184. | Mrc2 | P < 3.737e-04 | 6784564 | 6784564 | 5497290 (3.74e-04) | | 1185. | Gtse1 | P < 3.745e-04 | 6832394 | 6832394 | 4761644 (3.75e-04) | | 1186. | Fermt3 | P < 3.753e-04 | 6871276 | 6871276 | 5228857 (3.75e-04) | | 1187. | Eif2a | P < 3.757e-04 | 6897762 | 6897762 | 4313050 (3.76e-04) | | 1188. | Pqlc2 | P < 3.757e-04 | 6926341 | 6926341 | 5106619 (3.76e-04) | | 1189. | Nhlh2 | P < 3.764e-04 | 6900044 | 6900044 | 5450981 (3.76e-04) | | 1190. | Adam19 | P < 3.767e-04 | 6780527 | 6780527 | 4680440 (3.77e-04) | | 1191. | Wnt7b | P < 3.774e-04 | 6837582 | 6837582 | 4314431 (3.77e-04) | | 1192. | Oplah | P < 3.775e-04 | 6836810 | 6836810 | 4917870 (3.78e-04) | | 1193. | Reln | P < 3.777e-04 | 6936589 | 6936589 | 5140733 (3.78e-04) | | 1194. | Nipbl | P < 3.788e-04 | 6833842 | 6833842 | 5468248 (3.79e-04) | | 1195. | Gpx1 | P < 3.791e-04 | 6992332 | 6992332 | 4910569 (3.79e-04) | | 1196. | Hdac11 | P < 3.798e-04 | 6947985 | 6947985 | 5215729 (3.80e-04) | 5431606 (8.69e-04) | | 1197. | Eltd1 | P < 3.805e-04 | 6902440 | 6902440 | 4556843 (3.81e-04) | | 1198. | NA | P < 3.812e-04 | 6848568 | 6848568 | 5145642 (3.81e-04) | | 1199. | Bcl11a | P < 3.822e-04 | 6779432 | 6779432 | 5381910 (3.82e-04) | 5257645 (6.17e-04) | | 1200. | Tbc1d23 | P < 3.823e-04 | 6846457 | 6846457 | 5265311 (3.82e-04) | | 1201. | Tpr | P < 3.838e-04 | 6753937 | 6753937 | 4386056 (3.84e-04) | | 1202. | Wdr74 | P < 3.858e-04 | 6867955 | 6867955 | 5089432 (3.86e-04) | | 1203. | Mcm4 | P < 3.865e-04 | 6844177 | 6844177 | 5510267 (3.87e-04) | | 1204. | Sgol2 | P < 3.865e-04 | 6749701 | 6749701 | 4856275 (3.87e-04) | | 1205. | St6gal1 | P < 3.875e-04 | 6840115 | 6840115 | 5111254 (3.87e-04) | | 1206. | Ccdc33 | P < 3.891e-04 | 6995950 | 6995950 | 4838374 (3.89e-04) | 4837025 (7.79e-04) | | 1207. | Rbl1 | P < 3.892e-04 | 6892505 | 6892505 | 4544330 (3.89e-04) | 5530875 (4.33e-04) | | 1208. | 1700021F05Rik | P < 3.915e-04 | 6773622 | 6773622 | 4810512 (3.91e-04) | | 1209. | Gad1 | P < 3.939e-04 | 6877963 | 6877963 | 4451922 (3.94e-04) | | 1210. | Sart1 | P < 3.943e-04 | 6871102 | 6871102 | 4517794 (3.94e-04) | | 1211. | Akr1e1 | P < 3.946e-04 | 6810717 | 6810717 | 4985008 (3.95e-04) | | 1212. | Spen | P < 3.951e-04 | 6926447 | 6926447 | 4772721 (3.95e-04) | 5614829 (9.39e-04) | | 1213. | Atf3 | P < 3.951e-04 | 6765218 | 6765218 | 4948304 (3.95e-04) | | 1214. | Psmb10 | P < 3.966e-04 | 6985004 | 6985004 | 4595633 (3.97e-04) | | 1215. | Setbp1 | P < 3.968e-04 | 6866862 | 6866862 | 4490138 (3.97e-04) | 4512987 (7.10e-04) | | 1216. | Creld1 | P < 3.991e-04 | 6949095 | 6949095 | 5312941 (3.99e-04) | | 1217. | Lrp1 | P < 4.019e-04 | 6777957 | 6777957 | 4880224 (4.02e-04) | 4698110 (7.30e-04) | | 1218. | Prnp | P < 4.021e-04 | 6881291 | 6881291 | 5504793 (4.02e-04) | 5566985 (4.53e-04) | | 1219. | Popdc2 | P < 4.023e-04 | 6840841 | 6840841 | 5300814 (4.02e-04) | | 1220. | Kif3c | P < 4.030e-04 | 6792931 | 6792931 | 5000252 (4.03e-04) | | 1221. | Hsf1 | P < 4.033e-04 | 6831660 | 6831660 | 4786286 (4.03e-04) | | 1222. | Mink1 | P < 4.040e-04 | 6782136 | 6782136 | 4964423 (4.04e-04) | | 1223. | Atp11a | P < 4.044e-04 | 6974113 | 6974113 | 4926910 (4.04e-04) | | 1224. | Akap7 | P < 4.045e-04 | 6772829 | 6772829 | 4465636 (4.04e-04) | | 1225. | Camk1 | P < 4.053e-04 | 6956550 | 6956550 | 5593541 (4.05e-04) | | 1226. | Tgfb1i1 | P < 4.055e-04 | 6964394 | 6964394 | 4495329 (4.05e-04) | | 1227. | Rai14 | P < 4.059e-04 | 6833980 | 6833980 | 4456110 (4.06e-04) | | 1228. | Polq | P < 4.065e-04 | 6840782 | 6840782 | 5598582 (4.07e-04) | | 1229. | Ankrd27 | P < 4.067e-04 | 6959817 | 6959817 | 4374584 (4.07e-04) | 4950163 (9.70e-04) | | 1230. | Samd8 | P < 4.068e-04 | 6817412 | 6817412 | 4552541 (4.07e-04) | | 1231. | Zer1 | P < 4.072e-04 | 6885734 | 6885734 | 5262272 (4.07e-04) | | 1232. | Glt8d4 | P < 4.086e-04 | 6948649 | 6948649 | 5456693 (4.09e-04) | 4843772 (1.06e-03) | | 1233. | E130203B14Rik | P < 4.093e-04 | 6818104 | 6818104 | 4583417 (4.09e-04) | | 1234. | Apbb2 | P < 4.093e-04 | 6938797 | 6938797 | 4827469 (4.09e-04) | 4734027 (7.96e-04) | | 1235. | Fosl2 | P < 4.103e-04 | 6929719 | 6929719 | 4584829 (4.10e-04) | | 1236. | NA | P < 4.105e-04 | 6942376 | 6942376 | 5235131 (4.11e-04) | | 1237. | Apbb1 | P < 4.113e-04 | 6970134 | 6970134 | 4858800 (4.11e-04) | | 1238. | Hspa9 | P < 4.117e-04 | 6864565 | 6864565 | 4483406 (4.12e-04) | | 1239. | Srm | P < 4.118e-04 | 6918720 | 6918720 | 4404953 (4.12e-04) | | 1240. | Top1 | P < 4.120e-04 | 6882878 | 6882878 | 4698860 (4.12e-04) | | 1241. | Kif21a | P < 4.123e-04 | 6837925 | 6837925 | 4479698 (4.12e-04) | | 1242. | 2310079F23Rik | P < 4.123e-04 | 6937433 | 6937433 | 5210259 (4.12e-04) | | 1243. | Pafah1b3 | P < 4.129e-04 | 6965940 | 6965940 | 4386116 (4.13e-04) | 4632102 (4.46e-04) | | 1244. | Higd1a | P < 4.131e-04 | 6999549 | 6999549 | 4719237 (4.13e-04) | | 1245. | Nol6 | P < 4.135e-04 | 6921015 | 6921015 | 5321865 (4.13e-04) | | 1246. | L3mbtl2 | P < 4.135e-04 | 6832117 | 6832117 | 4536233 (4.14e-04) | 4467030 (9.39e-04) | | 1247. | Drp2 | P < 4.143e-04 | 7013867 | 7013867 | 5238133 (4.14e-04) | | 1248. | Fancg | P < 4.153e-04 | 6921127 | 6921127 | 4879764 (4.15e-04) | | 1249. | Mkx | P < 4.168e-04 | 6863210 | 6863210 | 5020464 (4.17e-04) | | 1250. | Dhx29 | P < 4.169e-04 | 6810317 | 6810317 | 4320485 (4.17e-04) | | 1251. | Sash3 | P < 4.171e-04 | 7011035 | 7011035 | 5403051 (4.17e-04) | | 1252. | Gabrb2 | P < 4.180e-04 | 6780331 | 6780331 | 5486573 (4.18e-04) | | 1253. | Ccnc | P < 4.186e-04 | 6912092 | 6912092 | 4923568 (4.19e-04) | | 1254. | Sys1 | P < 4.187e-04 | 6883097 | 6883097 | 4823160 (4.19e-04) | | 1255. | Rabgap1 | P < 4.193e-04 | 6876486 | 6876486 | 5387317 (4.19e-04) | | 1256. | Pknox2 | P < 4.195e-04 | 6994672 | 6994672 | 4758482 (4.20e-04) | | 1257. | Cdv3 | P < 4.204e-04 | 6998401 | 6998401 | 4387911 (4.20e-04) | | 1258. | Ddx59 | P < 4.211e-04 | 6753430 | 6753430 | 4894924 (4.21e-04) | | 1259. | NA | P < 4.213e-04 | 6757979 | 6757979 | 5513331 (4.21e-04) | | 1260. | Irgm | P < 4.221e-04 | 6787896 | 6787896 | 5249908 (4.22e-04) | | 1261. | Plch2 | P < 4.228e-04 | 6927238 | 6927238 | 4441796 (4.23e-04) | | 1262. | 2310044H10Rik | P < 4.257e-04 | 6966869 | 6966869 | 4660693 (4.26e-04) | | 1263. | Oxnad1 | P < 4.264e-04 | 6818053 | 6818053 | 4615906 (4.26e-04) | | 1264. | Fbxl19 | P < 4.272e-04 | 6964345 | 6964345 | 5300312 (4.27e-04) | | 1265. | Thoc4 | P < 4.273e-04 | 6792784 | 6792784 | 5199587 (4.27e-04) | | 1266. | Col3a1 | P < 4.280e-04 | 6749142 | 6749142 | 5480837 (4.28e-04) | 5278479 (1.03e-03) | | 1267. | Spnb3 | P < 4.286e-04 | 6867685 | 6867685 | 5432759 (4.29e-04) | 4438430 (8.33e-04) | 4525297 (9.67e-04) | | 1268. | Ankrd56 | P < 4.297e-04 | 6940039 | 6940039 | 5342265 (4.30e-04) | | 1269. | Usp5 | P < 4.302e-04 | 6957134 | 6957134 | 5257802 (4.30e-04) | | 1270. | Cplx2 | P < 4.304e-04 | 6807158 | 6807158 | 4706881 (4.30e-04) | | 1271. | Wdr12 | P < 4.313e-04 | 6759043 | 6759043 | 4652274 (4.31e-04) | | 1272. | Cpe | P < 4.324e-04 | 6982918 | 6982918 | 5212227 (4.32e-04) | | 1273. | Col27a1 | P < 4.336e-04 | 6914006 | 6914006 | 4458913 (4.34e-04) | | 1274. | Azi1 | P < 4.341e-04 | 6792738 | 6792738 | 5207010 (4.34e-04) | 4908358 (6.71e-04) | | 1275. | Wdr26 | P < 4.343e-04 | 6764662 | 6764662 | 5558957 (4.34e-04) | | 1276. | Cops3 | P < 4.343e-04 | 6788702 | 6788702 | 4697443 (4.34e-04) | | 1277. | Med15 | P < 4.347e-04 | 6844291 | 6844291 | 5452691 (4.35e-04) | | 1278. | Rec8 | P < 4.348e-04 | 6819259 | 6819259 | 4645831 (4.35e-04) | | 1279. | Polrmt | P < 4.355e-04 | 6775309 | 6775309 | 5549764 (4.35e-04) | | 1280. | Rrp15 | P < 4.367e-04 | 6764956 | 6764956 | 4535586 (4.37e-04) | | 1281. | H6pd | P < 4.377e-04 | 6926988 | 6926988 | 4438907 (4.38e-04) | | 1282. | Card11 | P < 4.384e-04 | 6942753 | 6942753 | 4306845 (4.38e-04) | | 1283. | Dock7 | P < 4.392e-04 | 6923694 | 6923694 | 4741038 (4.39e-04) | | 1284. | Atp10a | P < 4.392e-04 | 6960915 | 6960915 | 5205417 (4.39e-04) | | 1285. | NA | P < 4.398e-04 | 6849098 | 6849098 | 4665235 (4.40e-04) | | 1286. | Asrgl1 | P < 4.404e-04 | 6871429 | 6871429 | 4952698 (4.40e-04) | | 1287. | Sh2b2 | P < 4.416e-04 | 6942494 | 6942494 | 4683642 (4.42e-04) | | 1288. | Gmeb2 | P < 4.417e-04 | 6894278 | 6894278 | 4312575 (4.42e-04) | 5234154 (6.99e-04) | | 1289. | Rfc1 | P < 4.441e-04 | 6938698 | 6938698 | 4305087 (4.44e-04) | | 1290. | Vldlr | P < 4.485e-04 | 6868899 | 6868899 | 5462256 (4.49e-04) | | 1291. | Pik3cd | P < 4.490e-04 | 6926974 | 6926974 | 4982634 (4.49e-04) | | 1292. | Gripap1 | P < 4.511e-04 | 7009788 | 7009788 | 5297658 (4.51e-04) | | 1293. | Macrod1 | P < 4.534e-04 | 6867885 | 6867885 | 5449163 (4.53e-04) | | 1294. | Pcnx | P < 4.572e-04 | 6796485 | 6796485 | 5318122 (4.57e-04) | | 1295. | Gpr123 | P < 4.576e-04 | 6965069 | 6965069 | 4814361 (4.58e-04) | | 1296. | Magi3 | P < 4.579e-04 | 6907810 | 6907810 | 5104412 (4.58e-04) | | 1297. | Ube2v2 | P < 4.581e-04 | 6844176 | 6844176 | 5117427 (4.58e-04) | | 1298. | Prpf8 | P < 4.587e-04 | 6782447 | 6782447 | 4734598 (4.59e-04) | | 1299. | Sema6a | P < 4.589e-04 | 6865267 | 6865267 | 4891000 (4.59e-04) | | 1300. | Esr1 | P < 4.590e-04 | 6771884 | 6771884 | 4483165 (4.59e-04) | | 1301. | Rangap1 | P < 4.606e-04 | 6837301 | 6837301 | 5209603 (4.61e-04) | | 1302. | Tead2 | P < 4.624e-04 | 6960351 | 6960351 | 5055369 (4.62e-04) | | 1303. | Cdc2a | P < 4.629e-04 | 6774794 | 6774794 | 5275259 (4.63e-04) | | 1304. | Itfg3 | P < 4.631e-04 | 6854468 | 6854468 | 4833185 (4.63e-04) | | 1305. | Inpp5d | P < 4.648e-04 | 6751344 | 6751344 | 4660735 (4.65e-04) | | 1306. | Xpa | P < 4.659e-04 | 6921353 | 6921353 | 4358793 (4.66e-04) | | 1307. | Ptges | P < 4.666e-04 | 6885796 | 6885796 | 4411052 (4.67e-04) | | 1308. | Serhl | P < 4.666e-04 | 6832217 | 6832217 | 5399019 (4.67e-04) | 4882673 (7.31e-04) | 4774463 (9.76e-04) | | 1309. | Nfx1 | P < 4.687e-04 | 6912887 | 6912887 | 4958835 (4.69e-04) | | 1310. | Syn1 | P < 4.695e-04 | 7015988 | 7015988 | 5006084 (4.69e-04) | 5354090 (8.08e-04) | | 1311. | Smn1 | P < 4.695e-04 | 6809524 | 6809524 | 4344871 (4.70e-04) | | 1312. | Trim2 | P < 4.712e-04 | 6906441 | 6906441 | 4774082 (4.71e-04) | | 1313. | Fbn1 | P < 4.715e-04 | 6890597 | 6890597 | 4788336 (4.71e-04) | 5608592 (1.04e-03) | | 1314. | Nup107 | P < 4.722e-04 | 6777353 | 6777353 | 4716458 (4.72e-04) | | 1315. | A930001N09Rik | P < 4.743e-04 | 6849466 | 6849466 | 5277976 (4.74e-04) | | 1316. | C76566 | P < 4.753e-04 | 6984952 | 6984952 | 5050625 (4.75e-04) | | 1317. | Eef1d | P < 4.765e-04 | 6836779 | 6836779 | 4986283 (4.77e-04) | | 1318. | Rap2c | P < 4.768e-04 | 7016797 | 7016797 | 5252219 (4.77e-04) | | 1319. | Kpna3 | P < 4.777e-04 | 6825216 | 6825216 | 4850157 (4.78e-04) | | 1320. | Ghitm | P < 4.789e-04 | 6824007 | 6824007 | 5239225 (4.79e-04) | | 1321. | Nod1 | P < 4.796e-04 | 6953800 | 6953800 | 4666282 (4.80e-04) | | 1322. | Centb5 | P < 4.807e-04 | 6919195 | 6919195 | 5288680 (4.81e-04) | | 1323. | Ppp4c | P < 4.816e-04 | 6971291 | 6971291 | 5136736 (4.82e-04) | | 1324. | Cux2 | P < 4.824e-04 | 6941710 | 6941710 | 4986624 (4.82e-04) | 4980119 (1.03e-03) | | 1325. | Dock9 | P < 4.825e-04 | 6827925 | 6827925 | 4311806 (4.83e-04) | | 1326. | Atp8a2 | P < 4.826e-04 | 6825132 | 6825132 | 5215642 (4.83e-04) | | 1327. | Myo5a | P < 4.832e-04 | 6990527 | 6990527 | 4346845 (4.83e-04) | | 1328. | NA | P < 4.837e-04 | 6759718 | 6759718 | 4699026 (4.84e-04) | 5230070 (7.89e-04) | | 1329. | 2300002D11Rik | P < 4.843e-04 | 6925904 | 6925904 | 5613716 (4.84e-04) | | 1330. | Arid2 | P < 4.844e-04 | 6832947 | 6832947 | 5387879 (4.84e-04) | | 1331. | Mecp2 | P < 4.861e-04 | 7017610 | 7017610 | 5441830 (4.86e-04) | 5373583 (8.91e-04) | | 1332. | Psme3 | P < 4.872e-04 | 6784257 | 6784257 | 4874050 (4.87e-04) | | 1333. | Tulp2 | P < 4.882e-04 | 6960378 | 6960378 | 4325563 (4.88e-04) | | 1334. | Zfp148 | P < 4.884e-04 | 6840618 | 6840618 | 5359757 (4.88e-04) | | 1335. | Slc25a36 | P < 4.894e-04 | 6998101 | 6998101 | 5005652 (4.89e-04) | | 1336. | Gata6 | P < 4.925e-04 | 6858842 | 6858842 | 4619561 (4.93e-04) | | 1337. | Dzip1l | P < 4.930e-04 | 6991799 | 6991799 | 5336065 (4.93e-04) | | 1338. | NA | P < 4.946e-04 | 6939320 | 6939320 | 4497804 (4.95e-04) | | 1339. | Nlgn2 | P < 4.946e-04 | 6789351 | 6789351 | 5431189 (4.95e-04) | | 1340. | Tjp2 | P < 4.959e-04 | 6872250 | 6872250 | 4876998 (4.96e-04) | | 1341. | Tsc22d3 | P < 4.965e-04 | 7019818 | 7019818 | 4326752 (4.96e-04) | | 1342. | Pan3 | P < 4.969e-04 | 6935594 | 6935594 | 4652111 (4.97e-04) | | 1343. | Neurog2 | P < 4.972e-04 | 6901190 | 6901190 | 4599344 (4.97e-04) | 5365089 (8.04e-04) | | 1344. | Fyco1 | P < 4.979e-04 | 6999682 | 6999682 | 4540244 (4.98e-04) | | 1345. | Btbd10 | P < 4.980e-04 | 6970568 | 6970568 | 5205542 (4.98e-04) | | 1346. | Lad1 | P < 5.007e-04 | 6753400 | 6753400 | 5291461 (5.01e-04) | | 1347. | NA | P < 5.010e-04 | 6838750 | 6838750 | 5572049 (5.01e-04) | | 1348. | Eda2r | P < 5.017e-04 | 7018366 | 7018366 | 4848507 (5.02e-04) | | 1349. | Hmgn3 | P < 5.034e-04 | 6997349 | 6997349 | 5048153 (5.03e-04) | | 1350. | Ylpm1 | P < 5.062e-04 | 6796645 | 6796645 | 5052061 (5.06e-04) | | 1351. | Rg9mtd3 | P < 5.062e-04 | 6913143 | 6913143 | 4542439 (5.06e-04) | | 1352. | Per3 | P < 5.068e-04 | 6927085 | 6927085 | 4974767 (5.07e-04) | | 1353. | Cacybp | P < 5.071e-04 | 6763493 | 6763493 | 4519973 (5.07e-04) | 5453029 (6.99e-04) | | 1354. | Greb1 | P < 5.081e-04 | 6799206 | 6799206 | 5537559 (5.08e-04) | | 1355. | Pcbp3 | P < 5.087e-04 | 6775192 | 6775192 | 4340353 (5.09e-04) | 4504386 (5.54e-04) | | 1356. | Slc39a14 | P < 5.090e-04 | 6825688 | 6825688 | 5267655 (5.09e-04) | 5497317 (6.69e-04) | | 1357. | Jarid1c | P < 5.096e-04 | 7014561 | 7014561 | 4640521 (5.10e-04) | | 1358. | Papss1 | P < 5.097e-04 | 6901413 | 6901413 | 4766207 (5.10e-04) | | 1359. | Tapbpl | P < 5.116e-04 | 6957168 | 6957168 | 4621132 (5.12e-04) | | 1360. | Cadm4 | P < 5.124e-04 | 6959127 | 6959127 | 4558425 (5.12e-04) | | 1361. | Tmie | P < 5.149e-04 | 6998893 | 6998893 | 4837177 (5.15e-04) | | 1362. | Gigyf2 | P < 5.168e-04 | 6751338 | 6751338 | 4998874 (5.17e-04) | 5606805 (5.87e-04) | | 1363. | Csk | P < 5.181e-04 | 6995922 | 6995922 | 5440551 (5.18e-04) | | 1364. | Stk32c | P < 5.225e-04 | 6971996 | 6971996 | 5563264 (5.23e-04) | | 1365. | Phc1 | P < 5.249e-04 | 6957030 | 6957030 | 4860483 (5.25e-04) | | 1366. | Usp3 | P < 5.291e-04 | 6996391 | 6996391 | 5572986 (5.29e-04) | | 1367. | Atp2a2 | P < 5.297e-04 | 6941751 | 6941751 | 4418573 (5.30e-04) | | 1368. | Gnao1 | P < 5.298e-04 | 6978263 | 6978263 | 4680958 (5.30e-04) | | 1369. | Samhd1 | P < 5.298e-04 | 6892504 | 6892504 | 4319771 (5.30e-04) | | 1370. | Zfr | P < 5.311e-04 | 6828790 | 6828790 | 5552856 (5.31e-04) | | 1371. | Grcc10 | P < 5.316e-04 | 6957123 | 6957123 | 4394570 (5.32e-04) | | 1372. | Atg4b | P < 5.348e-04 | 6751712 | 6751712 | 4434471 (5.35e-04) | | 1373. | 8430419L09Rik | P < 5.378e-04 | 6950397 | 6950397 | 5009696 (5.38e-04) | 4650772 (7.68e-04) | | 1374. | Smg7 | P < 5.394e-04 | 6763110 | 6763110 | 4313835 (5.39e-04) | | 1375. | Khsrp | P < 5.415e-04 | 6856270 | 6856270 | 4443237 (5.42e-04) | 5332952 (6.04e-04) | | 1376. | Abca1 | P < 5.432e-04 | 6921670 | 6921670 | 5290755 (5.43e-04) | 4689212 (7.88e-04) | | 1377. | Caskin1 | P < 5.432e-04 | 6849297 | 6849297 | 4953457 (5.43e-04) | | 1378. | Mcm3ap | P < 5.434e-04 | 6768926 | 6768926 | 5169198 (5.43e-04) | | 1379. | Cnot6 | P < 5.447e-04 | 6787967 | 6787967 | 5450708 (5.45e-04) | | 1380. | Camk4 | P < 5.486e-04 | 6859850 | 6859850 | 5351530 (5.49e-04) | | 1381. | BC018242 | P < 5.492e-04 | 6987414 | 6987414 | 4874806 (5.49e-04) | | 1382. | Chmp5 | P < 5.495e-04 | 6912885 | 6912885 | 5085611 (5.50e-04) | | 1383. | Wdr77 | P < 5.498e-04 | 6900236 | 6900236 | 5568052 (5.50e-04) | | 1384. | Crebl2 | P < 5.501e-04 | 6950384 | 6950384 | 4729451 (5.50e-04) | | 1385. | Rasa2 | P < 5.508e-04 | 6998076 | 6998076 | 4308945 (5.51e-04) | | 1386. | Parp11 | P < 5.523e-04 | 6949992 | 6949992 | 5257694 (5.52e-04) | | 1387. | Bcl11b | P < 5.527e-04 | 6803524 | 6803524 | 4391059 (5.53e-04) | | 1388. | Gsr | P < 5.546e-04 | 6975235 | 6975235 | 4580304 (5.55e-04) | 5129794 (9.98e-04) | | 1389. | Psmc3 | P < 5.547e-04 | 6879016 | 6879016 | 4508297 (5.55e-04) | | 1390. | Usp7 | P < 5.552e-04 | 6843834 | 6843834 | 5027757 (5.55e-04) | | 1391. | Muted | P < 5.576e-04 | 6812516 | 6812516 | 4889341 (5.58e-04) | | 1392. | Ikbkg | P < 5.577e-04 | 7012015 | 7012015 | 4536196 (5.58e-04) | | 1393. | Dync2li1 | P < 5.581e-04 | 6852751 | 6852751 | 4703808 (5.58e-04) | | 1394. | Ednra | P < 5.591e-04 | 6983531 | 6983531 | 5206092 (5.59e-04) | | 1395. | Dlgap3 | P < 5.592e-04 | 6917217 | 6917217 | 4551149 (5.59e-04) | | 1396. | Disc1 | P < 5.607e-04 | 6979856 | 6979856 | 4704135 (5.61e-04) | | 1397. | Rnase4 | P < 5.615e-04 | 6818956 | 6818956 | 4471914 (5.61e-04) | 4455871 (6.03e-04) | | 1398. | Ryr3 | P < 5.616e-04 | 6889777 | 6889777 | 4433473 (5.62e-04) | | 1399. | Ppie | P < 5.629e-04 | 6925156 | 6925156 | 4366293 (5.63e-04) | | 1400. | Rasgrp1 | P < 5.643e-04 | 6890068 | 6890068 | 5193642 (5.64e-04) | | 1401. | Tnmd | P < 5.664e-04 | 7013835 | 7013835 | 4346656 (5.66e-04) | | 1402. | Col1a1 | P < 5.683e-04 | 6783685 | 6783685 | 4731409 (5.68e-04) | | 1403. | NA | P < 5.700e-04 | 6881181 | 6881181 | 5173762 (5.70e-04) | 4832014 (7.15e-04) | 4624953 (7.84e-04) | | 1404. | Smarcc2 | P < 5.701e-04 | 6771662 | 6771662 | 5278041 (5.70e-04) | | 1405. | Rabep1 | P < 5.717e-04 | 6782164 | 6782164 | 4331173 (5.72e-04) | | 1406. | Frmd4b | P < 5.719e-04 | 6955778 | 6955778 | 5063687 (5.72e-04) | | 1407. | Shroom3 | P < 5.737e-04 | 6932510 | 6932510 | 5270732 (5.74e-04) | | 1408. | Krt80 | P < 5.754e-04 | 6838621 | 6838621 | 4433540 (5.75e-04) | | 1409. | Tsga14 | P < 5.768e-04 | 6952436 | 6952436 | 4391689 (5.77e-04) | | 1410. | Lrrc45 | P < 5.770e-04 | 6785481 | 6785481 | 4410705 (5.77e-04) | | 1411. | Tdrkh | P < 5.800e-04 | 6899578 | 6899578 | 4942414 (5.80e-04) | | 1412. | Ace | P < 5.805e-04 | 6784587 | 6784587 | 4801085 (5.80e-04) | | 1413. | Canx | P < 5.824e-04 | 6788020 | 6788020 | 4728482 (5.82e-04) | | 1414. | Pon3 | P < 5.834e-04 | 6951401 | 6951401 | 5168463 (5.83e-04) | | 1415. | Ltbp3 | P < 5.834e-04 | 6867785 | 6867785 | 4339112 (5.83e-04) | 4314104 (6.82e-04) | | 1416. | Dync1h1 | P < 5.836e-04 | 6798108 | 6798108 | 4413948 (5.84e-04) | | 1417. | Adh5 | P < 5.845e-04 | 6901747 | 6901747 | 4937735 (5.85e-04) | | 1418. | Wdr48 | P < 5.866e-04 | 6992898 | 6992898 | 4568851 (5.87e-04) | | 1419. | Jmjd1a | P < 5.867e-04 | 6954572 | 6954572 | 5101094 (5.87e-04) | | 1420. | Hdlbp | P < 5.889e-04 | 6760777 | 6760777 | 5532205 (5.89e-04) | | 1421. | Zfp704 | P < 5.913e-04 | 6903129 | 6903129 | 5381996 (5.91e-04) | | 1422. | Fancl | P < 5.914e-04 | 6779588 | 6779588 | 5482720 (5.91e-04) | | 1423. | Lyst | P < 5.932e-04 | 6804898 | 6804898 | 4698006 (5.93e-04) | | 1424. | Bcl7c | P < 5.935e-04 | 6971393 | 6971393 | 4573497 (5.94e-04) | | 1425. | Tgfb2 | P < 5.953e-04 | 6764953 | 6764953 | 5396639 (5.95e-04) | | 1426. | Ngly1 | P < 5.953e-04 | 6817136 | 6817136 | 5332465 (5.95e-04) | | 1427. | Phf1 | P < 5.963e-04 | 6849474 | 6849474 | 5433382 (5.96e-04) | | 1428. | Afap1 | P < 5.974e-04 | 6929920 | 6929920 | 4913064 (5.97e-04) | | 1429. | Sgsm1 | P < 5.978e-04 | 6941157 | 6941157 | 4684091 (5.98e-04) | | 1430. | Gsk3b | P < 5.982e-04 | 6840828 | 6840828 | 5014484 (5.98e-04) | | 1431. | Stap2 | P < 5.982e-04 | 6856192 | 6856192 | 4480085 (5.98e-04) | | 1432. | Fgd5 | P < 6.003e-04 | 6948034 | 6948034 | 5551976 (6.00e-04) | | 1433. | Rab21 | P < 6.004e-04 | 6777180 | 6777180 | 5600183 (6.00e-04) | | 1434. | Ccdc85a | P < 6.006e-04 | 6786914 | 6786914 | 4383470 (6.01e-04) | | 1435. | Psap | P < 6.021e-04 | 6768155 | 6768155 | 4938490 (6.02e-04) | | 1436. | Tada2l | P < 6.024e-04 | 6790318 | 6790318 | 5532758 (6.02e-04) | 4390515 (6.90e-04) | | 1437. | Dlx2 | P < 6.025e-04 | 6887715 | 6887715 | 5354902 (6.02e-04) | 4966005 (7.24e-04) | | 1438. | Cerk | P < 6.028e-04 | 6837614 | 6837614 | 4950794 (6.03e-04) | | 1439. | NA | P < 6.038e-04 | 6921057 | 6921057 | 4548773 (6.04e-04) | | 1440. | Galnt12 | P < 6.060e-04 | 6913262 | 6913262 | 5310975 (6.06e-04) | | 1441. | Agtpbp1 | P < 6.076e-04 | 6813664 | 6813664 | 5369169 (6.08e-04) | | 1442. | Ehmt2 | P < 6.082e-04 | 6850074 | 6850074 | 5593763 (6.08e-04) | | 1443. | Capn2 | P < 6.082e-04 | 6764721 | 6764721 | 4930745 (6.08e-04) | | 1444. | Atic | P < 6.088e-04 | 6750351 | 6750351 | 5441164 (6.09e-04) | | 1445. | Fli1 | P < 6.103e-04 | 6994431 | 6994431 | 4433838 (6.10e-04) | 4887388 (6.65e-04) | | 1446. | Slc39a7 | P < 6.134e-04 | 6855003 | 6855003 | 5435979 (6.13e-04) | | 1447. | Lhx8 | P < 6.135e-04 | 6910727 | 6910727 | 4571238 (6.13e-04) | | 1448. | Eapp | P < 6.141e-04 | 6800890 | 6800890 | 4621653 (6.14e-04) | | 1449. | Trap1 | P < 6.156e-04 | 6843629 | 6843629 | 4427378 (6.16e-04) | | 1450. | Rps19bp1 | P < 6.161e-04 | 6837189 | 6837189 | 5111120 (6.16e-04) | | 1451. | Atp4a | P < 6.176e-04 | 6959615 | 6959615 | 4545163 (6.18e-04) | | 1452. | Ephx1 | P < 6.183e-04 | 6764650 | 6764650 | 4427878 (6.18e-04) | | 1453. | Timp2 | P < 6.204e-04 | 6792649 | 6792649 | 5243066 (6.20e-04) | | 1454. | Raet1a | P < 6.215e-04 | 6766470 | 6766470 | 5149760 (6.21e-04) | | 1455. | St5 | P < 6.253e-04 | 6970378 | 6970378 | 4974158 (6.25e-04) | | 1456. | Osbpl6 | P < 6.258e-04 | 6878344 | 6878344 | 4382997 (6.26e-04) | | 1457. | Scarf2 | P < 6.262e-04 | 6839771 | 6839771 | 5294660 (6.26e-04) | | 1458. | Egfr | P < 6.279e-04 | 6778972 | 6778972 | 5316652 (6.28e-04) | | 1459. | Nup93 | P < 6.294e-04 | 6978296 | 6978296 | 4346501 (6.29e-04) | | 1460. | Lbxcor1 | P < 6.296e-04 | 6996246 | 6996246 | 4715577 (6.30e-04) | | 1461. | Ids | P < 6.302e-04 | 7017419 | 7017419 | 4861104 (6.30e-04) | | 1462. | Myh10 | P < 6.304e-04 | 6781941 | 6781941 | 4862698 (6.30e-04) | | 1463. | Zfp275 | P < 6.308e-04 | 7011928 | 7011928 | 4911948 (6.31e-04) | | 1464. | Chd1 | P < 6.320e-04 | 6848877 | 6848877 | 4581682 (6.32e-04) | | 1465. | Zic4 | P < 6.324e-04 | 6991326 | 6991326 | 4921704 (6.32e-04) | 5022237 (9.82e-04) | | 1466. | Atrnl1 | P < 6.327e-04 | 6870697 | 6870697 | 4513288 (6.33e-04) | 5523904 (8.94e-04) | | 1467. | Aff1 | P < 6.330e-04 | 6933028 | 6933028 | 4856020 (6.33e-04) | | 1468. | Tnfaip1 | P < 6.334e-04 | 6789974 | 6789974 | 4528743 (6.33e-04) | | 1469. | Arl4a | P < 6.349e-04 | 6800228 | 6800228 | 4820630 (6.35e-04) | | 1470. | Plekhg3 | P < 6.351e-04 | 6796193 | 6796193 | 4526254 (6.35e-04) | | 1471. | Brap | P < 6.353e-04 | 6934076 | 6934076 | 5450535 (6.35e-04) | | 1472. | Lphn1 | P < 6.375e-04 | 6977692 | 6977692 | 4840964 (6.37e-04) | | 1473. | Tmem2 | P < 6.387e-04 | 6868650 | 6868650 | 4557494 (6.39e-04) | | 1474. | Tle1 | P < 6.393e-04 | 6922573 | 6922573 | 4736343 (6.39e-04) | | 1475. | Dscam | P < 6.395e-04 | 6848179 | 6848179 | 5599145 (6.40e-04) | | 1476. | Mbtps1 | P < 6.434e-04 | 6985804 | 6985804 | 5427887 (6.43e-04) | 5421582 (9.62e-04) | | 1477. | Acadvl | P < 6.448e-04 | 6789378 | 6789378 | 4551720 (6.45e-04) | 5180343 (9.62e-04) | | 1478. | Glp1r | P < 6.460e-04 | 6849762 | 6849762 | 4547912 (6.46e-04) | | 1479. | Ktn1 | P < 6.539e-04 | 6818722 | 6818722 | 5355410 (6.54e-04) | | 1480. | Ephb1 | P < 6.540e-04 | 6998327 | 6998327 | 5248464 (6.54e-04) | | 1481. | 2700094K13Rik | P < 6.561e-04 | 6888309 | 6888309 | 5550028 (6.56e-04) | | 1482. | Tef | P < 6.568e-04 | 6832132 | 6832132 | 5208302 (6.57e-04) | | 1483. | Fndc3b | P < 6.571e-04 | 6903779 | 6903779 | 4551214 (6.57e-04) | 4630860 (6.74e-04) | | 1484. | NA | P < 6.580e-04 | 6965348 | 6965348 | 4640376 (6.58e-04) | | 1485. | Centg2 | P < 6.593e-04 | 6751442 | 6751442 | 5595424 (6.59e-04) | | 1486. | Btbd12 | P < 6.603e-04 | 6843624 | 6843624 | 5340154 (6.60e-04) | | 1487. | Midn | P < 6.608e-04 | 6769193 | 6769193 | 5149913 (6.61e-04) | | 1488. | Fnbp1l | P < 6.610e-04 | 6908886 | 6908886 | 4485802 (6.61e-04) | | 1489. | Plcg2 | P < 6.611e-04 | 6979439 | 6979439 | 4893048 (6.61e-04) | | 1490. | Syt14 | P < 6.625e-04 | 6765313 | 6765313 | 5241509 (6.63e-04) | | 1491. | Smox | P < 6.643e-04 | 6881267 | 6881267 | 4918743 (6.64e-04) | | 1492. | Sh3bp5 | P < 6.646e-04 | 6823721 | 6823721 | 4902016 (6.65e-04) | | 1493. | Endod1 | P < 6.657e-04 | 6993465 | 6993465 | 4626264 (6.66e-04) | | 1494. | Atxn1 | P < 6.712e-04 | 6812894 | 6812894 | 5395822 (6.71e-04) | | 1495. | Gars | P < 6.715e-04 | 6946396 | 6946396 | 4352124 (6.71e-04) | 4684799 (1.05e-03) | | 1496. | Bax | P < 6.726e-04 | 6966985 | 6966985 | 4731712 (6.73e-04) | | 1497. | Stac2 | P < 6.730e-04 | 6791236 | 6791236 | 5335734 (6.73e-04) | | 1498. | NA | P < 6.735e-04 | 6964163 | 6964163 | 5070353 (6.73e-04) | | 1499. | Vprbp | P < 6.738e-04 | 6992189 | 6992189 | 5444677 (6.74e-04) | 4652777 (9.09e-04) | | 1500. | Rassf3 | P < 6.745e-04 | 6777583 | 6777583 | 4713866 (6.74e-04) | | 1501. | Utp14b | P < 6.746e-04 | 6750868 | 6750868 | 4481573 (6.75e-04) | | 1502. | Cmtm8 | P < 6.750e-04 | 6999154 | 6999154 | 5064705 (6.75e-04) | | 1503. | Cth | P < 6.755e-04 | 6910938 | 6910938 | 5466158 (6.76e-04) | | 1504. | Ncoa1 | P < 6.757e-04 | 6798601 | 6798601 | 4769828 (6.76e-04) | | 1505. | Rgp1 | P < 6.760e-04 | 6913013 | 6913013 | 4878608 (6.76e-04) | | 1506. | Trfr2 | P < 6.762e-04 | 6935074 | 6935074 | 4937061 (6.76e-04) | | 1507. | Slc24a3 | P < 6.767e-04 | 6881870 | 6881870 | 5170706 (6.77e-04) | | 1508. | Zfp609 | P < 6.783e-04 | 6996370 | 6996370 | 4873722 (6.78e-04) | | 1509. | Pabpc1 | P < 6.795e-04 | 6835005 | 6835005 | 4922044 (6.80e-04) | | 1510. | Lsp1 | P < 6.805e-04 | 6965268 | 6965268 | 4924274 (6.81e-04) | | 1511. | Dennd1a | P < 6.807e-04 | 6886142 | 6886142 | 5463628 (6.81e-04) | | 1512. | D2Wsu81e | P < 6.819e-04 | 6885749 | 6885749 | 5258371 (6.82e-04) | | 1513. | NA | P < 6.822e-04 | 6783137 | 6783137 | 4485599 (6.82e-04) | | 1514. | Lifr | P < 6.843e-04 | 6828522 | 6828522 | 5165283 (6.84e-04) | | 1515. | Snx14 | P < 6.843e-04 | 6997626 | 6997626 | 4711086 (6.84e-04) | | 1516. | Tbrg4 | P < 6.858e-04 | 6785808 | 6785808 | 4580468 (6.86e-04) | | 1517. | Abca2 | P < 6.868e-04 | 6875722 | 6875722 | 5105225 (6.87e-04) | 4811051 (8.89e-04) | | 1518. | Hsp90b1 | P < 6.894e-04 | 6775758 | 6775758 | 4441973 (6.89e-04) | | 1519. | Srd5a1 | P < 6.899e-04 | 6814157 | 6814157 | 4347806 (6.90e-04) | | 1520. | Armc5 | P < 6.904e-04 | 6964392 | 6964392 | 5374829 (6.90e-04) | | 1521. | Dnm1l | P < 6.907e-04 | 6844196 | 6844196 | 5512910 (6.91e-04) | | 1522. | Usp53 | P < 6.921e-04 | 6908919 | 6908919 | 4984534 (6.92e-04) | | 1523. | Elovl6 | P < 6.931e-04 | 6901316 | 6901316 | 5478036 (6.93e-04) | | 1524. | Pygb | P < 6.939e-04 | 6882181 | 6882181 | 5445979 (6.94e-04) | | 1525. | Scube2 | P < 6.959e-04 | 6970392 | 6970392 | 5121629 (6.96e-04) | | 1526. | Slc12a7 | P < 6.964e-04 | 6808221 | 6808221 | 4421406 (6.96e-04) | | 1527. | Rage | P < 6.971e-04 | 6803770 | 6803770 | 4782638 (6.97e-04) | | 1528. | Tpd52l2 | P < 6.971e-04 | 6884277 | 6884277 | 4473030 (6.97e-04) | | 1529. | Tex2 | P < 6.996e-04 | 6791942 | 6791942 | 5314283 (7.00e-04) | | 1530. | Snx5 | P < 7.013e-04 | 6891675 | 6891675 | 4434456 (7.01e-04) | | 1531. | Tmem131 | P < 7.025e-04 | 6757945 | 6757945 | 4841391 (7.03e-04) | | 1532. | Arglu1 | P < 7.030e-04 | 6980271 | 6980271 | 5139949 (7.03e-04) | | 1533. | Zfp385a | P < 7.030e-04 | 6838808 | 6838808 | 4608323 (7.03e-04) | | 1534. | Igf1 | P < 7.040e-04 | 6769597 | 6769597 | 5261482 (7.04e-04) | | 1535. | Cbara1 | P < 7.046e-04 | 6768143 | 6768143 | 5043881 (7.05e-04) | | 1536. | Fbxl11 | P < 7.048e-04 | 6870996 | 6870996 | 4638170 (7.05e-04) | | 1537. | Hyal2 | P < 7.048e-04 | 6992221 | 6992221 | 4312155 (7.05e-04) | | 1538. | Ric8 | P < 7.058e-04 | 6965130 | 6965130 | 5600283 (7.06e-04) | | 1539. | Agrn | P < 7.109e-04 | 6927362 | 6927362 | 5601266 (7.11e-04) | | 1540. | Usp25 | P < 7.124e-04 | 6842503 | 6842503 | 5272010 (7.12e-04) | | 1541. | 4932417H02Rik | P < 7.129e-04 | 6785394 | 6785394 | 4463376 (7.13e-04) | | 1542. | Zfp462 | P < 7.134e-04 | 6913619 | 6913619 | 4839440 (7.13e-04) | | 1543. | Vav3 | P < 7.147e-04 | 6900456 | 6900456 | 5588958 (7.15e-04) | | 1544. | AB030242 | P < 7.159e-04 | 6936702 | 6936702 | 5320420 (7.16e-04) | | 1545. | Ubash3b | P < 7.174e-04 | 6994887 | 6994887 | 5096915 (7.17e-04) | | 1546. | Zbtb20 | P < 7.187e-04 | 6841019 | 6841019 | 4569053 (7.19e-04) | | 1547. | Pnpla6 | P < 7.193e-04 | 6973686 | 6973686 | 5228239 (7.19e-04) | | 1548. | Otub1 | P < 7.204e-04 | 6871295 | 6871295 | 5254039 (7.20e-04) | | 1549. | BC056923 | P < 7.219e-04 | 6751549 | 6751549 | 4638164 (7.22e-04) | | 1550. | Adora1 | P < 7.226e-04 | 6762353 | 6762353 | 5174154 (7.23e-04) | | 1551. | Pltp | P < 7.230e-04 | 6892964 | 6892964 | 5522347 (7.23e-04) | | 1552. | Klf8 | P < 7.242e-04 | 7014637 | 7014637 | 5434674 (7.24e-04) | | 1553. | Mical1 | P < 7.242e-04 | 6767385 | 6767385 | 5579273 (7.24e-04) | | 1554. | Prkag2 | P < 7.244e-04 | 6936760 | 6936760 | 5476651 (7.24e-04) | | 1555. | Cd37 | P < 7.245e-04 | 6966960 | 6966960 | 4405585 (7.24e-04) | | 1556. | Snx26 | P < 7.249e-04 | 6966292 | 6966292 | 4548957 (7.25e-04) | | 1557. | Gorasp2 | P < 7.254e-04 | 6877964 | 6877964 | 4405986 (7.25e-04) | | 1558. | Sdc4 | P < 7.283e-04 | 6892905 | 6892905 | 4366594 (7.28e-04) | | 1559. | Exoc7 | P < 7.288e-04 | 6792506 | 6792506 | 5225201 (7.29e-04) | | 1560. | Eps8 | P < 7.293e-04 | 6957789 | 6957789 | 4949829 (7.29e-04) | | 1561. | Chn1 | P < 7.315e-04 | 6887854 | 6887854 | 5034467 (7.32e-04) | | 1562. | NA | P < 7.341e-04 | 6937051 | 6937051 | 4889439 (7.34e-04) | | 1563. | Eif3d | P < 7.344e-04 | 6836983 | 6836983 | 5243913 (7.34e-04) | | 1564. | Hells | P < 7.348e-04 | 6869577 | 6869577 | 4809968 (7.35e-04) | | 1565. | D6Wsu176e | P < 7.378e-04 | 6952097 | 6952097 | 5382517 (7.38e-04) | | 1566. | Psat1 | P < 7.436e-04 | 6871768 | 6871768 | 4357131 (7.44e-04) | | 1567. | Pop5 | P < 7.439e-04 | 6933652 | 6933652 | 5488116 (7.44e-04) | | 1568. | Cnih2 | P < 7.448e-04 | 6871076 | 6871076 | 4901324 (7.45e-04) | | 1569. | Cideb | P < 7.488e-04 | 6824842 | 6824842 | 4511180 (7.49e-04) | | 1570. | Dgat2 | P < 7.511e-04 | 6969693 | 6969693 | 5216308 (7.51e-04) | | 1571. | Carm1 | P < 7.512e-04 | 6987391 | 6987391 | 5108840 (7.51e-04) | | 1572. | Exosc10 | P < 7.520e-04 | 6918719 | 6918719 | 5141981 (7.52e-04) | | 1573. | NA | P < 7.537e-04 | 6794063 | 6794063 | 4482066 (7.54e-04) | | 1574. | Rap1b | P < 7.551e-04 | 6777355 | 6777355 | 5202251 (7.55e-04) | | 1575. | Stag3 | P < 7.561e-04 | 6935128 | 6935128 | 4776159 (7.56e-04) | | 1576. | Pthlh | P < 7.593e-04 | 6958326 | 6958326 | 5086240 (7.59e-04) | | 1577. | Hap1 | P < 7.610e-04 | 6791418 | 6791418 | 4784832 (7.61e-04) | | 1578. | Clstn1 | P < 7.612e-04 | 6918814 | 6918814 | 4991052 (7.61e-04) | | 1579. | Wdr36 | P < 7.619e-04 | 6859837 | 6859837 | 4341886 (7.62e-04) | | 1580. | Grik4 | P < 7.622e-04 | 6994947 | 6994947 | 5065836 (7.62e-04) | | 1581. | Rnf123 | P < 7.653e-04 | 6998667 | 6998667 | 5575236 (7.65e-04) | | 1582. | Csf1 | P < 7.665e-04 | 6908048 | 6908048 | 5110752 (7.66e-04) | | 1583. | Usp12 | P < 7.667e-04 | 6943093 | 6943093 | 5111345 (7.67e-04) | | 1584. | Bet1l | P < 7.668e-04 | 6972153 | 6972153 | 5426507 (7.67e-04) | | 1585. | Hn1l | P < 7.685e-04 | 6854401 | 6854401 | 4830592 (7.68e-04) | | 1586. | 6720467C03Rik | P < 7.688e-04 | 6919748 | 6919748 | 5321086 (7.69e-04) | | 1587. | Fbxl2 | P < 7.708e-04 | 6999097 | 6999097 | 5030452 (7.71e-04) | 4704592 (8.32e-04) | | 1588. | Wdr46 | P < 7.716e-04 | 6849992 | 6849992 | 5304856 (7.72e-04) | | 1589. | Calu | P < 7.730e-04 | 6944997 | 6944997 | 4504554 (7.73e-04) | | 1590. | Rps15a | P < 7.732e-04 | 6970821 | 6970821 | 4455811 (7.73e-04) | | 1591. | Atpaf2 | P < 7.762e-04 | 6788743 | 6788743 | 5484777 (7.76e-04) | | 1592. | Jag1 | P < 7.764e-04 | 6891336 | 6891336 | 5579298 (7.76e-04) | | 1593. | Nt5dc2 | P < 7.853e-04 | 6817970 | 6817970 | 5433385 (7.85e-04) | | 1594. | Igf2 | P < 7.865e-04 | 6972317 | 6972317 | 5066197 (7.87e-04) | | 1595. | Igfbp1 | P < 7.872e-04 | 6778609 | 6778609 | 4937206 (7.87e-04) | | 1596. | S3-12 | P < 7.907e-04 | 6856201 | 6856201 | 4658718 (7.91e-04) | | 1597. | Myo7b | P < 7.917e-04 | 6864370 | 6864370 | 4758314 (7.92e-04) | | 1598. | Usp8 | P < 7.925e-04 | 6880945 | 6880945 | 5148739 (7.92e-04) | | 1599. | NA | P < 7.927e-04 | 6808948 | 6808948 | 5096647 (7.93e-04) | | 1600. | Guf1 | P < 7.937e-04 | 6931453 | 6931453 | 4616082 (7.94e-04) | | 1601. | Slc13a4 | P < 7.972e-04 | 6952679 | 6952679 | 4909647 (7.97e-04) | | 1602. | NA | P < 7.973e-04 | 6960390 | 6960390 | 4786864 (7.97e-04) | | 1603. | Apip | P < 7.986e-04 | 6879599 | 6879599 | 4570750 (7.99e-04) | | 1604. | Apeh | P < 8.000e-04 | 6998668 | 6998668 | 4831287 (8.00e-04) | | 1605. | Sesn1 | P < 8.010e-04 | 6767402 | 6767402 | 5606483 (8.01e-04) | | 1606. | Ppap2c | P < 8.017e-04 | 6775298 | 6775298 | 4532447 (8.02e-04) | | 1607. | Cic | P < 8.047e-04 | 6959198 | 6959198 | 4715728 (8.05e-04) | | 1608. | Tesk2 | P < 8.067e-04 | 6916586 | 6916586 | 4510176 (8.07e-04) | | 1609. | Ctdspl | P < 8.067e-04 | 6992864 | 6992864 | 4906515 (8.07e-04) | | 1610. | Ifrd2 | P < 8.073e-04 | 6992229 | 6992229 | 4654289 (8.07e-04) | | 1611. | Dhx16 | P < 8.085e-04 | 6850200 | 6850200 | 5525246 (8.08e-04) | | 1612. | Lrsam1 | P < 8.089e-04 | 6885919 | 6885919 | 4411327 (8.09e-04) | | 1613. | Pdhb | P < 8.101e-04 | 6822459 | 6822459 | 4668550 (8.10e-04) | | 1614. | Bzw2 | P < 8.108e-04 | 6800082 | 6800082 | 4447077 (8.11e-04) | | 1615. | Lamc1 | P < 8.117e-04 | 6763132 | 6763132 | 4482558 (8.12e-04) | | 1616. | Speg | P < 8.119e-04 | 6750628 | 6750628 | 4790184 (8.12e-04) | | 1617. | Adck2 | P < 8.129e-04 | 6945623 | 6945623 | 5217656 (8.13e-04) | | 1618. | Lgtn | P < 8.138e-04 | 6753033 | 6753033 | 4483226 (8.14e-04) | | 1619. | Ubqln4 | P < 8.154e-04 | 6899144 | 6899144 | 5457944 (8.15e-04) | | 1620. | Clic4 | P < 8.165e-04 | 6926072 | 6926072 | 4603054 (8.17e-04) | | 1621. | St13 | P < 8.172e-04 | 6837279 | 6837279 | 4916025 (8.17e-04) | | 1622. | Nkx2-3 | P < 8.183e-04 | 6869879 | 6869879 | 5322721 (8.18e-04) | | 1623. | A830007P12Rik | P < 8.188e-04 | 6885375 | 6885375 | 5515296 (8.19e-04) | | 1624. | Mllt6 | P < 8.190e-04 | 6783917 | 6783917 | 4393846 (8.19e-04) | | 1625. | Pik3r6 | P < 8.190e-04 | 6781927 | 6781927 | 5352718 (8.19e-04) | | 1626. | Nme3 | P < 8.207e-04 | 6849327 | 6849327 | 5296752 (8.21e-04) | | 1627. | Sparc | P < 8.208e-04 | 6788410 | 6788410 | 4803234 (8.21e-04) | | 1628. | Gmfb | P < 8.241e-04 | 6824291 | 6824291 | 4990534 (8.24e-04) | | 1629. | Arhgap18 | P < 8.252e-04 | 6766772 | 6766772 | 4321541 (8.25e-04) | | 1630. | Sf1 | P < 8.274e-04 | 6867854 | 6867854 | 4592148 (8.27e-04) | | 1631. | Tesk1 | P < 8.280e-04 | 6913009 | 6913009 | 5334186 (8.28e-04) | | 1632. | Poln | P < 8.280e-04 | 6937269 | 6937269 | 5389295 (8.28e-04) | | 1633. | Nat10 | P < 8.282e-04 | 6889301 | 6889301 | 4425070 (8.28e-04) | | 1634. | 3300001P08Rik | P < 8.297e-04 | 6790940 | 6790940 | 4791498 (8.30e-04) | | 1635. | NA | P < 8.298e-04 | 6797969 | 6797969 | 4913980 (8.30e-04) | | 1636. | Hspa4 | P < 8.301e-04 | 6788264 | 6788264 | 4448085 (8.30e-04) | | 1637. | Supv3l1 | P < 8.315e-04 | 6774395 | 6774395 | 4376444 (8.32e-04) | | 1638. | Rasgrp4 | P < 8.334e-04 | 6959474 | 6959474 | 4492183 (8.33e-04) | | 1639. | Pmm1 | P < 8.341e-04 | 6837328 | 6837328 | 4876724 (8.34e-04) | | 1640. | Dlst | P < 8.356e-04 | 6796658 | 6796658 | 5514083 (8.36e-04) | | 1641. | Orc1l | P < 8.357e-04 | 6916190 | 6916190 | 4896095 (8.36e-04) | | 1642. | Atm | P < 8.407e-04 | 6995711 | 6995711 | 5243827 (8.41e-04) | 5359525 (9.32e-04) | | 1643. | Ptprm | P < 8.416e-04 | 6856756 | 6856756 | 4347420 (8.42e-04) | | 1644. | Clic6 | P < 8.436e-04 | 6843244 | 6843244 | 5616039 (8.44e-04) | | 1645. | Dlc1 | P < 8.444e-04 | 6981757 | 6981757 | 4740147 (8.44e-04) | | 1646. | NA | P < 8.450e-04 | 6787469 | 6787469 | 5274744 (8.45e-04) | | 1647. | AI314180 | P < 8.512e-04 | 6922021 | 6922021 | 4816959 (8.51e-04) | | 1648. | Psmc3ip | P < 8.522e-04 | 6791465 | 6791465 | 5066381 (8.52e-04) | | 1649. | Coq4 | P < 8.557e-04 | 6876022 | 6876022 | 4951448 (8.56e-04) | | 1650. | Glis2 | P < 8.575e-04 | 6839052 | 6839052 | 4540341 (8.57e-04) | | 1651. | Kcnn4 | P < 8.593e-04 | 6959116 | 6959116 | 5421167 (8.59e-04) | | 1652. | NA | P < 8.598e-04 | 6912945 | 6912945 | 5424200 (8.60e-04) | 5510913 (9.62e-04) | | 1653. | Cul4a | P < 8.608e-04 | 6974137 | 6974137 | 5490931 (8.61e-04) | | 1654. | Stk11ip | P < 8.612e-04 | 6750642 | 6750642 | 5429979 (8.61e-04) | | 1655. | Ptpn9 | P < 8.612e-04 | 6989360 | 6989360 | 5332263 (8.61e-04) | | 1656. | Tada3l | P < 8.627e-04 | 6956552 | 6956552 | 5319257 (8.63e-04) | | 1657. | Rhbdl3 | P < 8.635e-04 | 6782814 | 6782814 | 4579612 (8.63e-04) | | 1658. | Tacstd1 | P < 8.637e-04 | 6852882 | 6852882 | 4991379 (8.64e-04) | | 1659. | Il15ra | P < 8.662e-04 | 6875039 | 6875039 | 4475567 (8.66e-04) | | 1660. | Slc25a13 | P < 8.673e-04 | 6951440 | 6951440 | 5328475 (8.67e-04) | | 1661. | Syngr3 | P < 8.678e-04 | 6854383 | 6854383 | 4333222 (8.68e-04) | | 1662. | Fxyd7 | P < 8.680e-04 | 6966338 | 6966338 | 5506951 (8.68e-04) | | 1663. | Pik3cb | P < 8.681e-04 | 6998192 | 6998192 | 4318633 (8.68e-04) | | 1664. | Slc45a3 | P < 8.698e-04 | 6753089 | 6753089 | 4614536 (8.70e-04) | | 1665. | Gjb2 | P < 8.703e-04 | 6824932 | 6824932 | 4331368 (8.70e-04) | | 1666. | Rad23a | P < 8.708e-04 | 6983878 | 6983878 | 4609778 (8.71e-04) | | 1667. | Mpdu1 | P < 8.717e-04 | 6789324 | 6789324 | 4313255 (8.72e-04) | | 1668. | Mlxipl | P < 8.717e-04 | 6934897 | 6934897 | 5250542 (8.72e-04) | | 1669. | Slc7a8 | P < 8.728e-04 | 6824763 | 6824763 | 5023459 (8.73e-04) | | 1670. | Nanos1 | P < 8.740e-04 | 6870880 | 6870880 | 4942249 (8.74e-04) | | 1671. | Myl4 | P < 8.799e-04 | 6784526 | 6784526 | 5281005 (8.80e-04) | | 1672. | Ppwd1 | P < 8.817e-04 | 6815708 | 6815708 | 4312560 (8.82e-04) | | 1673. | Slc23a3 | P < 8.822e-04 | 6759773 | 6759773 | 5001090 (8.82e-04) | | 1674. | Ccdc42 | P < 8.836e-04 | 6781933 | 6781933 | 4732304 (8.84e-04) | | 1675. | BC003266 | P < 8.838e-04 | 6917219 | 6917219 | 5570364 (8.84e-04) | | 1676. | Paics | P < 8.838e-04 | 6931835 | 6931835 | 4708529 (8.84e-04) | | 1677. | Rab6b | P < 8.844e-04 | 6992012 | 6992012 | 5309353 (8.84e-04) | | 1678. | Fh1 | P < 8.856e-04 | 6764351 | 6764351 | 4876168 (8.86e-04) | | 1679. | Mobkl2a | P < 8.857e-04 | 6775391 | 6775391 | 5485735 (8.86e-04) | | 1680. | Avpi1 | P < 8.862e-04 | 6873171 | 6873171 | 5488689 (8.86e-04) | | 1681. | Psmd10 | P < 8.864e-04 | 7019829 | 7019829 | 4448307 (8.86e-04) | | 1682. | Rdx | P < 8.864e-04 | 6989100 | 6989100 | 4855471 (8.86e-04) | | 1683. | Fndc3a | P < 8.865e-04 | 6825853 | 6825853 | 4325110 (8.86e-04) | | 1684. | Lrrc57 | P < 8.877e-04 | 6890308 | 6890308 | 5306615 (8.88e-04) | | 1685. | Ankrd46 | P < 8.893e-04 | 6835001 | 6835001 | 4938171 (8.89e-04) | | 1686. | Rbpjl | P < 8.898e-04 | 6883094 | 6883094 | 4635781 (8.90e-04) | | 1687. | Epha1 | P < 8.914e-04 | 6953128 | 6953128 | 5523852 (8.91e-04) | | 1688. | Ndrg1 | P < 8.915e-04 | 6836358 | 6836358 | 4328407 (8.92e-04) | | 1689. | Rpl10a | P < 8.917e-04 | 6849543 | 6849543 | 5085396 (8.92e-04) | | 1690. | 2500003M10Rik | P < 8.920e-04 | 6906902 | 6906902 | 4368787 (8.92e-04) | | 1691. | Gipc1 | P < 8.920e-04 | 6977682 | 6977682 | 4722565 (8.92e-04) | | 1692. | Tle2 | P < 8.938e-04 | 6769305 | 6769305 | 5553987 (8.94e-04) | | 1693. | Tmem49 | P < 8.939e-04 | 6790508 | 6790508 | 4780502 (8.94e-04) | | 1694. | Pgm2 | P < 8.971e-04 | 6915791 | 6915791 | 5301549 (8.97e-04) | | 1695. | Amfr | P < 8.983e-04 | 6984413 | 6984413 | 5241077 (8.98e-04) | | 1696. | 2400003C14Rik | P < 8.998e-04 | 6985263 | 6985263 | 4782140 (9.00e-04) | | 1697. | NA | P < 9.012e-04 | 6972989 | 6972989 | 5002277 (9.01e-04) | | 1698. | Itgb7 | P < 9.018e-04 | 6838716 | 6838716 | 4879889 (9.02e-04) | | 1699. | Banp | P < 9.041e-04 | 6979607 | 6979607 | 5180895 (9.04e-04) | 5293058 (1.01e-03) | | 1700. | Rg9mtd1 | P < 9.044e-04 | 6846393 | 6846393 | 5011676 (9.04e-04) | | 1701. | Specc1l | P < 9.047e-04 | 6768851 | 6768851 | 5295510 (9.05e-04) | | 1702. | Dock3 | P < 9.049e-04 | 6998603 | 6998603 | 5077246 (9.05e-04) | 5213595 (9.45e-04) | | 1703. | Lass5 | P < 9.058e-04 | 6838483 | 6838483 | 4921246 (9.06e-04) | | 1704. | Plxdc2 | P < 9.074e-04 | 6875214 | 6875214 | 4907230 (9.07e-04) | | 1705. | Fmn1 | P < 9.108e-04 | 6880087 | 6880087 | 4325867 (9.11e-04) | | 1706. | Ppm1k | P < 9.178e-04 | 6953920 | 6953920 | 5581546 (9.18e-04) | | 1707. | Derl3 | P < 9.183e-04 | 6768891 | 6768891 | 4970000 (9.18e-04) | | 1708. | Ubr5 | P < 9.185e-04 | 6835065 | 6835065 | 5507096 (9.19e-04) | 5613200 (1.05e-03) | | 1709. | Eif3b | P < 9.214e-04 | 6935273 | 6935273 | 5203246 (9.21e-04) | | 1710. | Tenc1 | P < 9.215e-04 | 6833387 | 6833387 | 5505053 (9.21e-04) | | 1711. | Tmem209 | P < 9.223e-04 | 6952426 | 6952426 | 4605127 (9.22e-04) | | 1712. | Prkci | P < 9.226e-04 | 6896518 | 6896518 | 4609746 (9.23e-04) | | 1713. | Rybp | P < 9.236e-04 | 6955978 | 6955978 | 4368347 (9.24e-04) | | 1714. | Tcf25 | P < 9.240e-04 | 6979701 | 6979701 | 4840744 (9.24e-04) | | 1715. | Sh2d3c | P < 9.245e-04 | 6876217 | 6876217 | 5045033 (9.24e-04) | | 1716. | Tjp1 | P < 9.249e-04 | 6967914 | 6967914 | 4729176 (9.25e-04) | | 1717. | Zfp277 | P < 9.259e-04 | 6800236 | 6800236 | 5224422 (9.26e-04) | | 1718. | NA | P < 9.264e-04 | 6990509 | 6990509 | 4574863 (9.26e-04) | | 1719. | Pah | P < 9.274e-04 | 6769593 | 6769593 | 5008746 (9.27e-04) | | 1720. | Parn | P < 9.316e-04 | 6844072 | 6844072 | 4652215 (9.32e-04) | | 1721. | Emid1 | P < 9.323e-04 | 6785698 | 6785698 | 4749999 (9.32e-04) | | 1722. | Stx1b | P < 9.342e-04 | 6971403 | 6971403 | 5200181 (9.34e-04) | | 1723. | Ddx5 | P < 9.354e-04 | 6791965 | 6791965 | 4803068 (9.35e-04) | | 1724. | Snd1 | P < 9.356e-04 | 6944952 | 6944952 | 5259445 (9.36e-04) | | 1725. | NA | P < 9.368e-04 | 6970420 | 6970420 | 4365626 (9.37e-04) | | 1726. | Ppfibp1 | P < 9.371e-04 | 6951118 | 6951118 | 5507222 (9.37e-04) | | 1727. | F5 | P < 9.380e-04 | 6754691 | 6754691 | 4441320 (9.38e-04) | | 1728. | Ctbp2 | P < 9.398e-04 | 6971714 | 6971714 | 4644396 (9.40e-04) | | 1729. | Lime1 | P < 9.399e-04 | 6884267 | 6884267 | 4487143 (9.40e-04) | | 1730. | Moxd1 | P < 9.414e-04 | 6766605 | 6766605 | 5012065 (9.41e-04) | | 1731. | Esrrb | P < 9.426e-04 | 6796728 | 6796728 | 4536772 (9.43e-04) | | 1732. | Ripk5 | P < 9.455e-04 | 6753126 | 6753126 | 4764133 (9.45e-04) | | 1733. | Twistnb | P < 9.457e-04 | 6794339 | 6794339 | 5012977 (9.46e-04) | | 1734. | Atp11c | P < 9.473e-04 | 7017156 | 7017156 | 5263094 (9.47e-04) | | 1735. | Vps35 | P < 9.476e-04 | 6983922 | 6983922 | 4991325 (9.48e-04) | | 1736. | Alk | P < 9.489e-04 | 6857106 | 6857106 | 5047882 (9.49e-04) | | 1737. | Phospho1 | P < 9.492e-04 | 6783784 | 6783784 | 4484044 (9.49e-04) | | 1738. | Sobp | P < 9.511e-04 | 6773582 | 6773582 | 5401867 (9.51e-04) | | 1739. | Zdhhc17 | P < 9.528e-04 | 6776978 | 6776978 | 4379671 (9.53e-04) | | 1740. | Hspa12b | P < 9.555e-04 | 6881237 | 6881237 | 5553093 (9.56e-04) | | 1741. | Ezh1 | P < 9.573e-04 | 6791472 | 6791472 | 4688729 (9.57e-04) | | 1742. | Mtf1 | P < 9.589e-04 | 6917046 | 6917046 | 5079042 (9.59e-04) | | 1743. | Tanc1 | P < 9.609e-04 | 6877441 | 6877441 | 5093525 (9.61e-04) | | 1744. | Tlcd1 | P < 9.623e-04 | 6782679 | 6782679 | 5547434 (9.62e-04) | | 1745. | A930041I02Rik | P < 9.640e-04 | 6886203 | 6886203 | 5263040 (9.64e-04) | | 1746. | Chd4 | P < 9.645e-04 | 6949847 | 6949847 | 5614102 (9.64e-04) | | 1747. | Nek9 | P < 9.673e-04 | 6802386 | 6802386 | 4821231 (9.67e-04) | | 1748. | Slc13a3 | P < 9.706e-04 | 6893002 | 6893002 | 4812514 (9.71e-04) | | 1749. | Slc5a6 | P < 9.708e-04 | 6937056 | 6937056 | 4652192 (9.71e-04) | | 1750. | Farsa | P < 9.718e-04 | 6977766 | 6977766 | 4364462 (9.72e-04) | | 1751. | Upk3b | P < 9.724e-04 | 6934969 | 6934969 | 4751068 (9.72e-04) | | 1752. | Gart | P < 9.729e-04 | 6847880 | 6847880 | 4980144 (9.73e-04) | | 1753. | Fcho1 | P < 9.732e-04 | 6983286 | 6983286 | 5079830 (9.73e-04) | | 1754. | Col4a1 | P < 9.738e-04 | 6980364 | 6980364 | 4453602 (9.74e-04) | | 1755. | Sall1 | P < 9.747e-04 | 6984093 | 6984093 | 4956098 (9.75e-04) | | 1756. | Picalm | P < 9.769e-04 | 6962483 | 6962483 | 4930128 (9.77e-04) | | 1757. | Amz2 | P < 9.779e-04 | 6784785 | 6784785 | 4438771 (9.78e-04) | | 1758. | Trerf1 | P < 9.780e-04 | 6850763 | 6850763 | 4744534 (9.78e-04) | | 1759. | Sbno2 | P < 9.784e-04 | 6775337 | 6775337 | 5477439 (9.78e-04) | | 1760. | Mt3 | P < 9.815e-04 | 6978286 | 6978286 | 4781946 (9.81e-04) | | 1761. | Pisd | P < 9.824e-04 | 6937190 | 6937190 | 4706055 (9.82e-04) | | 1762. | Igtp | P < 9.830e-04 | 6781248 | 6781248 | 5354610 (9.83e-04) | | 1763. | Ankrd52 | P < 9.835e-04 | 6771655 | 6771655 | 5019449 (9.83e-04) | | 1764. | Nek3 | P < 9.850e-04 | 6980943 | 6980943 | 4609166 (9.85e-04) | | 1765. | Mobp | P < 9.861e-04 | 6992925 | 6992925 | 5259385 (9.86e-04) | | 1766. | Cidec | P < 9.868e-04 | 6956558 | 6956558 | 4768683 (9.87e-04) | | 1767. | Ccnb2 | P < 9.868e-04 | 6996646 | 6996646 | 4377292 (9.87e-04) | | 1768. | Itgbl1 | P < 9.872e-04 | 6822367 | 6822367 | 4329717 (9.87e-04) | | 1769. | 3110048E14Rik | P < 9.879e-04 | 6832343 | 6832343 | 4488845 (9.88e-04) | | 1770. | Hmgcs1 | P < 9.880e-04 | 7005797 | 7005797 | 4539582 (9.88e-04) | | 1771. | Scyl2 | P < 9.892e-04 | 6775888 | 6775888 | 5223150 (9.89e-04) | | 1772. | Usp49 | P < 9.903e-04 | 6850791 | 6850791 | 4461687 (9.90e-04) | | 1773. | Arpc1a | P < 9.906e-04 | 6935486 | 6935486 | 4607959 (9.91e-04) | | 1774. | Rb1 | P < 9.908e-04 | 6825875 | 6825875 | 4884886 (9.91e-04) | | 1775. | Nab2 | P < 9.910e-04 | 6777960 | 6777960 | 5095465 (9.91e-04) | 5135561 (1.06e-03) | | 1776. | Asph | P < 9.924e-04 | 6919596 | 6919596 | 4344974 (9.92e-04) | | 1777. | Gla | P < 9.931e-04 | 7019519 | 7019519 | 4600077 (9.93e-04) | | 1778. | BC052040 | P < 9.943e-04 | 6880219 | 6880219 | 5276639 (9.94e-04) | | 1779. | Lrpprc | P < 9.966e-04 | 6857810 | 6857810 | 5466689 (9.97e-04) | | 1780. | Creg1 | P < 9.978e-04 | 6754798 | 6754798 | 4433211 (9.98e-04) | | 1781. | Pot1a | P < 9.979e-04 | 6952231 | 6952231 | 5214339 (9.98e-04) | | 1782. | Csrnp2 | P < 9.982e-04 | 6838563 | 6838563 | 4846478 (9.98e-04) | | 1783. | NA | P < 9.996e-04 | 6984955 | 6984955 | 4654217 (1.00e-03) | | 1784. | Alkbh3 | P < 9.997e-04 | 6888931 | 6888931 | 5194673 (1.00e-03) | | 1785. | Sdc1 | P < 1.000e-03 | 6793226 | 6793226 | 5299552 (1.00e-03) | | 1786. | Usp9x | P < 1.001e-03 | 7010073 | 7010073 | 5578807 (1.00e-03) | | 1787. | Sema6c | P < 1.004e-03 | 6899654 | 6899654 | 4937453 (1.00e-03) | | 1788. | Masp1 | P < 1.004e-03 | 6844632 | 6844632 | 5012634 (1.00e-03) | | 1789. | Fbn2 | P < 1.004e-03 | 6865792 | 6865792 | 5363384 (1.00e-03) | | 1790. | Asb6 | P < 1.005e-03 | 6885793 | 6885793 | 4488665 (1.01e-03) | | 1791. | Tmeff1 | P < 1.005e-03 | 6913348 | 6913348 | 4685318 (1.01e-03) | | 1792. | 1810043G02Rik | P < 1.006e-03 | 6769030 | 6769030 | 4335503 (1.01e-03) | | 1793. | Pole | P < 1.008e-03 | 6933422 | 6933422 | 5382018 (1.01e-03) | | 1794. | Mid1 | P < 1.009e-03 | 7015255 | 7015255 | 4706205 (1.01e-03) | | 1795. | Pfkfb4 | P < 1.011e-03 | 6992380 | 6992380 | 4993802 (1.01e-03) | | 1796. | Myst2 | P < 1.011e-03 | 6791023 | 6791023 | 5180504 (1.01e-03) | | 1797. | Tsr1 | P < 1.012e-03 | 6782411 | 6782411 | 5534238 (1.01e-03) | | 1798. | Coro7 | P < 1.013e-03 | 6843653 | 6843653 | 4564519 (1.01e-03) | | 1799. | Gabpb1 | P < 1.013e-03 | 6890638 | 6890638 | 4404263 (1.01e-03) | | 1800. | Fads3 | P < 1.016e-03 | 6868018 | 6868018 | 5287089 (1.02e-03) | | 1801. | Sirt2 | P < 1.018e-03 | 6959452 | 6959452 | 4563986 (1.02e-03) | | 1802. | Ooep | P < 1.019e-03 | 6997105 | 6997105 | 4476650 (1.02e-03) | | 1803. | Csnk1d | P < 1.020e-03 | 6792827 | 6792827 | 4693877 (1.02e-03) | | 1804. | NA | P < 1.021e-03 | 6947570 | 6947570 | 5267179 (1.02e-03) | | 1805. | 6530403A03Rik | P < 1.023e-03 | 6806300 | 6806300 | 5382513 (1.02e-03) | | 1806. | Pkp2 | P < 1.023e-03 | 6839681 | 6839681 | 5496065 (1.02e-03) | | 1807. | Abat | P < 1.024e-03 | 6839231 | 6839231 | 5323000 (1.02e-03) | | 1808. | Rhot1 | P < 1.025e-03 | 6782812 | 6782812 | 4540492 (1.02e-03) | | 1809. | Zfp608 | P < 1.027e-03 | 6865643 | 6865643 | 4775517 (1.03e-03) | | 1810. | 1810023F06Rik | P < 1.027e-03 | 6803211 | 6803211 | 4580560 (1.03e-03) | | 1811. | Itpr3 | P < 1.028e-03 | 6849481 | 6849481 | 4477340 (1.03e-03) | | 1812. | Chd3 | P < 1.029e-03 | 6789274 | 6789274 | 4664539 (1.03e-03) | | 1813. | Aph1c | P < 1.033e-03 | 6996438 | 6996438 | 5387095 (1.03e-03) | | 1814. | Sec23a | P < 1.033e-03 | 6801102 | 6801102 | 4902509 (1.03e-03) | | 1815. | Ube3a | P < 1.036e-03 | 6960931 | 6960931 | 4642907 (1.04e-03) | | 1816. | NA | P < 1.037e-03 | 6795593 | 6795593 | 5191416 (1.04e-03) | | 1817. | Atp1a1 | P < 1.039e-03 | 6907638 | 6907638 | 4379552 (1.04e-03) | | 1818. | Gabbr1 | P < 1.040e-03 | 6850271 | 6850271 | 4342621 (1.04e-03) | | 1819. | Klhl25 | P < 1.041e-03 | 6961766 | 6961766 | 4520503 (1.04e-03) | | 1820. | Yaf2 | P < 1.041e-03 | 6838032 | 6838032 | 4456854 (1.04e-03) | | 1821. | Angptl4 | P < 1.041e-03 | 6854971 | 6854971 | 4335852 (1.04e-03) | | 1822. | Ntan1 | P < 1.044e-03 | 6839539 | 6839539 | 4578074 (1.04e-03) | | 1823. | Dusp9 | P < 1.046e-03 | 7011938 | 7011938 | 4592687 (1.05e-03) | | 1824. | Slc38a2 | P < 1.048e-03 | 6838257 | 6838257 | 5277703 (1.05e-03) | | 1825. | Fem1b | P < 1.049e-03 | 6996223 | 6996223 | 4931398 (1.05e-03) | | 1826. | Cpsf6 | P < 1.053e-03 | 6777315 | 6777315 | 5410006 (1.05e-03) | | 1827. | H2afx | P < 1.054e-03 | 6988627 | 6988627 | 5293064 (1.05e-03) | | 1828. | Ovca2 | P < 1.055e-03 | 6789721 | 6789721 | 4481057 (1.05e-03) |**
