# Supplementary material for: Alternative splicing is frequent during early embryonic development in mouse
Source: BMC Genomics. 2010 Jun 23;11:399. doi: 10.1186/1471-2164-11-399 (PMC2898759; doi:10.1186/1471-2164-11-399)
Supplement: Additional file 3 — Figure S2 - PCR validation. Results of the endpoint PCR validation of alternative cassette exons. [file 1471-2164-11-399-S3.PDF]

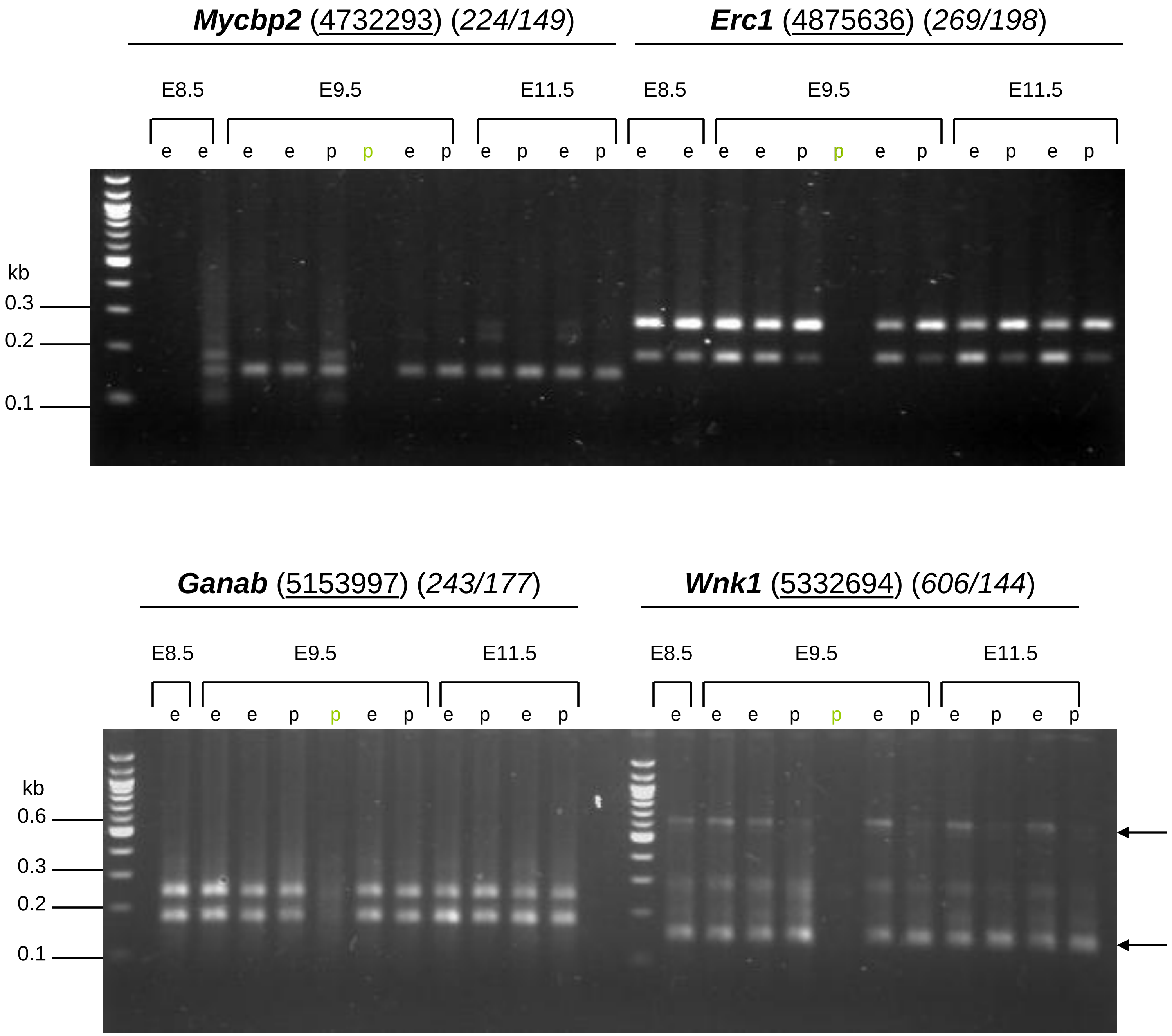

Mycbp2 (4732293) (*224/149*)
Erc1 (4875636) (*269/198*)
E8.5
E9.5
E11.5
e e e e p p e p e p e p
e e e e p p e p e p e p
kb
0.3
0.2
0.1
Ganab (5153997) (*243/177*)
Wnk1 (5332694) (*606/144*)
e e e p p e p e p e p
e e e p p e p e p e p
0.6

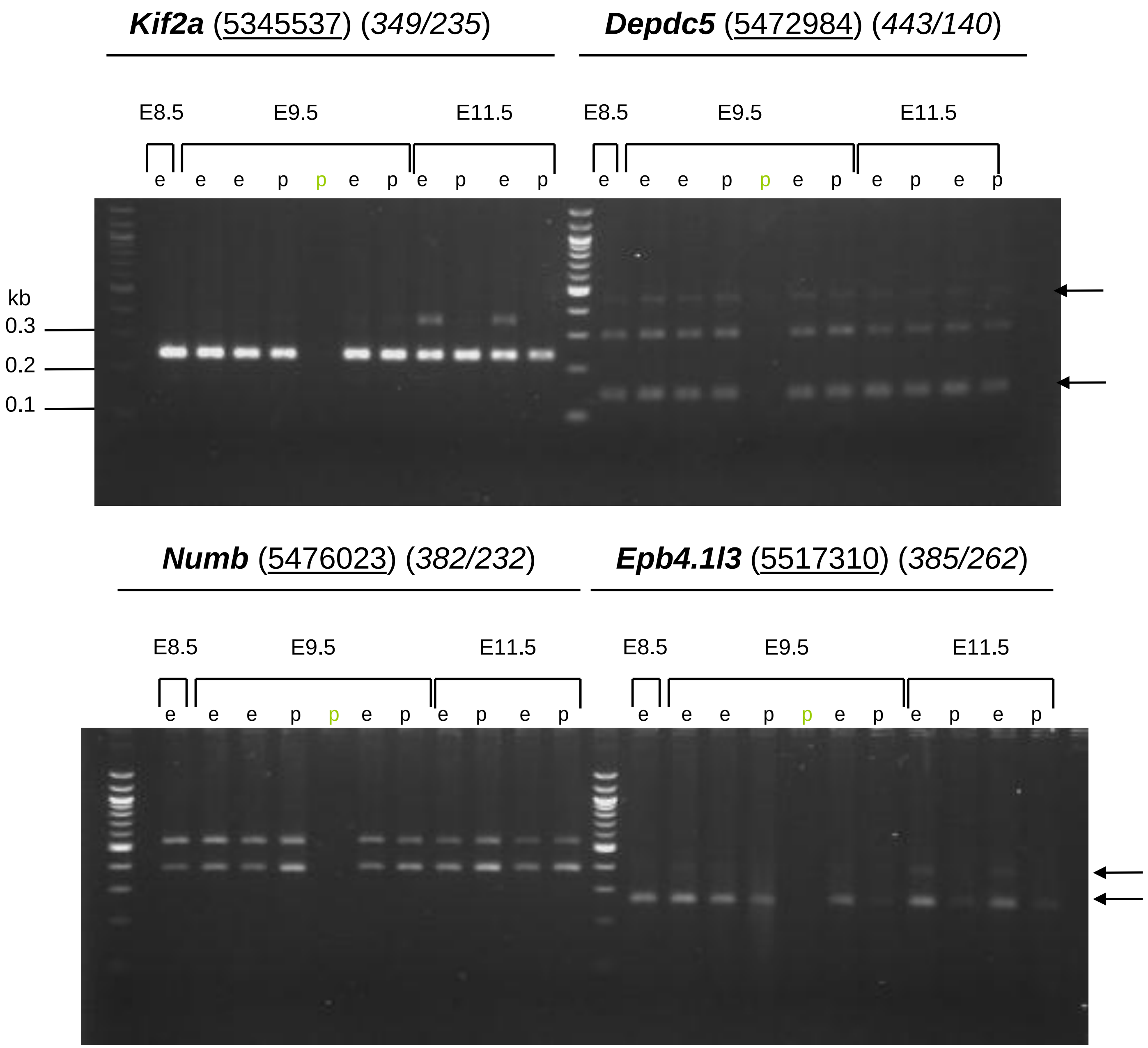

Kif2a (5345537) (*349/235*)
Depdc5 (5472984) (*443/140*)
E8.5 E9.5 E11.5
e e e p p e p e p e p
kb
0.3
0.2
0.1
Numb (5476023) (*382/232*)
Epb4.1l3 (5517310) (*385/262*)

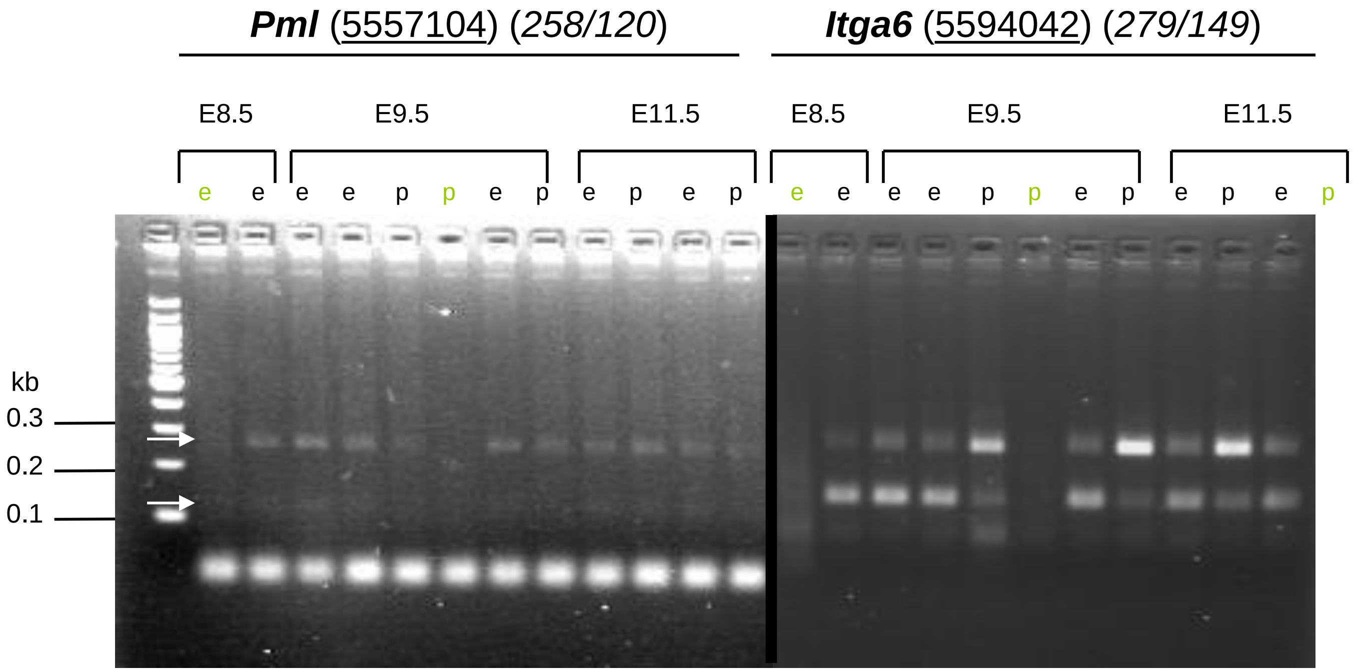

Supplementary figure 2 – Results of the endpoint PCR validation of alternative cassette exons. We can confirm alternative splicing for each of the ten candidates chosen between stages (E8.5-E11.5) or tissues (embryonic (e) or placenta (p)). The gene symbols are indicated above the gel figure in bold italic with their respective candidate probe set underlined and the expected PCR product size of the inclusion/exclusion isoforms in italic. The expected PCR products are also indicated with arrows on the gel image when unclear.
