# Supplementary material for: Alternative splicing is frequent during early embryonic development in mouse
Source: BMC Genomics. 2010 Jun 23;11:399. doi: 10.1186/1471-2164-11-399 (PMC2898759; doi:10.1186/1471-2164-11-399)
Supplement: Additional file 4 — Additional tables . Table S2 Table containing primers used for end-point PCR validation. Table S3 Table containing primers used for qRT-PCR validation. Table S4 Information about binding sites found for Fox2. [file 1471-2164-11-399-S4.DOCX]

| **Gene symbol** | **Forward primer** | **Reverse primer** |
| --- | --- | --- |
| **Itga6** | TCATCCTCCTGGCTGTTCTT | TTGATCTCTCGCTCTTCTTTCC |
| **Erc1** | AGGTGTACAGGAGCCACTCAA | CCCTTTGCTCCTTAGCAGTG |
| **Kif2a** | TGCATCCGAGCCTTAGGTAG | GGTGCATTATTGGATGGACA |
| **Epb4.1L3** | CAAACGAGTGGGAAAAGAGG | AACTTCATCCCTGGCTGTGT |
| **Numb** | GGGATTTCCTGCTCTTAGCC | CCGCACACTCTTTGACACTT |
| **Pml** | GCCTGAGGACCTTGAGCAG | CCTGTCCTCGTTCTCCTCTG |
| **Depdc5** | GAAGCTGCGCGAACTGTG | AAAGCCCTTCTTGTGGATGA |
| **Wnk1** | TCACTTCCACAAGCAGCATC | CGTTGGAAGAGGGAGCAATA |
| **Mycbp2** | CGGTTACACTGAAGCCTGGT | TCTCGTTCTTGCATTGTTGC |
| **Ganab** | GGAGCTAACAGTGGCTGAGG | ATTGCCTTCAGCTGGGTCTT |

**Supplementary table 1 - Primers used for endpoint PCR validation.**

| **Gene symbol** | **Splicing isoform detected** | **Forward primer** | **Reverse primer** |
| --- | --- | --- | --- |
| **Ank3** | Prox. promoter | GCATGTTTCGGTCTGAGTTT | TAGAGTAAGCCGTCCACACC |
|  | Distal promoter | GGAGAGAACACCCAGTTCCT | CTTGAGATCAGGGGAGTTGA |
| **Depdc5** | Exon inclusion | CGAGTCATGGGAGTGCATAA | CAAAGCCCTTCTTGTGGATG |
|  | All isoforms | GGAGACATTGTGGAGATTGC | CCAGGTCAAGAGTCACATCC |
| **Elmo1** | Distal promoter | CGACTTTCACCCGATGTTCT | TCTCAGACTGGCGGATTTTC |
|  | Prox. promoter | TGGCTCAACTGGAAACCTGT | TCTCAGACTGGCGGATTTTC |
| **Erc1** | Exon inclusion | CCGAGACCTGGAAGAGGAA | AGCCGCTCTCTTTTTCAGC |
|  | All isoforms | GGATCCAGAGGGACTTGAAT | TCAATCCTCAGCTCCATTTC |
| **Gcnt2** | Distal promoter | ATGAGCACTTCTGGGTGACA | AATCAGCCACTGCAAGTCTC |
|  | Prox. promoter | TACTTTCAGTCCCGATGAGC | AATCAGCCACTGCAAGTCTC |
| **Itsn1** | Prox. finish | CCAGAACGATGACGAACTAGC | TCACATGATCAAGGCTGCAT |
|  | Distal finish | CCAGAACGATGACGAACTAGC | TCGGGGTCAGCATATCTAAG |
| **Kif1b** | Prox. finish | GCTGCTGTCAAAAGCAGACT | TAGGCACCAACTCAATCCAT |
|  | Distal finish | AGCCTGTTTCAGCATCACTC | AACAAAGGAAGCTGGGAGTC |
| **Kif2a** | Exon inclusion | AAGGAGAAAAGTCGTCATAATCG | GCATCCGAGCCTTAGGTAGA |
|  | All isoforms | CGCGATGTCTTTTTAATGCT | ACGTCCTCCACACACTTCAC |
| **Mycbp2** | Exon inclusion | TTTGGAAGTCCATGAAAATACG | GATGGCGTGTGGTTGAGAC |
|  | All isoforms | TATGGCCTGGGAAATAACAA | TGATGTGGACACTGGTCTTG |
| **Rab6** | Exon1 inclusion | AGCAGAGCGTTGGAAAGAC | TCACGGATGTAACTGGGAAT |
|  | Exon2 inclusion | AGCAGAGCGTTGGAAAGAC | AGGAATCAAGCTCCTGAACC |
| **Tcf4** | Distal promoter | GAAAGGGGCTCATACTCATCTT | TAGGGAAAGTGCTGGCTGCT |
|  | Prox. promoter | GGGCGGCAACTCTTTGAT | TAGGGAAAGTGCTGGCTGCT |
| **Wnk1** | Exon inclusion | CCAGCAGCCATTGTTATAGG | TGAACCTCACATCCCAGTTT |
|  | All isoforms | AGAGTGAGCAGCCAACAGAC | AGATAGAACCCCAGCCAAAC |

Supplementary table 2 - Primers used for quantitative PCR validation.

| Binding site | Intron containing site | Probeset (exon) | Gene symbol |
| --- | --- | --- | --- |
| UGCAUG | Upstream | 5117020 | Add3 |
|  |  | 4866829 | Epb4.1l3 |
|  |  | 4532968 | Fastkd5 |
|  |  | 5066773 | Fyn |
|  |  | 4864482 | Map3k7 |
|  |  | 5427887 | Mbtps1 |
|  |  | 5476023 | Numb |
|  |  | 4538548 | Pbx1 |
|  |  | 4601289 | Prepl |
|  |  | 4321833 | Rufy3 |
|  |  | 5230966 | Tcf7l2 |
|  | Downstream | 4767450 | Col25a1 |
|  |  | 5018323 | Cux1 |
|  |  | 5480180 | Dab2 |
|  |  | 4930679 | Deaf1 |
|  |  | 5382517 | Fam3c |
|  |  | 5201971 | Fn1 |
|  |  | 4954760 | Ipo11 |
|  |  | 4849027 | Mbnl2 |
|  |  | 5021115 | Rab6 |
|  |  | 4900774 | Srpk2 |
|  |  | 4964545 | Tcf7l2 |
| Combined | Upstream | 4690576 | 0610010K14Rik |
|  |  | 5518088 | Acox1 |
|  |  | 5117020 | Add3 |
|  |  | 5599187 | Ank3 |
|  |  | 5434022 | Csda |
|  |  | 5596927 | Ctage5 |
|  |  | 4851097 | Cux1 |
|  |  | 5256610 | Dnajc6 |
|  |  | 4749999 | Emid1 |
|  |  | 4866829 | Epb4.1l3 |
|  |  | 4532968 | Fastkd5 |
|  |  | 5066773 | Fyn |
|  |  | 4864482 | Map3k7 |
|  |  | 5427887 | Mbtps1 |
|  |  | 4574272 | Myb |
|  |  | 5476023 | Numb |
|  |  | 5156154 | Pard3 |
|  |  | 4538548 | Pbx1 |
|  |  | 5218867 | Pphln1 |
|  |  | 4601289 | Prepl |
|  |  | 4675762 | Ptk2 |
|  |  | 5073391 | Pus1 |
|  |  | 4456110 | Rai14 |
|  |  | 5422528 | Rbpms |
|  |  | 4465149 | Rnf38 |
|  |  | 4321833 | Rufy3 |
|  |  | 5070994 | Tcf12 |
|  |  | 5230966 | Tcf7l2 |
|  |  | 4949913 | Tnc |
|  |  | 5177372 | Zfp207 |
|  |  | 4552183 | Zfp740 |
|  | Downstream | 4478673 | Enox1 |
|  |  | 4616082 | Guf1 |
|  |  | 4629085 | Mta1 |
|  |  | 4719820 | Ank3 |
|  |  | 4732293 | Mycbp2 |
|  |  | 4767450 | Col25a1 |
|  |  | 4838652 | Smarca2 |
|  |  | 4842548 | Reep6 |
|  |  | 4849027 | Mbnl2 |
|  |  | 4860483 | Phc1 |
|  |  | 4900774 | Srpk2 |
|  |  | 4930679 | Deaf1 |
|  |  | 4954760 | Ipo11 |
|  |  | 4964545 | Tcf7l2 |
|  |  | 4987109 | Abi2 |
|  |  | 5018323 | Cux1 |
|  |  | 5021115 | Rab6 |
|  |  | 5039407 | R3hdm2 |
|  |  | 5049028 | Ank3 |
|  |  | 5074796 | Ank3 |
|  |  | 5076503 | Epb4.1l3 |
|  |  | 5115644 | Itsn1 |
|  |  | 5201971 | Fn1 |
|  |  | 5224672 | Ank3 |
|  |  | 5375456 | Ank3 |
|  |  | 5382517 | Fam3c |
|  |  | 5464083 | Bnc2 |
|  |  | 5480180 | Dab2 |
|  |  | 5551727 | Slain2 |

Supplementary table 3 - List of probe sets representing cassette exons with introns containing a binding site for Fox2.
